# A preliminary investigation into the early embryo death syndrome (EEDS) at the world’s largest green turtle rookery

**DOI:** 10.1371/journal.pone.0195462

**Published:** 2018-04-25

**Authors:** David Terrington Booth, Andrew Dunstan

**Affiliations:** 1 School of Biological Sciences, The University of Queensland, Queensland, Australia; 2 Queensland Government Department of National Parks, Sports and Racing, Brisbane, Queensland, Australia; Deakin University, AUSTRALIA

## Abstract

Raine Island hosts the largest nesting aggregation of green turtles in the world, but nest emergence success and hence recruitment of hatchlings off the beach appear to have significantly declined since the 1990s. Nests destroyed by subsequent nesting turtles, and nest failure due to flooding account for most of the nest failure, but many nests still have poor hatch success even when undisturbed and flood-free. In undisturbed, flood-free nests that experience high mortality, embryos typically die at a very early stage of development, a phenomenon we term early embryo death syndrome (EEDS). Previous research indicates that EEDS is correlated with the number of females nesting at Raine Island during a nesting season. Here, we monitor nest temperature and oxygen (PO_2_) and carbon dioxide (PCO_2_) partial pressures during the first week after nest construction to discover if they are associated with EEDS. Our investigation found that the proportion of early embryo death was greatest in two nests that experienced the highest nest temperature, lowest PO_2_ and highest PCO_2_ during the first week of incubation suggesting that these variables either by themselves or in combination may be the underlying cause of EEDS. These two nests were located adjacent to maturing nests, so the high temperature and more extreme PO_2_s and PCO_2_s are most likely to be caused by the combined metabolism of embryos in the mature nests. Although this conclusion is based on just two nests and needs to be substantiated in future studies, it would appear that the laying of new nests in the close location to mature nests could be a significant cause of hatch failure at high density nesting sea turtle rookeries around the world.

## Introduction

Successful persistence of animal populations requires that sufficient young survive and become breeding adults. In egg-laying amniotes (all birds and most reptiles), it is essential that enough eggs are successfully incubated to produce viable hatchlings that can then be recruited into the adult population. In sea turtles, which are egg laying, embryonic death during incubation varies greatly between populations, and in some populations high embryonic death rates may limiting population growth or even persistence. Raine Island is the location of the world’s largest green turtle (*Chelonia mydas*) nesting aggregation [1] with between 3,600 and 60,000 females visiting to nest in a single nesting season [2]. In the 1970s and 1980s Raine Island was thought to be a highly productive rookery with nest emergence success exceeding 75% in nests located by signs of successful emergence [1] and the population was recently assessed to be increasing during these years [3]. Indeed, a description from February 1975 reads ‘vast numbers of hatchlings were crossing the beach throughout the night into the hours approaching dawn’ [1]. However, during the late 1990s recruitment of hatchling green turtles from Raine Island appeared to be greatly reduced with only 10s to 100s crossing the beach each night in February 1997 despite very large numbers of turtles nesting in December 1996 and it is thought that most of the eggs laid in the 1996/1997 nesting season died before hatching [1]. This apparent decrease in hatchling recruitment since the late 1990s coincided with the appearance of tidal inundation of some of the nesting area, a phenomenon that had not been regularly noted previously [1]. Increased nest inundation may occur if either sand has been lost from the island or that the existing sand has been redistributed from a relatively high but narrow beach to a wider but shallower beach. In either case, the net result has been that large numbers of turtle nests drown during periods of peak high tides or during storm surges [2].

While initial investigations clearly indicated that water inundation does cause significant mortality in green turtle nests at Raine Island, other factors are also causing within nest mortality because many nests that remain flood-free throughout incubation still experience low hatching success [2]. Importantly, the majority of embryonic failure in these undisturbed and ‘dry’ nests occurs at a very early stage of embryonic development before stage 16 described by Miller [4], (when limb buds are forming, death within just 0–7 days of incubation), and the proportion of nests experiencing this early stage mortality is positively correlated with the number of nesting females visiting Raine Island to nest [2]. We term this high early embryonic nest mortality that occurs in dry and undisturbed nests early embryo death syndrome (EEDS). EEDS has also been identified as the major cause of failed nests in the Playa Grande, Costa Rica nesting population of leatherback turtles (*Dermochelys coriacea*) but the cause of EEDS is not known [5,6]. Embryo death is also correlated with nest density in Costa Rican populations of olive ridley turtles (*Lepidochelys olivacea*), but embryo death was independent of developmental stage, i.e. embryo death did not occur predominately within the first week of incubation [7].

Two possible explanations of EEDS are either inviable eggs (eggs are infertile or the eggs are fertile but the embryos have deteriorated while the eggs have been held in the oviducts because of a prolonged storage period due to the female not being able to nest successfully over many nights) or a sub-optimal nest microenvironment. Infertility of eggs is an unlikely explanation, but prolonged retention of eggs in oviducts does occur in green turtles nesting at Raine Island, especially in years with high numbers of nesting females, but it is unknown if the holding of eggs in the uterus for periods longer than a few days causes egg deterioration in green turtles. However, the proportion of eggs experiencing EEDS increased as the inter-nesting period increased in the Playa Grande, Costa Rica nesting population of leatherback turtles, suggesting that increased egg retention time may be detrimental to embryos [5].

A number of factors could be causing unfavourable incubation conditions in green turtle nests at Raine Island and these factors alone or acting together may be responsible for EEDS. These factors include microbial infection, high nest temperature and fatal respiratory gas (oxygen and carbon dioxide) concentrations. In the current study, using field observations, we measure general sand and within nest gaseous conditions and nest temperature to discover if suboptimum levels of oxygen and carbon dioxide and high temperatures are correlated with the prevalence of EEDS.

## Methods

### Ethics statement

All procedures used in this project were approved by the Raine Island scientific advisory group and by a University of Queensland animal ethics committee (approval number SBS/267/17).

### Study area

All measurements were conducted on Raine Island (11^o^ 35’ 25” S, 144^o^ 02’ 05” E) during the 2016–2017 nesting season when an estimated 12,000 females visited the island to nest [2]. Assuming that each female constructs 5 nests (based on recent unpublished data from 20 satellite tagged females nesting at Raine Island), this would result in approximately 72,000 nests being constructed in 2016–2017. Because of the island’s isolation, it was not possible to take gas measurements throughout the incubation period, so measurements were made during three discreet trips from 30-Nov-2016 to 6-Dec-2016, 31-Jan-2017 to 5-Feb-2017 and 5-Apr-2017 to 9-Apr-2017.

### Beach sand gas profiles

On three dates, 2-Dec-2106, 3-Feb-2017 and 6-Apr-2017 oxygen and carbon dioxide at nest depth (55 cm below the sand surface) were measured at ~ 2m intervals across three transects running from the berm at the beach front to a small rock cliff at the back of the beach. At each sampling site, an aluminium tube (outside diameter 8 mm) with a sharpened point, inside of which ran a plastic tube of inside diameter 1.5 mm was inserted 55 cm into the sand. The base of the tube had four, 1 mm diameter holes drilled through it from one side to the other, including through the internal plastic tube for the purpose of sampling sand gas. Once the sampling tube had been insert to 55 cm, a 70 ml sample of gas was aspirated through drierite™ desiccant into a combination oxygen-carbon dioxide analyser (Quantek model 907 O2/CO2 analyzer, Grafton, Massachusetts, USA) via its internal pump. The gas analyser was calibrated daily with a precision 2.00% carbon dioxide calibration gas, and the span of the oxygen meter recorded after each sample was taken. Barometric pressure measured by a shipboard aneroid barometer was recorded within 2 hours of gas samples being taken. Gas concentrations were converted to partial pressures using the measured barometric pressure and assuming the sand gas was saturated with water vapor at a temperature of 30°C.

During the February 2017 trip, two nests NG31 and NG48 consistently experienced respiratory gases more extreme than the other nests that were monitored. On 2-Feb-2017, sand gas profiles in the immediate vicinity of these nests were measured by sampling sand gas at a depth of 55 cm at 20 cm intervals across transects that ran roughly north-south and east-west through these nests.

### Nest monitoring

During the December 2016 and February 2017 field trips a sample of 13 and 19 nests respectively were set up for monitoring. Nests were haphazardly chosen whenever a female close to oviposition was found during the course of female censoring work on a particular night. During oviposition, a ping pong ball with the nest identity number and an iButton (model DS1921H-F5, Maxim Integrated, San Jose, California, USA, precision 0.0625°C, accuracy ± 0.5°C) temperature logger programed to log temperature every hour were placed amongst the eggs as they were being laid. The iButton was placed inside an orange party balloon, the balloon sealed with a knot, and a string tied to the balloon at one end and a protective stake at the other end. An air-stone attached to 2 mm internal diameter plastic tubing was also placed amongst the eggs during egg laying, and the other end of the tube taped to the stake with two 3-way stopcocks attached to it. In December 2016 a single 50 x 50 x 1800 mm wooden stake was inserted 80 cm into the sand, approximately 20 cm behind the nest as the female began filling in her nest. In February 2017, three 50 x 50 x 1800 mm wooden stakes were inserted around the nest to protect it from subsequent nesting females. The position of the nest was recorded via GPS with a hand held GPS unit (Garmin eTrex 30; Kansas, USA, accuracy ± 0.2 m) and mapped (S1 Fig). Nests were visited daily and a 70 ml sample of gas aspirated and analysed as described above. Gas concentrations were converted to partial pressures using the measured barometric pressure and assuming the nest was saturated with water vapor at nest temperature when the samples were taken.

On a subsequent fieldtrip, at a time after the nests were due to hatch, nests were excavated, the iButton temperature data loggers recovered and downloaded, and the hatching and emergence success of the nest recorded by counting the number of hatched shells, and unhatched eggs, and the developmental stage of unhatched eggs visually assessed to determine if death occurred within the first week of incubation before embryos reached stage 16 described by Miller [4].

Data are presented as means ± SE, and analysed using Pearson product-moment correlation and one-way ANOVA and statistical significance assumed if P < 0.05. All statistical procedures were performed using Statistica Ver 13 software (Dell Corporation).

## Results

Of the 32 nests we set up for monitoring, 26 survived to the end of incubation. Within these nests the proportion of early stage embryo death varied between 2 and 79% and nest emergence successes varied between 4 and 95%. As was expected, the proportion of early stage embryo death was negatively correlated with nest emergence success (statistical test preformed on Arcsine transformed data, r = 0.82, t = -7.06, P < 0.001, n = 26). Six nests set up in the December trip were destroyed by subsequent nesting turtles, but the 3 wooden stakes used to protect nests in the February trip were successful in protecting these nests from being dug up by subsequent nesting turtles.

### Sand gas profiles

**Fig 1:**
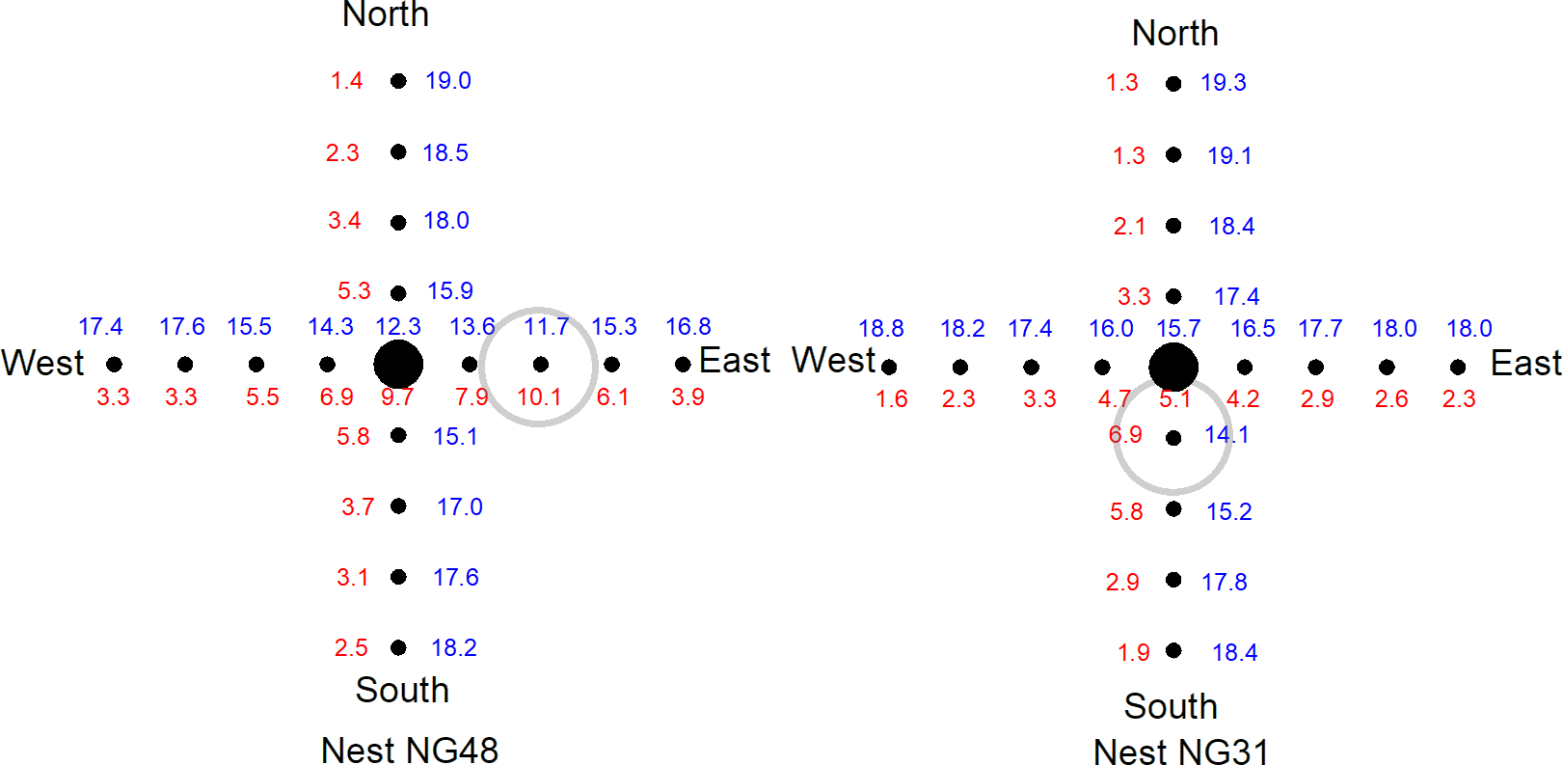
Gas samples taken at 55 cm depth at 20 cm intervals in north-south and east-west transects through nests NG31 and NG48 taken on 2 Feb 2017. Numbers in blue indicate PO_2_, numbers in red indicate PCO_2_. The gas sample that differed the most from atmospheric air are indicated by grey circles suggesting that a mature nests were present at these locations.

Respiratory gases in sand at 55 cm depth sampled in transects were slightly different from atmospheric air in all samples taken (PO_2_ (kPa): Atmospheric air = 20.3; 2 Dec 2016 = 19.8 ± 0.2, range 18.9–19.9, n = 37; 2 Feb 2017 = 18.8 ± 0.2, range 15.0–19.7, n = 32; 6 Apr 2017 = 18.7 ± 0.2, range 15.5–19.8, n = 28; PCO_2_ (kPa): Atmospheric air = 0.1; 2 Dec 2016 = 0.3 ± 0.2, range 0.1–1.0, n = 37; 2 Feb 2017 = 0.9 ± 0.2, range 0.1–5.1, n = 32; 6 Apr 2017 = 0.8 ± 0.2, range 0.1–2.1, n = 28). Mean PO_2_ differed between sampling times (F_2,94_ = 7.37, P = 0.001) with a Tukey Post Hoc test for uneven sample sizes indicating PO_2_ on 3 Feb and 6 Apr were similar, but lower than on 2 Dec. Mean PCO_2_ was not different between sampling dates (F_2,94_ = 1.79, P = 0.172). Sand gas samples taken on 2-Feb-2017 around nest NG31 indicated there was a mature nest located approximately 20 cm south of nest NG31 (Fig 1), while samples taken on the same day around nest NG48 indicated there was a mature nest located approximately 40 cm east of nest NG48 (Fig 1). However, we left the island before hatchlings from these mature nests were predicted to reach the sand surface and thus we would not absolutely confirm the existence of mature nests at these locations.

### Nest gas profiles

**Fig 2:**
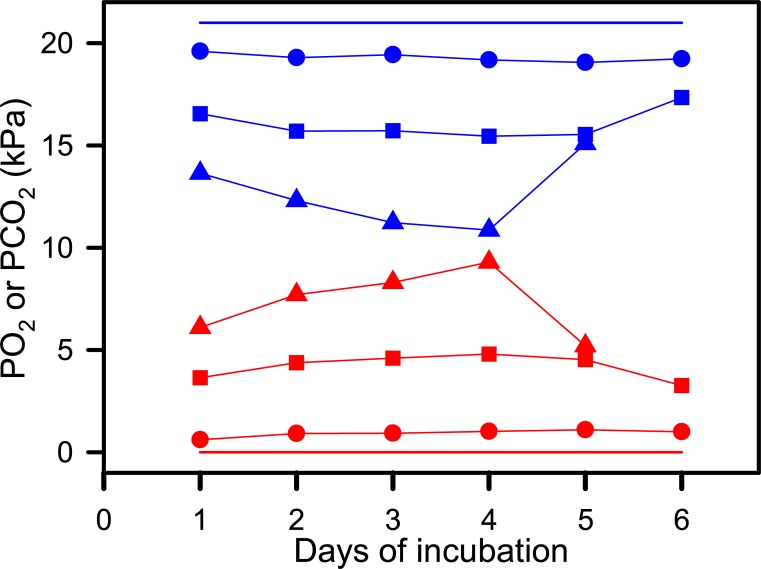
Oxygen partial pressure (PO_2_ blue lines and symbols) and carbon dioxide partial pressure (PCO_2_ red lines and symbols) inside newly constructed green turtle nests. Circles represent the means of 27 nests whose gas partial pressure were similar to air (SE error bars are contained entirely within the symbols). Square symbols represent nest NG31, and triangle symbols represent nest NG48. Straight blue lie indicates PO_2_ of atmospheric air and straight red line indicates PCO_2_ of atmospheric air.

**Fig 3:**
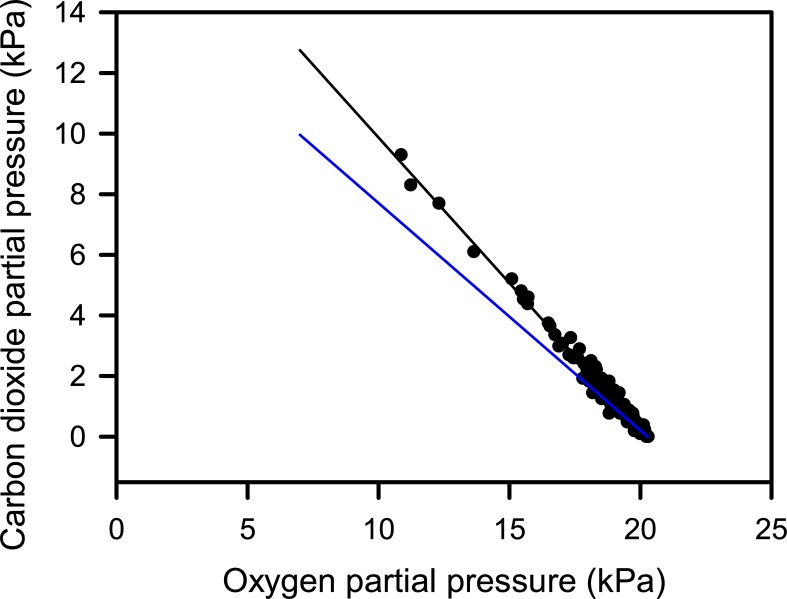
Relationship between oxygen partial pressure and carbon dioxide partial pressure for all nest samples from both the December 2016 and February 2017 field trips, and the theoretical lines on which data should fall if gas exchange between the atmosphere and nest occurred purely by diffusion (black line), or purely by convection (blue line) (see reference [8] for details).

Three nests were destroyed by subsequent nesting turtles within two days of being set up during the December 2016 trip, but protecting nests with 3 wooden stakes insured that all nests setup during the February 2017 trip survived intact. In 24 nests the PO_2_ (19.3 ± 0.2 kPa,) and PCO_2_ (0.9 ± 0.2 kPa) were close to atmospheric air throughout the entire first 6 days of incubation, but in nests NG31 and NG48, PO_2_ was lower and PCO_2_ higher than in atmospheric air (Fig 2). Nest PO_2_ and PCO_2_ were highly negatively correlated (r = -0.99, t = -80.60, P < 0.001, n = 112, Fig 3). On theoretical grounds, it is possible to determine if gas exchange between the atmosphere and the nest was dominated by diffusion or convection from a plot of PCO_2_ against PO_2_ by assuming the respiratory gas exchange quotient of embryos is 0.75 and that the ratio of the CO_2_ diffusion coefficient to O_2_ diffusion coefficient is 0.78 in air [8]. Using this approach it appears that respiratory gas exchange in nests was dominated by diffusion (Fig 3).

**Fig 4:**
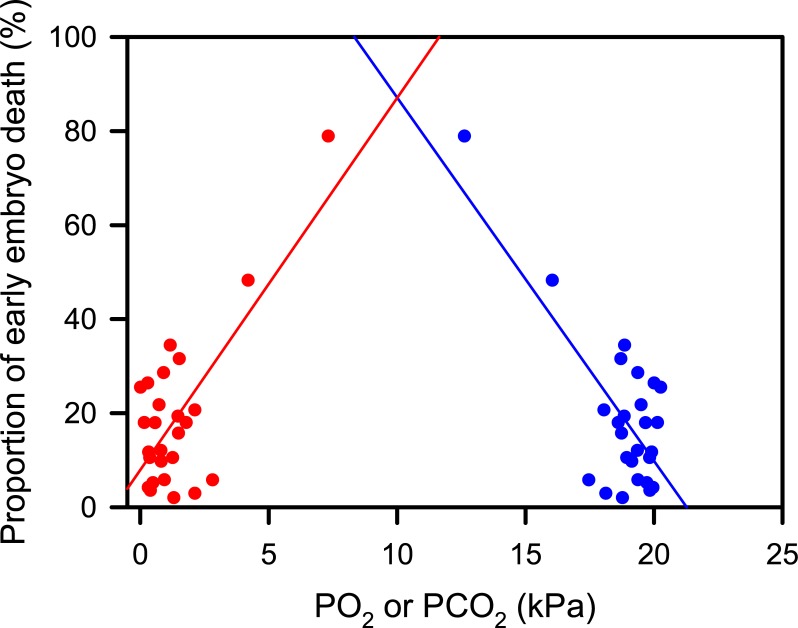
Relationship between oxygen partial pressure (PO_2_) and carbon dioxide partial pressure (PCO_2_) during the first 6 days of incubation and proportion of early embryo death in nests set up in December 2016 and February 2017 (n = 26). Blue circles and regression line represent PO_2_, red circles and regression line represent PCO_2_. Although the statistical analyses reported in the text were performed on arcsine transformed percentage data, for clarity, the data is represented as the original untransformed percent data, and the plotted regression lines were calculated using this raw percentage data.

The proportion of embryos that died within the first week of incubation was negatively correlated (statistical test performed on Arcsine transformed proportion data r = -0.76, t = -5.73, P < 0.001, n = 26) with mean PO_2_ experienced during the first 6 days of incubation and positively correlated with PCO_2_ (statistical test performed on Arcsine transformed proportion data r = 0.76, t = 5.65, P < 0.001, n = 26) (Fig 4). However, it should be note that both these correlation analyses are strongly influenced by the two nests that experienced the more extreme respiratory gas tensions, and more data from nests that experienced similar extreme gas tensions are needed to confirm this correlation.

### Nest temperatures

**Fig 5:**
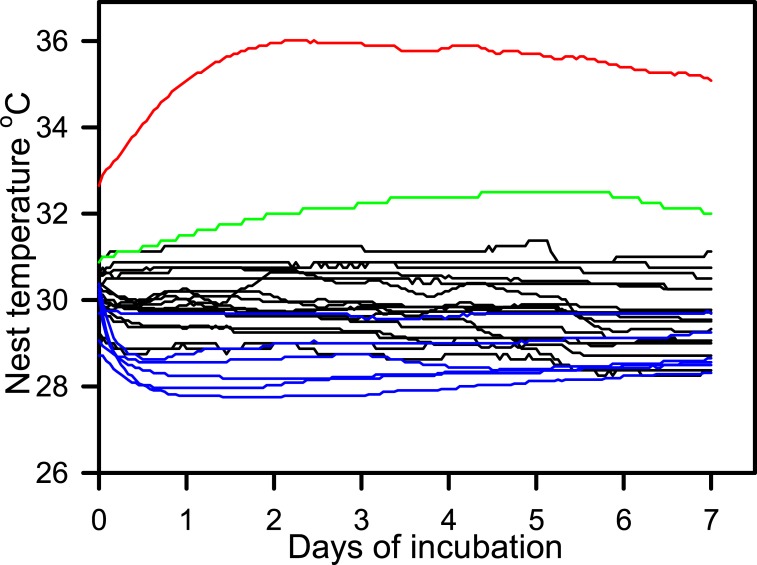
Nest temperatures during the first 6 days of incubation. Blue line represents nests set up in December. Black lines represent nests set up in February. Green line represents nest N31 set up in February. Red line represents nest N48 set up in February.

**Fig 6:**
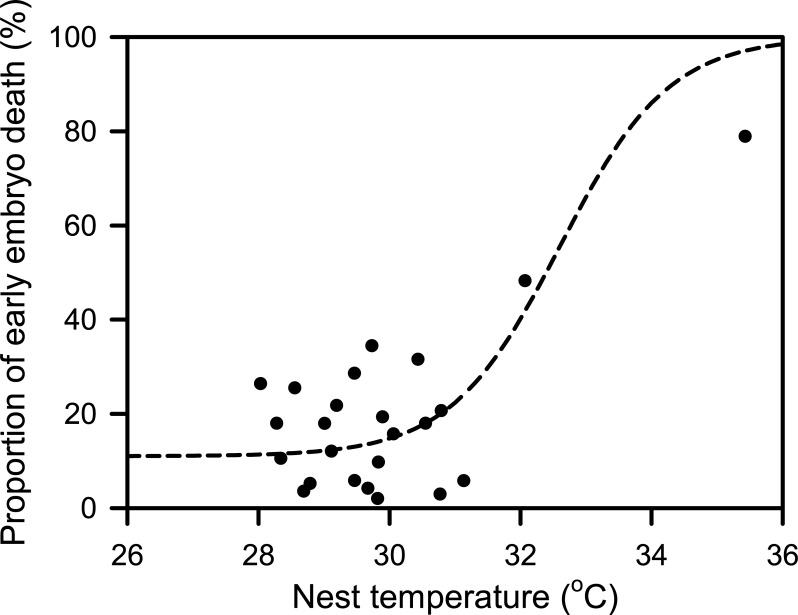
Relationship between mean nest temperature during the first 7 days of incubation and proportion of early embryo death in nests set up in December 2016 and February 2017 (n = 24). Dashed line represents the generalized relationship between incubation temperature and proportion of embryo death of all sea turtle species as defined by a logistic function [28].

Temperature data loggers were recovered from 6 nests that were set up in December 2016, and from 18 nests set up in February 2017. Within any particular nest, nest temperature was relatively constant during the first seven days of incubation, and with the exception of nests NG31 and NG48 were between 28°C and 31°C (Fig 5). Mean nest temperature over the first week of incubation during December (28.6 ± 0.2°C, n = 6) was cooler (F_1,20_ = 13.03, P = 0.002) than during February (29.9 ± 0.2°C, n = 16, excluding nests NG31 and NG48). Temperatures within nests NG31 and NG48 steadily increased for two days after nest construction before stabilizing (Fig 5). The proportion of early stage embryo death varied with incubation temperature in a way similar to that which has been reported for sea turtle embryos in general, moderately low mortality at temperatures below 31°C, and a large increase in mortality at temperatures above 33°C (Fig 6).

## Discussion

### Sand respiratory gas samples

When nests at Raine Island that have experienced EEDS are excavated at the end of incubation, most of the eggs were in a state of decay and infected with fungus and bacteria [2], but it is not known if these infections caused embryos to die, or have occurred secondarily after the embryos have died. High nest mortality of sea turtle embryos has been associated with high microbial loads within nests of olive ridley turtle arribada nesting aggregations at Ostional beach, Costa Rica [9,10]. The high microbial load in the sand at these beaches are associated with high concentrations of organic matter in the sand due to hatched eggshells and undeveloped eggs and dead embryos that accumulate in the sand [10,11,12]. However, at Ostional beach, it is unclear if the microbes themselves cause death, or if the high temperatures and low oxygen and high carbon dioxide conditions caused by microbial respiration is the cause of embryo death [10]. Decreasing the organic matter of sand (which decreases the microbial load of sand) can be achieved by sifting sand or by natural re-working of sand by storm and tidal surges and results in an increased hatching success [10], and presumably it is through these processes that the sand does not continue to increase its organic to very high levels that would result in very low hatching success.

In our Raine Island study, all of the respiratory gas samples taken from sand at nest depth within the transects had oxygen partial pressures slightly below, and carbon dioxide partial pressures slightly above atmospheric air indicating a low level of microbial respiration in the sand. Although we do not know of any studies that have experimentally examined the effect of depressed PO_2_ and elevated PCO_2_ on newly laid sea turtle eggs, we are confident that the deviations from atmospheric conditions we observed were not extreme enough to adversely influence embryonic development. We base this conclusion on studies that have reported field nest measurements of respiratory gases and also reported high hatching success from these nests [12,13,14,15,16,17,18,19,20,21]. This indicates that, at least for the 2016–2017 nesting season, the amount of organic matter residing in beach sand was not great enough to fuel a microbial respiratory load that could cause a significant depression in oxygen and elevation of carbon dioxide across the beach at green turtle nest level. Therefore, we reject the hypothesis that high nesting sand microbial load that causes unfavourable respiratory gas concentrations as the cause of EEDS for the 2016–2017 Raine Island nesting season. This conclusion is also supported by the fact that the vast majority of the nests we monitored (24 out of 26) did not experience early embryo death greater than 35%.

Interestingly, the sand PO_2_ during the 3 Feb and 6 Apr sampling trips were lower than the 2 Dec sampling trip. A possible explanation for this observation is that because the 2 Dec sampling occurred early in the nesting season there was relatively little organic material in the sand, but the organic content had increased by the time of the second two sampling dates. This resulted in a higher microbial load within the sand with its associated increase in oxygen demand. This hypothesis is supported by the findings that microbial density increases with nest density [7] and that differences between the ‘clean’ and ‘in situ’ sand organic content are correlated with the lower PO_2_ and higher PCO_2_ in olive ridley turtle nests constructed in ‘clean’ and ‘in situ’ sand during the first half of incubation [12].

### Nest respiratory gases and temperatures during the first 6 days of incubation

The slope of the line relating PO_2_ to PCO_2_ of nests indicate that gas exchange between the nest and atmospheric air occurs chiefly by diffusion (Fig 3). This is consistent with the empirical measurements and theoretical modelling of respiratory gas exchange in sea turtles nests [13].

For the majority of our monitored nests, PO_2_ and PCO_2_ was similar to sand values, and similar to atmospheric air as is usual for sea turtle nests early in incubation [12,13,14,15,16,17,18,19,20,21]. However, in two of our nests NG31 and NG48, PO_2_ and PCO_2_ were conspicuously different to other nests. Further investigation indicated that the reason for this difference was almost certainly their close proximity to sea turtle nests that were close to hatching as evidenced by epicentres of depressed PO_2_ and elevated PCO_2_. This close proximity to mature nests could also explain why these nests also experienced conspicuously higher nest temperatures than the other monitored nests because the combined metabolic heat production of mature embryos heats up the sand in the immediate vicinity of the mature nest. Laying nests close to a mature nest was not a problem early in the nesting season (November-early December) because there was not a high density of nests already incubating when females came ashore to nest. However, by mid-December and onward, nest density was much higher and the laying of nests adjacent to maturing nests became a potential problem. To obtain an estimate of how many nests might be affected by this phenomenon during the first week of February, we used the proportion of our sampled nests that were affected (2/19) and the estimated number of turtles ashore at night attempting to nest (~5,500 [2]), and the measured nesting success (0.39 [2]), to estimate the number of nests successfully constructed per night (2,145), and the number of these nest likely to be laid adjacent to late maturing nests (226). Hence, a not inconsiderable number of nests are at risk of EEDS because of the high density of nests laid on the island at this time.

Exposure to lethal concentrations of respiratory gases and high incubation temperature, especially early in incubation have been suggest as possible causes of sea turtle embryo death [10,12,18,19]. When first laid, sea turtle eggs are typically exposed to oxygen (PO_2_) and carbon dioxide (PCO_2_) partial pressures close to atmospheric air (oxygen ~21 kPa, carbon dioxide ~0 kPa). However, during the rapid embryo growth phase of embryonic development when embryos are much larger, their combined oxygen consumption and carbon dioxide production causes the within nest oxygen and carbon dioxide partial pressures to deviate appreciably from atmospheric conditions [13]. As a consequence, during the final stages of incubation, PO_2_ frequently drops below 15 kPa and PCO_2_ frequently increases above 5 kPa [12,13,14,15]. Clearly, late stage sea turtle embryos are tolerant of hypoxic and hypercapnic conditions, but early stage embryos may be less tolerant to these more extreme conditions. We now know that low oxygen availability while eggs are still in utero is what keeps green turtle embryos in a state of paused development before they are laid, and that exposure to high oxygen levels causes embryos to break developmental arrest once eggs are laid [22]. However, once exposed to high oxygen for between 12–16 h, if developing embryos are then exposed to PO_2_ < 1 kPa they die [22]. Our very preliminary data from nests NG31 and NG48 suggest that the decreased PO_2_ and increased PCO_2_ may have resulted in an increase in early embryo mortality in these nests. In the 48 hours following nest construction nest temperatures and PCO_2_ rose while PO_2_ fell in nests NG31 and NG48 which is what is expected as the newly constructed nests come under the influence of the adjacent mature nests. It would also appear that the adjacent mature nests hatched and the hatchlings began escaping their nests after day 5 in the nest near NG31 and after day 4 in the nest near NG48 because the nest temperature and PCO_2_ decreased and the PCO_2_ increased in these nests at those times. These observations support the hypothesis that EEDS is associated with deviations of respiratory gas from atmospheric conditions and or/high nest temperatures.

Elevated nest temperature combined with depressed PO_2_ during the first half of incubation was correlated with increased mortality of oliver ridley turtle embryos, although the cause of increased temperature and depressed PO_2_ was high sand microbial load rather than newly laid nests being located next to mature nests in this case [10]. In sea turtle nests, increased temperature, increased PCO_2_ and decreased PO_2_ occur in unison as incubation proceeds because the embryos become larger in mass which increases their oxygen consumption, carbon dioxide production and heat production. Occasionally an increase in nest temperature may become decoupled form changes in respiratory gases for short periods of time during and immediately after heavy rainfall events that cause a decrease in sand and thus nest temperature. If as appears likely, EEDS is caused by mature nest respiratory gas and temperature conditions being experienced in newly constructed nests, it is of interest to determine if it is the combination of these conditions that cause embryo death, or if any one condition is sufficient to cause death. Sea turtle embryos are tolerant of temperatures between 34°C and 36°C for days at a time by the end of incubation [23,24,25,26] but early stage embryos appear not to tolerate temperatures above 34°C [26, 27, 28, 29,30]. In the same way, early stage sea turtle embryos may be less tolerant of decreased oxygen and elevated carbon dioxide conditions than mature embryos. So high temperatures, or altered respiratory gas concentrations, or a combination of the two may kill early stage sea turtle embryos in natural nests laid adjacent to a mature nest, but controlled laboratory experiments will be needed to discover if depressed oxygen, elevated carbon dioxide or high temperature are sufficient to cause death by themselves.

One of the limitations of the results presented in this paper is that the correlations between PO_2_ and EEDS, PCO_2_ and EEDS, and temperature and EEDS rely heavily on just two data points. In this light, the results we put forward in this paper should be treated with a degree of caution. In order to confirm our preliminary findings, further field experiments are needed to record data at extreme and intermediate values for PO_2_, PCO_2_ and nest temperature. Only then will our understanding of the effects of PO_2_, PCO_2_ and nest temperature on EEDS be more complete. We encourage more research to build on the preliminary results presented in this paper.

## Supporting information

S1 Fig Representation of Raine Island indicating location of experimental green turtle nests. Black pins indicate nests set up in December 2016 that were not disturbed by subsequent nesting turtles. Red pins indicate nests set up in February 2017 that did not experience high levels of early embryo death. Green pins indicate nests set up in February 2017 that did experience high levels of early embryo death. Modified from Goggle Earth Pro (2018) Google Inc. Available at: https://www.google.com.au/maps/place/Raine+Island/@-11.5906114,144.0332035,17z/data=!3m1!4b1!4m5!3m4!1s0x69a5d4497bb2144b:0x1d8800dbd8c282ce!8m2!3d-11.5908498!4d144.0350979. Verified 21 March 2018.
(TIF)

S1 File Raw data. This is a Microsoft excel file containing the raw data that was used in this report.
(XLSX)

S2 File Reference [2]. This is a PDF file of Raine Island Recovery Project: 2016–17 Season technical report to the Raine Island Scientific Advisory Committee and Raine Island Reference Group. Brisbane: Department of National Parks, Sport and Racing, Queensland Government. 2017 cited as [2] in the article text.
(PDF)
