# Supplementary figures and images for: A preliminary investigation into the early embryo death syndrome (EEDS) at the world’s largest green turtle rookery
Source: PLoS One. 2018 Apr 25;13(4):e0195462. doi: 10.1371/journal.pone.0195462 (PMC5918617; doi:10.1371/journal.pone.0195462)

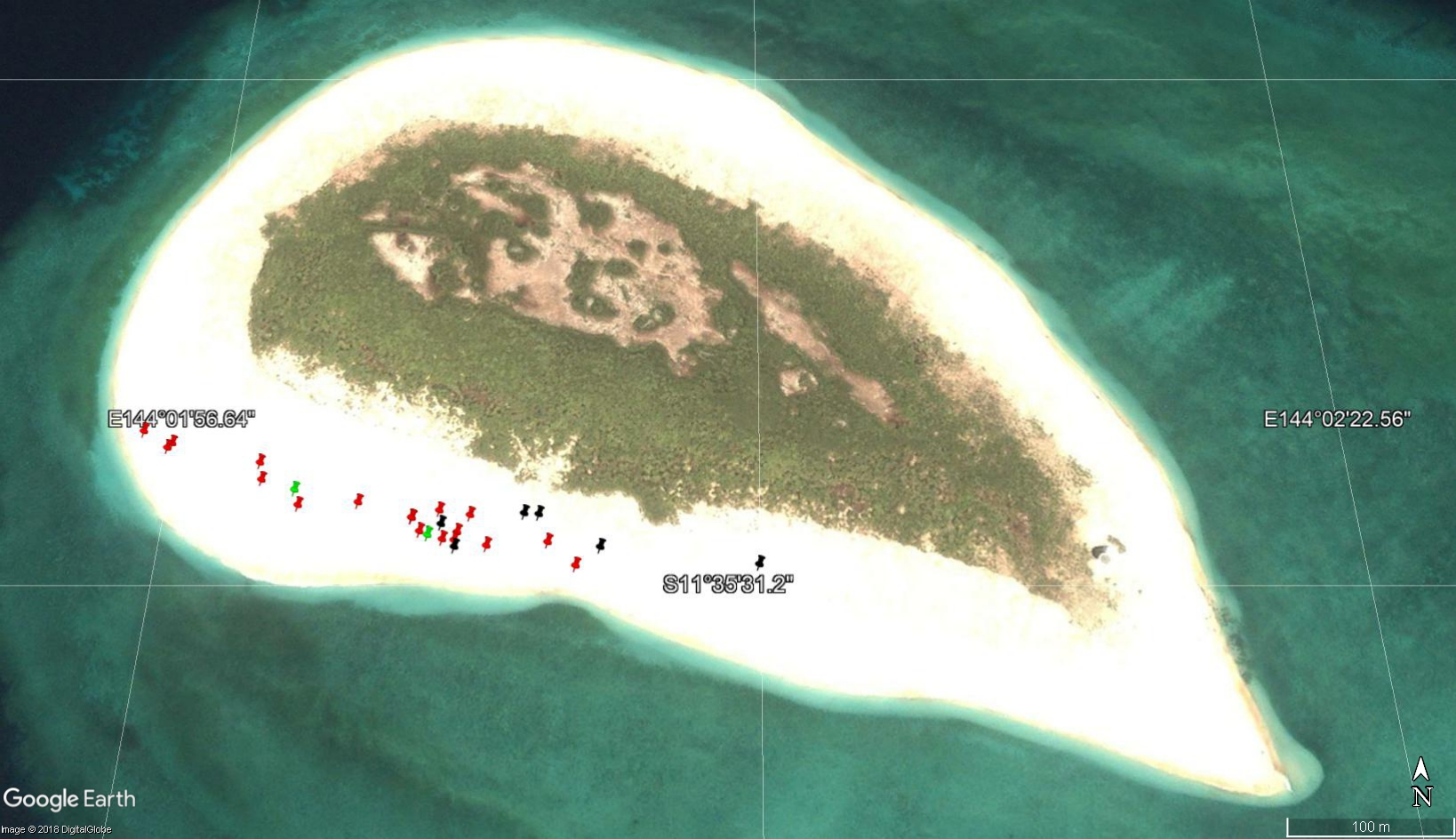

Supplement: S1 Fig — Black pins indicate nests set up in December 2016 that were not disturbed by subsequent nesting turtles. Red pins indicate nests set up in February 2017 that did not experience high levels of early embryo death. Green pins indicate nests set up in February 2017 that did experience high levels of early embryo death. Modified from Goggle Earth Pro (2018) Google Inc. Available at: https://www.google.com.au/maps/place/Raine+Island/@-11.5906114,144.0332035,17z/data=!3m1!4b1!4m5!3m4!1s0x69a5d4497bb2144b:0x1d8800dbd8c282ce!8m2!3d-11.5908498!4d144.0350979. Verified 21 March 2018. (TIF) [file pone.0195462.s001.tif]
