# Supplementary material for: A preliminary investigation into the early embryo death syndrome (EEDS) at the world’s largest green turtle rookery
Source: PLoS One. 2018 Apr 25;13(4):e0195462. doi: 10.1371/journal.pone.0195462 (PMC5918617; doi:10.1371/journal.pone.0195462)
Supplement: S2 File — This is a PDF file of Raine Island Recovery Project: 2016–17 Season technical report to the Raine Island Scientific Advisory Committee and Raine Island Reference Group. Brisbane: Department of National Parks, Sport and Racing, Queensland Government. 2017 cited as [2] in the article text. (PDF) [file pone.0195462.s003.pdf]

Raine Island Recovery Project

2016-17 Season Technical Report

DRAFT

Prepared by: Queensland Parks and Wildlife Service Marine Parks, Department of National Parks, Sport and Racing

© State of Queensland, 2017.

The Queensland Government supports and encourages the dissemination and exchange of its information. The copyright in this publication is licensed under a Creative Commons Attribution 3.0 Australia (CC BY) licence.

Under this licence you are free, without having to seek our permission, to use this publication in accordance with the licence terms.

You must keep intact the copyright notice and attribute the State of Queensland as the source of the publication.

For more information on this licence, visit http://creativecommons.org/licenses/by/3.0/au/deed.en

Disclaimer

This document has been prepared with all due diligence and care, based on the best available information at the time of publication. The department holds no responsibility for any errors or omissions within this document. Any decisions made by other parties based on this document are solely the responsibility of those parties.

If you need to access this document in a language other than English, please call the Translating and Interpreting Service (TIS National) on 131 450 and ask them to telephone Library Services on +61 7 3170 5470.

This publication can be made available in an alternative format (e.g. large print or audiotape) on request for people with vision impairment; phone +61 7 3170 5470 or email <library@nprsr.qld.gov.au>.

Citation

[1 to 6 authors]

Dunstan AJ. And Robertson K. 2017. Raine Island Recovery Project: 2016-17 Season technical report to the Raine Island Scientific Advisory Committee and Raine Island Reference Group. Brisbane: Department of National Parks, Sport and Racing, Queensland Government.

April 2017

Contents

Executive summary 1
Introduction 3
2016-17 Raine Island Recovery Project fieldwork…………………..4
2016-17 Research methods and results..5
Discussion..59
Acknowledgements..60
References..61
APPENDIX..62

Project Objective

To restore and maintain Raine Island as a viable island ecosystem which facilitates green turtle breeding, and seabird nesting and feeding, to support sustainable populations of those species.

Executive Summary

The Raine Island Recovery Project is an innovative program to improve the reproductive success and reduce adult female mortality of green turtles at Raine Island. The decision has been made to act rather than 'sit on our hands' and monitor a potentially serious ecological problem, in particular a major decline in the nGBR green turtle population. Intervention works at arguably the most important site in the GBRWHA are not taken lightly and risk assessment and mitigation of impacts underpin decision-making and actions.

Research at Raine Island suggests that tidal inundation and turtle nesting density are the main drivers for hatching failure. Sand re-profiling of the nesting beach to raise nest levels above the peak tidal water table was identified at the Raine Island Reference Group meeting in 2014 as the best management approach to reduce hatching failure due to inundation. Sand replenishment of the low lying sections of the nesting area was also predicted to increase the area utilised by nesting turtles, reduce nesting disturbance, increase nesting success and increase hatching success and hatchling production.

A 100m x 150m section of the nesting beach was re-profiled in September 2014, as described previously (Dunstan 2015). The results of the beach re-profiling showed increased nesting and hatching success and hatchling production, providing encouragement to expand the scope of beach re-profiling.

Results of adaptive management actions already undertaken demonstrate their effectiveness.

Increasing reproductive output. Nesting beach re-profiling has:

- Created 4 times more viable nesting area in the re-profiled sector than previously
- Increased hatching success
- Increased nesting efficiency
- Decreased nesting density (turtles spread out to nest throughout the entire area), resulting in a reduction in clutch destruction and nesting turtle disturbance
- Increased hatchling production

Significant egg death in clutches above inundation level indicates that factors other than clutch drowning are contributing to hatching failure. The negative correlation between nest density and hatching success provides support for the hypothesis that nest density dependant factors are causing high embryonic mortality. The factors causing embryo mortality relating to nest density have not yet been identified but are likely to be respiratory gas levels, sand composition, high microbial load, toxins or pathogens within nests.

Nesting failure is also a major concern for turtle reproduction at Raine Island. Dry and fine sand conditions and inter-nesting disturbance amongst turtles are considered the main causes for nesting failure of adult females and may result in reduced reproductive output due to increased energy expenditure on re-nesting efforts which can cause follicle resorption (Limpus et al, 2003) and may reduce health and hatching potential of eggs retained for multiple days.

Mature female turtle mortality is another area of impact on the population. The causes of adult mortality on Raine Island are heat exhaustion (65%), cliff falls (30%) and entrapment in cliff areas and beachrock (5%). Installing fencing from 2011 onwards to high-risk cliff areas has resulted in a major reduction in cliff-fall mortality while turtle rescues have greatly reduced mortality from heat exhaustion and other factors.

Reducing nesting mature female turtle mortality:

- Cliff-top fencing installation has reduced overturned turtle mortality by more than 70%, saving over 400 turtles. More fencing installation is planned, including the need for adaptation of fencing methods to counter rock falls as nesting turtles undermine the cliffs.

- Machinery assisted turtle rescues now enable staff to rescue all turtles during their presence at Raine Island equating to hundreds of mature female turtles during peak seasons.

The focus of the 2016-17 nesting season was on addressing current knowledge gaps, consolidating the monitoring regime and associated method standards.
The focus of the 2016-17 season was to compare turtle nesting and hatching success between the re-profiled sector and control sectors, to conduct preliminary observations on nest environment respiratory gas and temperature effects on incubation success and to investigate, quantify and plan logistics for future sand movement

works in late 2016-17. Measurement of nesting success, hatchling production, hatching success and turtle mortality was continued as in previous years. No major intervention work (sand movement) was conducted this season. Moulter Cay was also the subject of parallel investigation of nesting and hatching success and hatchling production as well as inundation and geomorphology surveys to assess whether similar issues to those affecting Raine Island are affecting Moulter Cay.

Key outcomes and findings of the 2016-17 season:

- This was a low-medium nesting density season with 11,960 mature female green turtles aggregated around Raine Island during early December 2016.
- There were 168 dead adult female turtles recorded (not including rescued turtles which would otherwise have died) on the nesting beach for the season up until April 10, 2017
- 161 turtles were rescued at Raine Island during field trips in November, December and February with no rescues required in April.
- Re-profiled sector has continued to retain its profile with minimal change.
- Inundation is consistent in height around the island and is concentrated in the swale and back swale areas except for the western end of the island where berm and back berm height is significantly lower. This corresponds with a lower level of emergence success recorded in these locations (NW end: 21.3%, other areas: 51.2%).
- Inundation closely follows the tidal cycle with height increase throughout linked to major rainfall events and wind driven wave and surge events, which only affect the berm area. Nests throughout the inundated areas have low hatching success.
- Nesting success was similar in late January (29.8%) to early November (27.2%), but much higher than in early December (7.6%), correlating with low rainfall and dry sand conditions leading up to December and consistent rain and moist nesting beach sand in late October and during January and February.
- Hatchling production and reproductive success was high (for Raine Island) in both February and April.
- Hatchling counts per night per 50m survey sector in February were similar for control (1325 and 1461) and re-profiled areas (1416). In April hatchling production was much higher in the re-profiled area (1972) than in control areas (1348, 998 and 775)
- Reproductive success in the re-profiled sector compared to the control sectors was similar in the Dec – Feb incubation period (57%: 58%). In the Feb – April period the re-profiled sector reproductive success (69%) was much higher than in the control sectors (33%).
- Hatching success in the re-profiled sector compared to the control sectors was similar in the Dec – Feb incubation period (62%: 59.4%). In the Feb – April period the re-profiled sector hatching success (62.5%) was higher than in the control sectors (46.2%).
- Comparison of nests exhibiting emergence failure and sections of the beach experiencing tidal inundation showed these are highly correlated.
- Nests laid in the re-profiled sector were above inundation level at all times.
- Death during the first phase of embryonic development is the major contribution to low hatching success.
- In December 2016 thirteen clutches were protected by timber stakes, dGPS marked, had temperature loggers placed mid-clutch and O_2 and CO_2 level readings taken during the first 8 days of incubation. Nests were then excavated and examined for emergence success 60 days later. A similar procedure was undertaken for 19 clutches laid in February 2017. Two clutches exhibited very low O_2 and correspondingly high CO_2 levels early in development and had extremely low hatching success in contrast to the remainder of the clutches. Nest temperature levels indicate only female hatchlings would have been produced from all clutches.
- Moulter Cay exhibits similar inundation and has a shallow sub-surface swale rock layer similar to Raine Island. Preliminary results indicated that nesting and hatching success and hatchling production at Moulter Cay are similar to that recorded at Raine Island. However, it is important to recognise that limited survey time, and hence data, was available during February for comparison of reproductive outcomes.
- Seabird counts and reproductive status were again undertaken by both ground observer and drone methods in December and April. Analysis is underway to compare and evaluate the relative merits of each method.

- Red-tailed tropicbird nesting location and breeding status counts were conducted on all trips. This continues to demonstrate the availability of many more nesting locations than number of birds. Artificial nesting boxes were installed but no red-tailed tropicbirds made use of these.

Introduction

Green turtles, *Chelonia mydas*, are listed as vulnerable under both the Environment Protection and Biodiversity Conservation Act (1999) and the Nature Conservation Act (1992). The Raine Island* Recovery Project aims to increase reproductive success and reduce adult mortality for green turtles at Raine Island. Queensland Department of National Parks, Sport and Racing leads the project in partnership with EHP Threatened Species Unit, the Great Barrier Reef Marine Park Authority and Wuthathi and Meriam Nation Traditional Owners with considerable support from collaborating organisations.

*Raine Island as used in this project refers to Raine Island National Park (Scientific), which includes Raine Island, Moulter Cay and McLennan Cay. An estimated 90% of nGBR green turtles nest at Raine Island and Moulter Cay.

Concerns about low reproductive success of green turtles at Raine Island have been reported since 1996 (Limpus et al 2003 and annual internal Queensland Government technical reports). Low reproductive success has been verified by research over the last six breeding seasons (2011-2012 to 2016-17). Nesting success (the percentage of turtles attempting to nest that actually lay successfully) in a night varies from 20% to 61% compared to approximately 85% in other Great Barrier Reef (GBR) coral cay green turtle rookeries (Limpus 2008).

Hatchling production success (the percentage of hatchlings emerging successfully from the number of eggs laid) is 10-50% compared to 75-85% in other GBR coral cay green turtle rookeries (Limpus 2008). The repetitive failure to lay eggs when the female comes ashore may lead to a reduction in the number of clutches that the female will lay for the season. This has not been quantified for the Raine Island nesting population but is expected to represent a significant reduction in hatchling production. Clutch and egg mortality is high. There is also a high mortality of nesting females, with mortality as high as 2000 individuals in a single high-density nesting season, however this has probably been occurring for more than 150 years on Raine Island (Limpus et al 2003). This level of mortality is still small (around 3%) compared to the size of the annual nesting population.

The very poor hatchling production since at least the 1996-1997 breeding season will result in a drastic reduction of breeding adults at Raine Island in the in the next generation. A major decline within the Northern GBR (nGBR) green turtle stock is predicted within the next 20-30 years unless immediate actions to mitigate these problems are undertaken.

The Raine Island Recovery Project also recognises the importance of understanding and sustainably managing the entire Raine Island ecosystem. Parallel research is being undertaken through a variety of partnerships into seabird nesting, climate change, island geomorphology and apex predators.

2016-17 Raine Island Recovery Project Fieldwork

The 2016-17 season work program is detailed in Table. 1.

Table 1. *2016-17 Field trips*

Date	Days on site	Personnel	Activities	Vessel	Funding
July 7-13	6 - Raine	6 x QPWS<br>2 x TO<br>1 x DNRM<br>1 x tower restoration TRM<br>1 x volunteer	Fencing<br>Tower survey<br>Network download<br>Topographic survey	Reef Ranger	FMP
Nov 1 - 13	10 - Raine	10 x QPWS<br>2 x TO's<br>2 x JCU<br>1 x UQ<br>1 x volunteer	Turtles – nesting success, mortality, nest marking, satellite tagging, tagging, 500 count, total census, rescue, necropsy, nest gas monitoring<br>Topographic survey, Network download, Seabird survey + RTTB	Reef Ranger	FMP
Nov 28 – Dec 7	8 - Raine	9 x QPWS<br>1 x GBRMPA<br>1 x DNRM<br>2 x TO's<br>2 x UQ<br>1 x Biopixel	Turtles – nesting success, mortality, nest marking, tagging, 500 count, total census, rescue, necropsy, nest gas monitoring<br>Topographic survey, Network download, Seabird survey + RTTB, Drone surveys	Reef Ranger	FMP
Dec 8 - 9	1 - Raine	7 x QPWS<br>2 x TO elders<br>1 x GBRMPA<br>1 x Threatened Species Commissioner<br>1 x GBRF<br>3 x BHP Billiton<br>1 x JCU	Delegates trip<br>Presentation of RIRP research and management activities on-site<br>Turtles –tagging, rescue, necropsy	Reef Ranger	RIRP
Dec 9 - 16	2 – Raine<br>4 - Moulter	10 x QPWS<br>2 x TO's<br>2 x JCU<br>1 x DEE<br>1 x Activ8me	Turtles – nesting success, mortality, nest marking, tagging, 500 count, rescue<br>Topographic survey, Network download, Seabird survey + RTTB, satellite install	Reef Ranger	FMP
Jan 27 - Feb 9	9 – Raine<br>Cut short by 2 days due to weather	9 x QPWS<br>1 x Qld Museum<br>1 x DNRM<br>1 x Volunteer<br>2 x TO's<br>1 x UQ	Turtles: hatching survey, nest digging and marking, nesting success, tally count, 500 count, rescue<br>Topographic survey, Network download	Reef Ranger	FMP
Feb 9 - 10	1 – Raine	6 x QPWS<br>1 x Minister Miles<br>1 x Minister assistant<br>2 x TO elders	Delegates trip<br>Presentation of RIRP research and management activities on-site<br>Turtles –rescue	Reef Ranger	RIRP

		1 x GBRF<br>3 x BHP Billiton<br>1 x Courier Mail<br>2 x ABC news			
Feb 10 - 15	2 – Raine<br>2 - Moulter	9 x QPWS<br>1 x Qld Museum<br>1 x DNRM<br>1 x JCU<br>2 x TO's<br>1 x UQ	Turtles: hatching survey, nest digging and marking, nesting success, tally count, 500 count, rescue<br>Topographic survey, Network download, nest gas monitoring	Reef Ranger	FMP
April 3 - 12	7 - Raine	8 x QPWS<br>1 x Volunteer<br>2 x JCU<br>2 x TO's<br>1 x Biopixel<br>1 x UQ<br>1 x NOAA	Turtles: Hatchling survey, nest gas monitoring, rescue, mortality<br>Drone surveys, Nest digging, Seabird survey + RTTB, Remote sensing download, Inundation mapping	Reef Ranger	RIRP

2016-17 Research methods and results

1. NESTING TURTLE POPULATION / PRESENCE

1a. Nesting beach tally count

The 2016-17 season was a low-mediem level season for nesting turtle numbers (Table 2).

Methods

An instantaneous count of all turtles was conducted by four persons walking line abreast across the beach, for one circuit of the island between 2030-2230hr (a tally count usually took 40-60min. to complete). Counts commenced at least one hour after turtles had swimming access to the nesting beach across the reef flat after dark. During the summer nesting season the tides are such that on most of the nights, the majority of the turtles for the night will have beached within a few hours of darkness. A green turtle rarely lays and returns to the water in less than 3hr on the beach.

Results

The mean tally count for the early November nesting period was 1090 ± 487.6 S.E.

The mean tally count for the late November/December nesting period was 5495 ± 277.6 S.E.

The single tally count for December at Moulter Cay was 1384 (Table 2)

Tally counts were not conducted during the Jan / Feb trip however these have been estimated from data recorded during nesting success surveys.

Table 2. *Tally counts conducted during 2016-17 season*

Date	Location	Total

13/12/16	Moulter Cay	1384
2/11/16	Raine Island	755
4/11/16		862
6/11/16		834
8/11/16		1149
10/11/16		1851
**Mean early Nov tally**		**1090 ± 487.6 S.E**
29/11/16		5565
3/12/16		5699
4/12/16		4709
9/12/16		6005
**Mean Nov/Dec tally**		**5495 ± 277.6 S.E**

1b. Petersen estimate of the size of a nesting population

The 2016-17 was a low-medium level season for breeding female turtles at Raine Island. The comparison of techniques using historical vessel based observer counts, GoPro underwater vessel mounted camera counts and drone based video counts showed very promising results for use of both GoPro and drone methods. Drone counts were more efficient (approximately ¼ of the field time) and resulted in higher total turtles recorded with a similar precision to vessel based observer counts (Tables 3 a, b & c).

Methods

If a sample of the turtles ashore on Raine Island at night are marked such that they can be recognised in the water over the following days, and if the marked turtles are uniformly distributed among the unmarked turtles in the waters surrounding Raine Island, then mark-recapture data can be analysed using the Petersen estimate with Bailey's correction to provide an estimate of the number of adult green turtles in the waters surrounding Raine Island at that time (after Limpus et al. 2003).

In a population where M animals are marked out of a total population of N animals and m marked animals are recaptured in a subsequent sampling of n animals:

$$N = M(n+1) / (m+1)$$

This estimate has a standard error of approximately:

$$SE = [M2(n+1)(n-m)]1/2 / (m+1)2(m+2)$$

The principal assumptions made in using this method are:

- Tagged turtles are uniformly distributed among the untagged turtles that form the Raine Island breeding assemblage on that day;
- There is a negligible rate of tag loss between tagging and recapture;

- The “tags” are easily seen on a swimming turtle;
- All the females aggregated on Raine Island Reef are there to breed i.e. there is an insignificant resident non-breeding female population on Raine Island Reef;
- There is equal probability of the tagged and untagged turtles moving outside the sampling area; and
- There is negligible mortality of the adult turtles during the sampling period.

Turtles were painted with white “APCO-SDS fast dry water-based road marking paint” (MSDS Infosafe No. 1WDKY) applied as longitudinal stripes by spray application then spread by 12 cm wide rollers to the midline of dry carapaces while the turtles were ashore for nesting at night. A turtle was selected for painting if the carapace was dry, the carapace did not have a thick coating of algae and the turtle was inland of the beach crest, well within the nesting habitat. When applied under these conditions, the paint could be expected to adhere well to the carapace surface for at least 96hr.

A search was made for marked turtles among the turtles in the waters surrounding the island on the morning and afternoon of the following two days. An outboard powered inflatable tender with three persons aboard, one recording, one driving and one counting, was driven across the reef flat at the upper part of the tidal cycle and along the outer edge of the reef in search of the painted turtles. By driving adjacent to the swimming turtle, it was possible to score it by species, size class, tail length and presence or absence of markings as above. Turtles sighted were recorded for species, sex and size class. Those sighted with long tails were scored as males, adult-sized short tailed turtles were scored as presumed adult females and those smaller than adult females (CCL<90cm for *C. mydas*; CCL<75cm for hawksbill turtles, *Eretmochelys imbricata*) were scored as immature. A count was made of the female turtles that could be scored positively for the presence/absence of the white paint mark.

During the 2016-17 season a painted turtle mark resight nesting population estimate was conducted twice, on Nov 4, 2016 (781 turtles painted) and on Dec 1, 2016 (2000 turtles painted). On the following three days in November and December vessel based sighting surveys were conducted to record painted vs unpainted adult female turtles (Fig. 1).

GoPro surveys were conducted from the survey tender simultaneously with all surface observer surveys (Tables 3a, b &c). Drone surveys were conducted immediately prior to GoPro and observer surveys in December.

Video footage was viewed by one observer using two tally counters to record each of painted and unpainted turtle numbers. Video was paused, slowed or sped up to improve counting efficiency and accuracy. Further review of data will be undertaken using three observers.

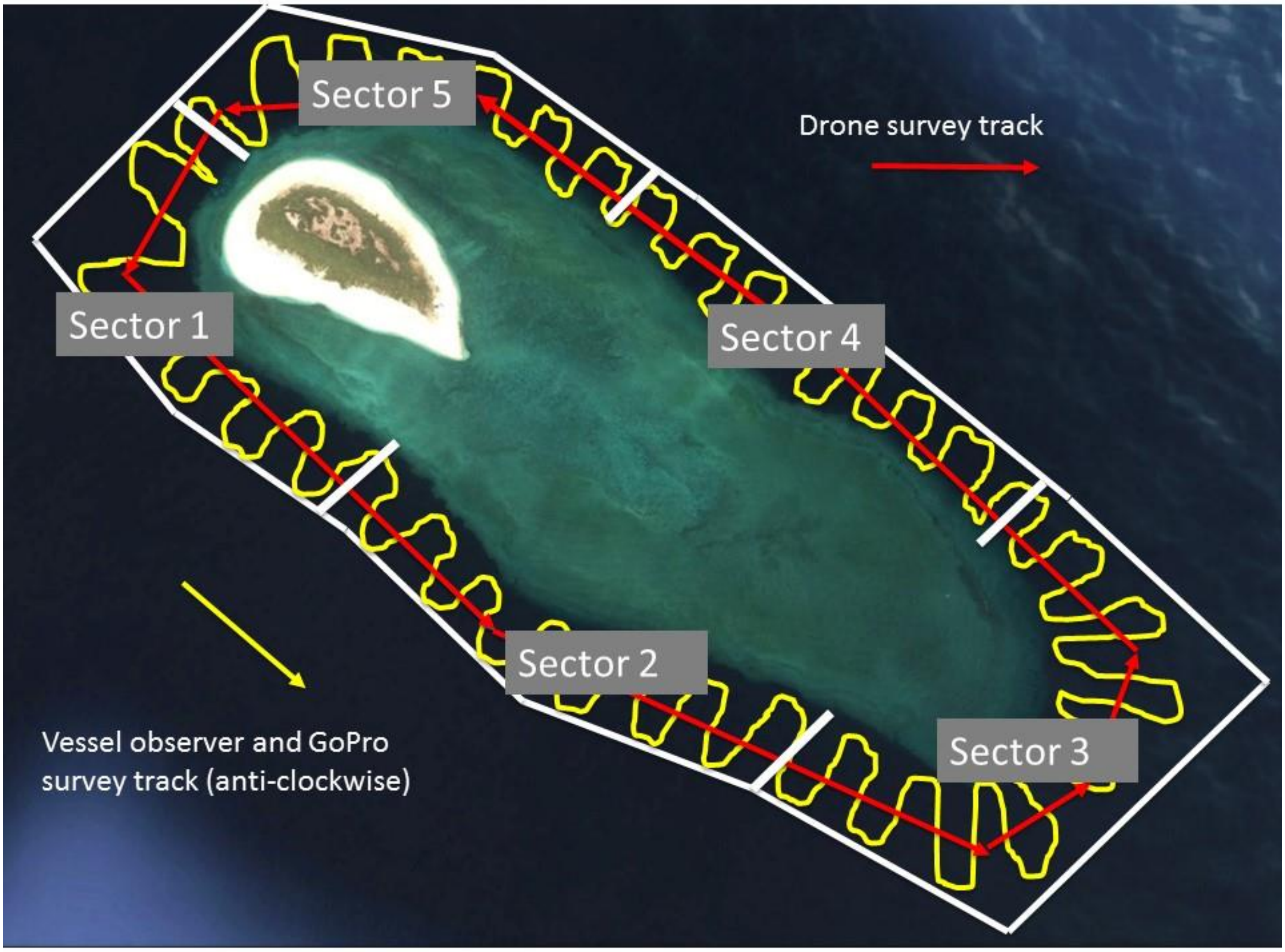

Figure 1. *Survey tracks for vessel based observer and GoPro surveys and Drone surveys of painted turtles*

Results

The estimated number of breeding female *C. mydas* aggregated at Raine was 8144 ± 1074 in early November 2016 (Table 3a) and 11960 ± 425 in early December 2016 (Table 3b) by vessel based sighting surveys.

The estimated number of breeding females in comparative drone surveys in early December 2016 was 12508 ± 567 (Table 3c).

GoPro footage is currently being analysed.

Table 3a. *November 5-7, 2016 painted turtle surface observer surveys*

Observer survey

Variable	Symbol	5/11/16 am	5/11/16 pm	6/11/16 am	6/11/16 pm	7/11/16 am	7/11/16 pm	Mean
Number of nesting turtles painted	M	781						
Total turtles counted in survey		496	847	547	712	703	664	**661**
Peterson estimate (total breeding females)	N	5183	8214	9871	7820	9637	12305	**8144**
Standard error	SE	± 506	± 824	± 1367	± 829	± 1164	± 1756	**± 1074**

Table 3b. *December 2-4, 2016 painted turtle surface observer surveys*

Observer survey

Variable	Symbol	2/12/16 am	2/12/16 pm	3/12/16 am	3/12/16 pm	4/12/16 pm	4/12/16 pm	Mean
Number of nesting turtles painted	M	2000						
Total turtles counted in survey		560	874	589	475	616	430	**590**
Peterson estimate (total breeding females)	N	10358	12593	12424	12326	12098	14463	**11960**
Standard error	SE	± 313	± 350	± 416	± 457	± 389	± 627	**± 425**

Table 3c. *December 3-4 painted turtle Drone surveys*

Drone survey

Variable	Symbol	3/12/16 am	3/12/16 pm	4/12/16 am	Mean
Number of nesting turtles painted	M	2000			
Total turtles counted in survey		1197	825	720	**914**
Peterson estimate (total breeding females)	N	**13621**	**11763**	**12141**	**12508**
Standard error	SE				**± 567**

1c. ‘500 counts’

Methods

A team of three persons, one tagging and measuring and the other recording, moved through the nesting turtles and examined as many turtles as possible in a single night.

- Every turtle examined was marked with paint ‘dob’ on carapace (paint colour was chosen to identify date of record).
- All turtles examined were counted
- Every turtle with an existing paint mark from a previous night was recorded.
- Turtles with special characteristics were recorded:
- Fresh shark attack (WS)
- Fibropapillomas (PG)
- Other unusual features

All turtle recaptures were retagged where tags were lost or poorly attached, existing tags and tag scars recorded and CCL measurements taken.

Results

A total of 4269 turtles were examined over five nights in early November, 2016 and two nights in early December, 2016 with the following records taken (Table 4)

Table 4: *Summary of 500 count data*

Date	2/11/16	4/11/16	6/11/16	8/11/16	10/11/16	3/12/16	4/12/16	Total
Total turtles examined	522	500	605	550	509	1014	569	**4269**
Total remigrants (individuals tagged in previous years)	29	16	29	30	12	68	22	**206**
Healed tag scars								
Within trip recaptures	2	9	60	57	29	39	36	**232**

1d. Turtle tagging

Methods

Tagging was carried out as described previously using standard titanium turtle flipper tags (Limpus, 1992).

Results

Tags applied – 1746 tags were applied at Raine Island this season. Tagging was highly efficient during 2016-17 with tags not needing to be pre-bent and application successful on the first attempt in nearly all instances.

2. MORTALITY AND RESCUE OF NESTING TURTLES

Methods

The number of dead and moribund turtles at each studied rookery was recorded on arrival. Carcasses were marked with a pink paint cross to eliminate double recording. Moribund turtles were rescued where possible, especially those that had fallen onto their backs. These rescued turtles were counted as mortalities for the purposes of the study. A turtle rescue and dead turtle census walk were undertaken each day between 0930-1100hrs when the schedule permitted.

The GPS location, CCL measurement, cause of death or impending death and state of the carcass relating to time since death were recorded for dead or rescued turtles where possible.

Necropsies were performed as described previously (Limpus 2003)

Results

2a. Nesting turtle mortality

2016-17 was a low-medium density nesting season with low mortality of nesting female turtles. Most nesting effort occurred on the berm area of the beach with few turtles traversing the beach to the cliff areas in November and December but with greater numbers in the cliff area in Jan/Feb.

A total of 169 dead turtles at Raine Island and 65 at Moulter Cay were recorded (Table 5).

161 turtles that would have died if the team had not been at Raine Island and 10 turtles at Moulter Cay were rescued during fieldwork periods and released to the sea. These rescued turtles are recorded as if they would have died in the appropriate categories for cause of death (Table 7).

Table 5: *Dead turtle count at Raine Island and Moulter Cay for 2016-17 season*

** Note: rescues are included in the appropriate cause of death column as well to be consistent with previous years which treated rescued turtles as mortalities in the final data.*

Date	Heat	Cliff falls	Cliff entrapment	Beachrock	TOTAL
**RAINE**					
Nov 2-11, 2016	8	1	3	0	12
Nov 30 - Dec 11, 2016	72	4	6	2	84
Jan 29 - Feb 9, 2017	18	33	3	0	54
April 4 - 9, 2017	5	13	1	0	19

**TOTAL**	**103**	**51**	**13**	**2**	**168**
**MOULTER**					
Dec 12-14, 2016	27	0	0	23	50
Feb 12-14, 2017	8	6	1	0	15
**TOTAL**	**35**	**6**	**1**	**23**	**65**

2b. Necropsies

Summary adapted from Limpus et al 2017. Necropsy based assessment of reproductive biology of nesting green turtles at Raine Island, 2016-2017 breeding season (Draft report)

A nesting female turtle expends a considerable amount of energy when she comes ashore and digs to make a nest. If she fails to successfully lay her eggs and returns to the sea, she can be expected to return for another attempt to lay the same clutch of eggs on the same night or within the next few nights. If she is repetitively unsuccessful in laying her eggs, she will deplete her stored fat reserves over a series of nights. Her capacity to replace those fat reserves during the nesting season is compromised by the female not feeding or feeding to a negligible amount during the inter-nesting periods. When a nesting female excessively reduces her stored fat reserves, she will commence to extract nutrients and energy from the mature ovarian follicles to support her immediate needs and to fuel her return migration back to her home foraging area. Once a mature follicle is being resorbed, i.e. undergoing atresia, it is no longer available for ovulation to make an egg. For a turtle that is repeatedly confronted with nesting habitat that impedes her capacity to successfully lay eggs, quantifying the proportion of large atretic follicles in an ovary can therefore provide a measure of the proportional reduction in remaining egg production for the turtle for that breeding season.

These methods provide a direct measure of:

- recruitment rate of new breeding females into the annual breeding population of marine turtles;
- nesting success of females ashore to lay their eggs;
- reduction in the number of clutches laid per female within a breeding season.

Methods:

When the study team was at Raine Island during the 2-11 November 2016 and 30 November – 5 December 2016 study periods, daily monitoring was conducted of dead or moribund turtles. Live moribund turtles were rescued as they were identified and returned to the sea following standard NPSR management at the island. As a result of this management intervention, the availability of freshly dead turtles is limited on current trips and freshly dead turtles were most likely to be available on the day the team arrived at the island for each trip.

Freshly dead turtles were necropsied to assess breeding condition as follows. The turtle carcass was rolled on its carapace and either the plastron was removed to expose the internal organs or an incision was made immediately anterior to a rear flipper to access the body cavity. A portion of the gonad and the associated oviduct was removed to the exterior of the turtle.

The ovary or portion of the ovary was spread on a flat surface such as the turtle’s plastron for photography. Each turtle was identified by its flipper tag number or by a printed specimen number with an “N” prefix. The printed specimen number was placed on the surface of the ovary for photography

Photographs were taken using a digital camera set at ISO 1,600 or higher. Images were checked for sharpness while ensuring that structures as small as 2mm in diameter were discernible. Eggs in left and/or right oviducts were counted.

If time permitted, the crop and stomach was examined for food content.

After being returned to the laboratory, the photographs were examined independently by two persons with advanced experience in identification of gonad morphology (Dr Colin Limpus, Duncan Limpus) to determine the presence of corpora albicantia and atretic and mature ovarian follicles. The number of mature ovarian follicles and mature sized atretic follicles were counted in one image of the gonad sample for each turtle.

Percentage estimates are summarised with ± 95% confidence limits.

Results:

The gonads and oviducts of 14 freshly dead green turtles that had died while ashore on nesting crawls at Raine Island were examined from 16 attempted necropsies during the 2016-2017 nesting season (Table 6). Necropsies were discontinued with two turtles that were too decomposed to provide useful data.

Table 6. *Summary of results of necropsy of freshly dead female Chelonia mydas that had died on the beach platform following nesting attempts during a previous night on Raine Island, 2 November - 04 December 2016. 'Yes' denotes presence; 'Nil' denotes absence; "-" denotes not recorded.*

Date 2016	Tag no.	CCL (cm)	Oviducal eggs		Corpora albicantia	Mature follicles in atresia	Notes
			Left	right			
02 Nov	N86826	107.8	39	44	Yes	Nil (0/5)	• Spent ovary;<br>• Nil stomach content
07 Nov	N86827	102.6	0	0	Yes	Yes (46/69)	• Died after only ovulating 1 clutch which was successfully laid on this night.
30 Nov	N86828	98.7	52	-	Yes	Yes (7/22)	
30 Nov	N86829	105.7	-	42	Yes	Nil (0/33)	
30 Nov	N86830	108.8	69	-	Yes	Yes (7/42)	
30 Nov	N86831	103.3	-	50	Yes	Yes (7/42)	
30 Nov	N86832	102.3	0	-	Yes	Yes (6/35)	
30 Nov	N86833	99.8	-	46	Yes	Yes (4/21)	
30 Nov	N86834	103.3	-	50	Yes	Yes (26/40)	
30 Nov	N86835	109.7	-	45	Yes	Yes (8/37)	
30 Nov	QA8291<br>QA66030<br>N86836	114.0	-	0	Yes	Yes (8/38	• Nesting Raine Is. Nov 2009.<br>• Agreement of gonad & capture data
30 Nov	QA74806<br>N86837	111.1	-	27	Yes	Yes (3/48)	• First tagged at Raine Is. Dec 2016
02 Dec	N86839	106.1	35	-	Yes	Yes (4/29)	
04 Dec	N86840	102.3	46	-	Yes	Yes (5/31)	

Presence or absence of oviducal eggs was scored for 14 turtles. Only 3 (21 ± 21%) had completed oviposition (Table 2). The remainder carried oviducal eggs consistent with having laid no eggs while ashore for nesting during the night before they died or they had been disturbed, presumably by another turtle(s) before they had completed the laying of the full clutch of eggs. This represents a low nesting success for these nesting females.

Twelve (86 ± 18%) of the 14 ovaries examined contained mature follicles that had begun atresia (Table 6). For the turtles for which gonads were examined during 30 November – 5 December 2016, the mean proportion of large

ovarian follicles that had commenced atresia per female was 21% (Table 2. n = 12, range = 0 – 65%). Based on an average female green turtle within the nGBR genetic stock laying on average of 6 clutches of 102 eggs per breeding season (Limpus *et al.* 2001), this level of mature follicle reduction would be equivalent to a reduction of at least one clutch of eggs for the entire season for these females, if they had not died at that time.

Considering female N86827 which was necropsied on 7 November 2016, this turtle had successfully laid the only clutch that she had ovulated for the season but had commenced resorption of 67% of her mature ovarian follicles. This turtle could only have produced one more clutch of eggs had she survived to lay additional eggs for the season. Assuming that this turtle was an average egg producer for this population, the loss of mature follicles due to atresia was equivalent to the loss of four clutches of for eggs for the season.

The 14 turtles examined by necropsy were scored for evidence of a past breeding history based on presence or absence of corpora albicantia in the ovaries. Both assessors scored each gonad identically. The one female with a past recorded breeding history based on tagging-recapture history was correctly scored via gonad examination as having bred in a previous breeding season. Corpora albicantia were found in all 14 females. This result, although from a small sample of the nesting population, suggests that there was a very low a recruitment rate, approaching 0**%** of new females into the 2016-2017 breeding population.

Discussion:

This season's measured rate of atresia of mature ovarian follicles in the early nesting season (late November – early December) is equivalent to a reduction by one in overall clutch production per female per year and should be viewed with concern. There is a high probability that, with continued poor nesting success through the remainder of the breeding season, there would have been an overall reduction in clutch production within this nesting population by several clutches per female. If such a reduction in annual egg production is occurring, then the current green turtle nesting population of Raine Island and Moulter Cay will not be sustainable, irrespective of the hatching success of the eggs that are laid.

This high incidence of atresia among nesting females early in the breeding season has not been recorded at any Australian turtle rookery other than Raine Island.

These measures of nesting success and follicular resorption (atresia) provide a direct measure of management success of green turtle breeding on Raine Island that is independent of hatching success of eggs laid.

The current measures of breeding success obtained via gonad assessment are indicative of breeding success not being managed successfully at Raine Island. Therefore while management actions continue to address the poor breeding success of the Raine Island green turtle population, increased emphasis should be given to improving the quantification of these parameters that give definitive measures of nesting success and loss of egg production during annual monitoring of the nesting population by:

- significantly increasing the number of turtles being examined;
- increasing the sampling events to include early, mid and late breeding season;
- increasing the range of techniques applied to measure these parameters to include gonad examination via necropsy of freshly dead turtles and viewing of gonads of the nesting females via laparoscopic and/or ultrasound examinations (Limpus *et al*. 2003, 2005). There will be a deceasing quality of data obtained from these three approaches to assessing breeding via gonad examination.

2c. Cliff-top fencing - mitigation of adult turtle mortality from cliff-falls

To minimise adult turtle mortality from cliff falls, cliff top fencing has been installed since 2011 in high-risk cliff areas.

A further 150m of cliff top fencing was installed in July 2016, bringing the cumulative total of fencing on the Island to 1150m (Figure 2). The 150m of fencing was installed along the cliff top, at the southern end of the island, adjacent to the re-profiled sector and directly behind the majority of the re-profiled sector. The site was chosen due to its proximity to the re-profiled sector and based on historical cliff fall data. Following the installation of the cliff top fencing during the 2016-17 season, minimal cliff fall deaths were recorded in this area. Cliff fall deaths were concentrated in an area near the tower with outlying raised rocks which are as yet not treated to minimise falls (Figure 3). Other significant mortality locations were in areas with no fencing or damaged fencing due to cliff erosion.

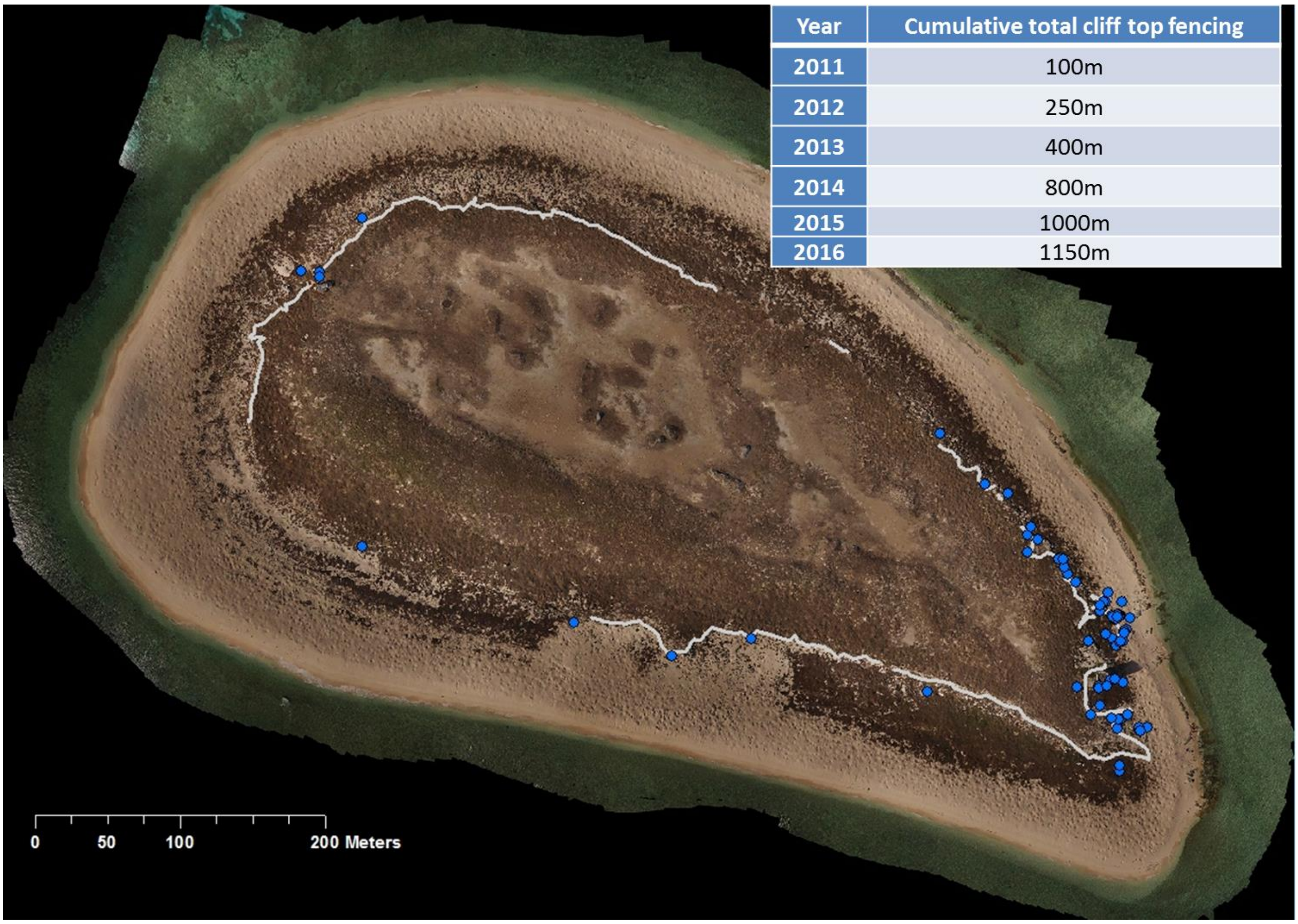

Figure 2. *Cliff top fencing at Raine Island as at December 2016. The current 1150m of fencing along the clifftop is marked as a white line. Location of cliff fall deaths are shown as blue dots.*

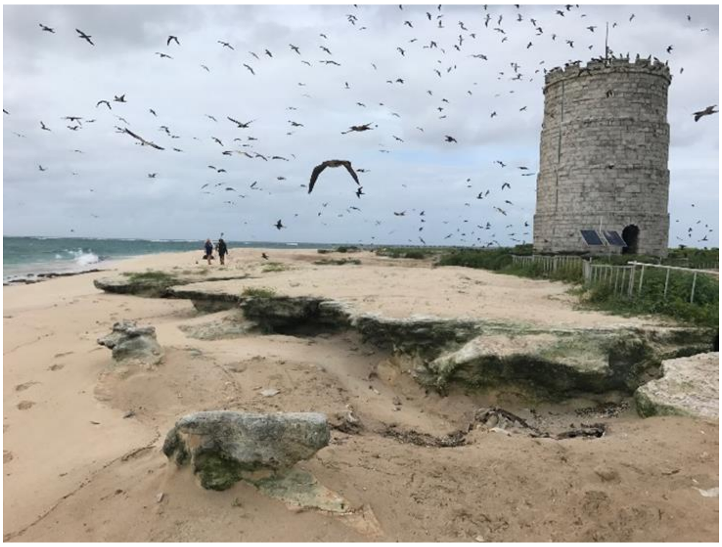

Figure 3. *Site of majority of cliff fall deaths in 2016-17*

2d. Turtle rescues

There were a total of 161 adult female turtles rescued at Raine Island in 2016-17 and 12 rescues at Moulter Cay (Table 7).

Table 7: *Turtle rescues and causes for rescue for 2016-17*

Date	Heat	Cliff falls	Cliff entrapment	Beachrock	TOTAL
**RAINE**					
Nov 2-11, 2016	19	3	6	10	38
Nov 30 - Dec 11, 2016	47	11	2	4	64
Jan 29 - Feb 9, 2017	13	27	19	0	59
April 4 - 9, 2017	0	0	0	0	0
**TOTAL**	**79**	**41**	**27**	**14**	**161**
**MOULTER**					
Feb 12-14, 2017	4	1	0	7	**12**

3. NESTING ACTIVITY

Methods

Nesting success is defined as the proportion of females ashore for the night that lay eggs (Limpus et al. 2003). Two study sectors on opposite sides of Raine Island (Sectors A2 & B) and the re-profiled sector (Fig. 4) were monitored simultaneously for four nights in early November and 4 nights in late Nov / early Dec, 2016 and three nights in late Jan/early Feb, 2017. Sector A2 is slightly to the south east of previously monitored Sector A in years prior to 2013 and hence some caution must be used in comparison of results across the years, although similar aspect and topography apply.

Each site was 50m in width and extended perpendicularly from the tidal water line to the cliff face. Each sector was marked with posts at the lateral edges and centre line to demarcate sub-sectors identified as berm, back berm, swale and back swale (0-15m, 15-35m, 35-55m and 55-75m from the top of the berm towards the cliff, respectively) (Fig.5). To minimise disturbance to nesting turtles no other activities were conducted within or adjacent to the survey sectors through the entire night. Within each site a count was made of the number of:

- Turtles which entered from the water line and from either side;
- Clutches laid and their sub-sector location;
- Existing clutches dug into by these nesting turtles; and
- Turtles within each of the subsectors at intervals of two hours throughout the entire night.

Turtles within the study sector were marked on the carapace with a paint spot on entry, exit and re-entry to the sector to gain an accurate total count of nesters within the sector. Turtles that successfully nested were painted with an X on the rear of the carapace to ensure each successful nesting was recorded only once.

Each site required two staff continuously monitoring the turtle nesting for approximately 12hr commencing at 1800hrs. Two rate parameters (nesting success and clutch disturbance by nesting turtles) were calculated from these data:

Nesting success = (no. clutches laid) / (total no. turtles entering area) * 100 %.

Clutch disturbance by nesting turtles (CD) = (no. existing clutches disturbed) / (no. new clutches laid).

Nesting success was calculated using the number of turtles entering the area by crossing the high water line. It is assumed that lateral movements along the beach into and out of a sampling site of turtles attempting to nest are approximately random and should cancel each other.

Clutch disturbance counts provide a measure of the egg mortality per disturbed clutch (EMpDC) caused by nesting females. When compared to the mean clutch count for the rookery, this egg destruction can be expressed as the proportion of a clutch destroyed per clutch laid (clutch equivalent egg mortality = CEEM).

CEEM = (EMpDC)(CD) / (mean clutch count).

The mean clutch count of 104.3 eggs measured in past studies (Limpus et al. 2003) were used for this analysis.

Bi-hourly counts of turtles and the successful nesting efforts within the sub-sectors were analysed to determine distribution across the beach of turtles attempting to nest and those which successfully nested.

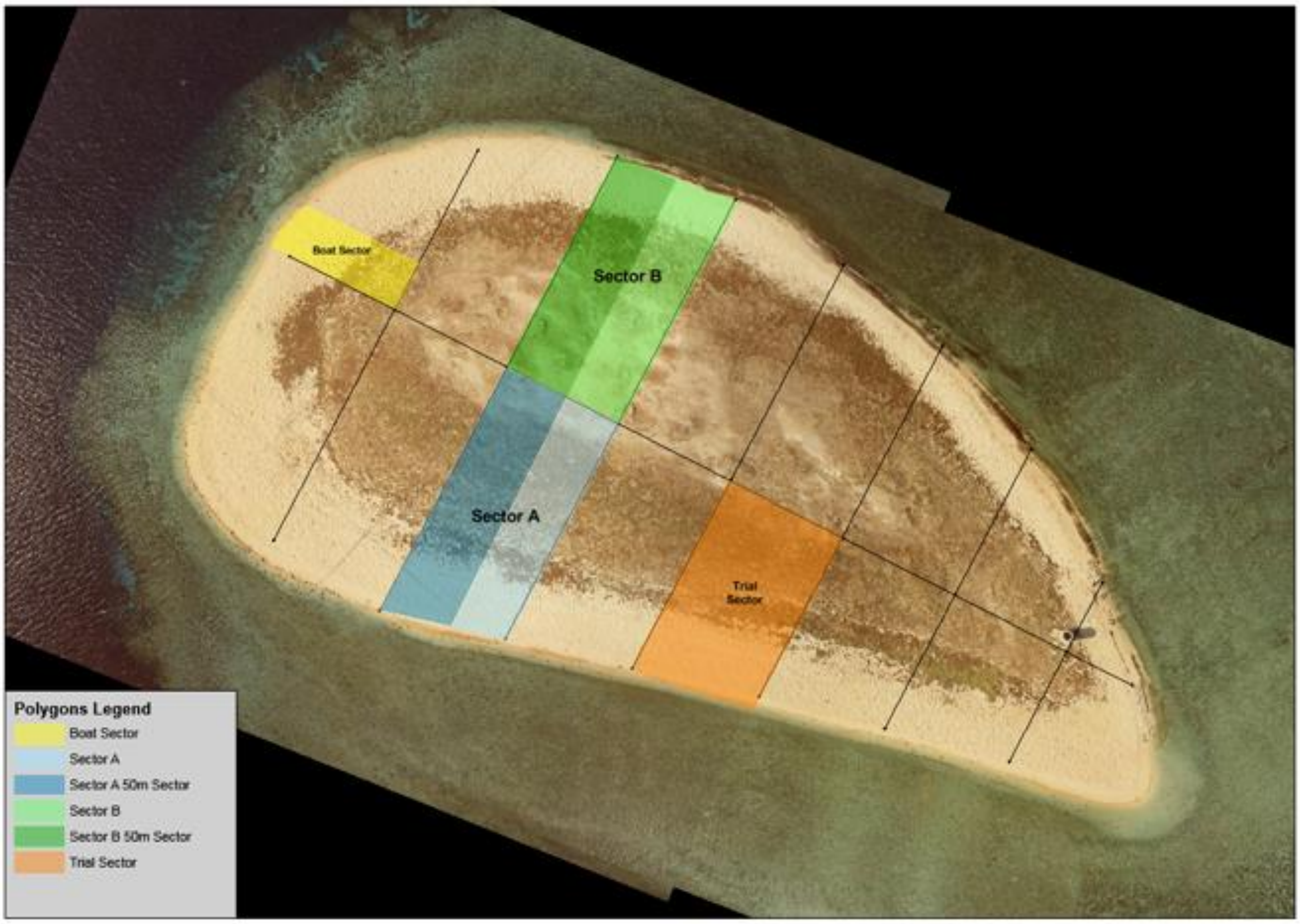

Figure 4. *Raine Island nesting and hatching success survey sectors*

50m wide sectors A2 & B and Re-profiled sector were monitored for nesting (Nov, Dec and Jan) and hatching (Jan-Feb and Apr) success in 2016-17.

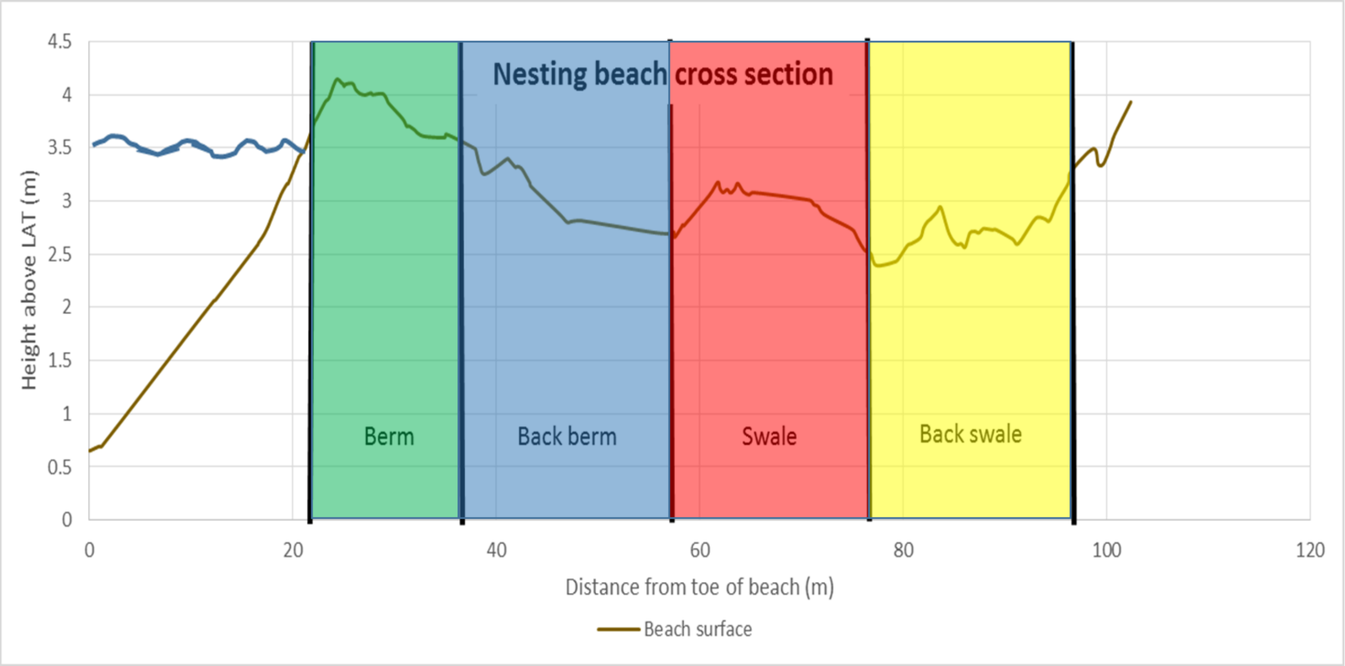

Figure 5. *Division of nesting area for nest location*

Brown line represents beach surface topography in Sector A2. Blue lines show the borders parallel to the shoreline of the subsectors across the nesting beach.

Results

3a. Nesting success

Nesting success was around the 20% early season average for Raine Island in early November. Sand was much drier in Sectors A and C than in Sector B which had been eroded by northerly winds and overtopped in August. This may have been the reason for increased sand moisture observed during the Nov and December nesting period in Sector B and the higher nesting success recorded there (Fig. 6 and Table 8.).

Total nesting turtle numbers were also higher in Sector B as there was exposed beachrock on eastern end of the sector restricting entry to the nesting beach in this area which concentrated the turtles into Sector B (Table 8).

Nesting success was lowest in early December 2016 (0.08) which correlates with a very low rainfall leading up to this time and resulting in dry nesting beach sand. November (0.23) and February nesting (0.39) success suggests a strong positive correlation with rainfall levels prior to these survey periods (Figs. 6 & 8 and Table 8).

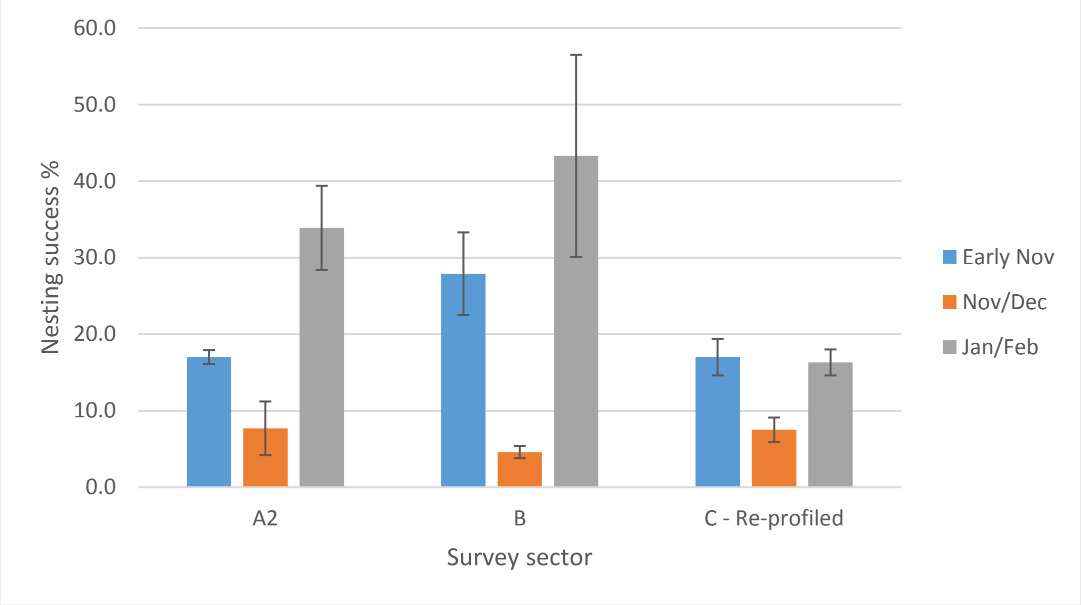

Figure 6*. Nesting success in the re-profiled sector compared to control sectors in 2016-17. Standard errors bars are as indicated.*

Table 8. *Nesting success survey summary for 2016-17*

Surveys undertaken in 50m long sectors for entire nesting nights during 2016-17 season. Data presented are calculations of the mean.

Date	Sector	# Surveys	# Nesters	# Clutches laid	Nesting success %	S.E
**Raine Island**						
Nov 3 - 9	A2	4	115.8	19.5	**17.0**	0.9
	B	4	145.3	39	**27.9**	5.4
	C - Re-profiled	4	112.3	19	**17.0**	2.4
Nov 30 - Dec 10	A2	2	335.5	25	**7.7**	3.5
	B	3	500.3	22	**4.6**	0.8
	C - Re-profiled	4	339.5	23.3	**7.5**	1.6
	D	2	295	31	**10.5**	3.0
Jan 30 - Feb 1	A2	3	65.7	20	**33.9**	5.5
	B	3	105	39.3	**43.3**	13.2
	C - Re-profiled	3	164.3	27.3	**16.3**	1.7
**Moulter Cay**						
Dec 11 - 13	A	3	178.7	27.0	**17.6**	7.2
	B	3	115.3	34.0	**30.4**	6.6
	C	3	96	18.3	**19.8**	3.1
**McLennan Cay**						
Dec 14	A	1	27	7	**25.9**	
	B	1	6	4	**66.7**	
	C	1	7	2	**28.6**	

3b. Distribution of clutches laid within survey sectors

Turtles nesting within the re-profiled sector spread out more evenly and were more successful throughout the entire berm, back berm, swale and back swale areas when compared with the controls (Table 9 and Figs. 7a, b & c). Nesting occurs predominantly in the berm area in the early part of the season. Re-profiled sector C shows a broader distribution of nests and higher percentage of nests throughout the swale and back swale in early December and early February except in comparison with Sector B in Dec 2016. (Figs. 7a, b & c)

Refer to nesting beach cross section (Fig.2) for location of berm, back berm, swale and back swale areas.

Table 9. *Nesting success data for 2016-17 season*

Date	Sector	# Surveys	# Clutches laid (mean)					% of Total clutches laid			
			Berm	Back berm	Swale	Back swale	Total	Berm	Back berm	Swale	Back swale
**Raine**											
Nov 3 - 9	A2	4	19.0	1	0	0	20.0	95.0	5.0	0.0	0.0
	B	4	31.0	8	1	0	40.0	77.5	20.0	2.5	0.0
	C - Re-profiled	4	17.0	2	1	0	20.0	85.0	10.0	5.0	0.0
Nov 30 - Dec 10	A2	2	19.0	0.5	0	0	19.5	97.4	2.6	0.0	0.0
	B	3	30.8	7.5	0.8	0	39.1	78.8	19.2	2.0	0.0
	C - Re-profiled	4	16.8	1.8	0.5	0	19.1	88.0	9.4	2.6	0.0
	D	2	18.0	5.5	5.5	2.5	31.5	57.1	17.5	17.5	7.9
Jan 30 - Feb 1	A2	3	7.3	8.0	3.7	1.0	20.0	36.7	40.0	18.3	5.0
	B	3	15.7	11.0	12.7	0.0	39.3	39.9	28.0	32.2	0.0
	C - Re-profiled	3	7.3	10.0	5.3	4.7	27.3	26.9	36.6	19.5	17.1
**Moulter**											
Dec 11 - 13	A	3	14.7	5.7	6.7		27.0	54.3	21.0	24.7	0.0
	B	3	22.7	10.7	0.7		34.0	66.7	31.4	2.0	0.0
	C	3	12.3	5.7	0.7		18.7	66.0	30.3	3.6	0.0

Figure 7a.

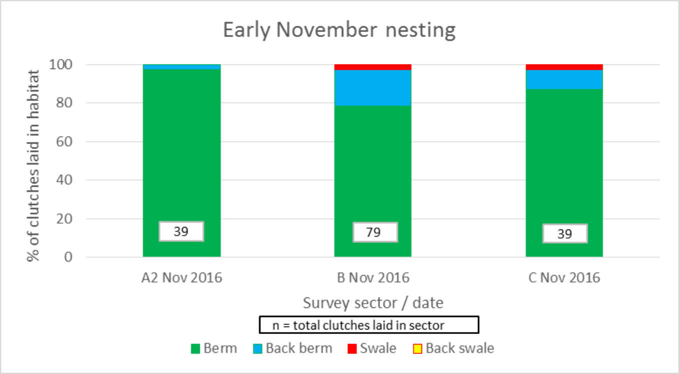

Figure 7b.

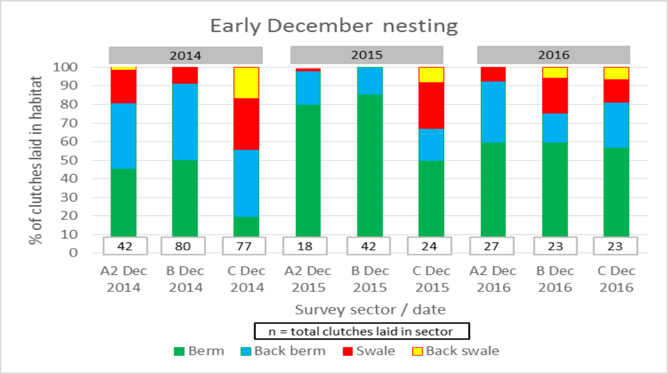

Figure 7c.

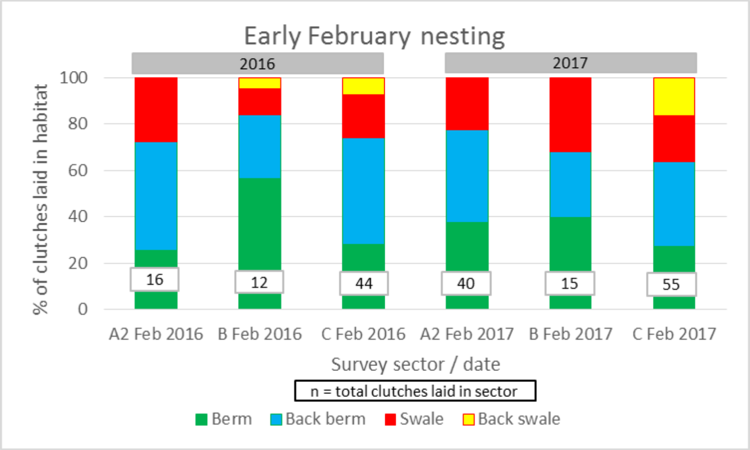

Figures 7. (a, b &c). *Mean number of clutches laid per night and distribution of nests within survey sectors*

Mean number of clutches laid per night is presented in boxes. % of clutches within each habitat type is presented as per the table legend.

Rainfall influence on nesting success

Nesting success was lowest in early December 2016 (0.08) which correlates with a very low rainfall leading up to this time and resulting in dry nesting beach sand. November (0.23) and February nesting (0.39) success suggests a strong positive correlation with rainfall levels prior to these survey periods (Fig. 8).

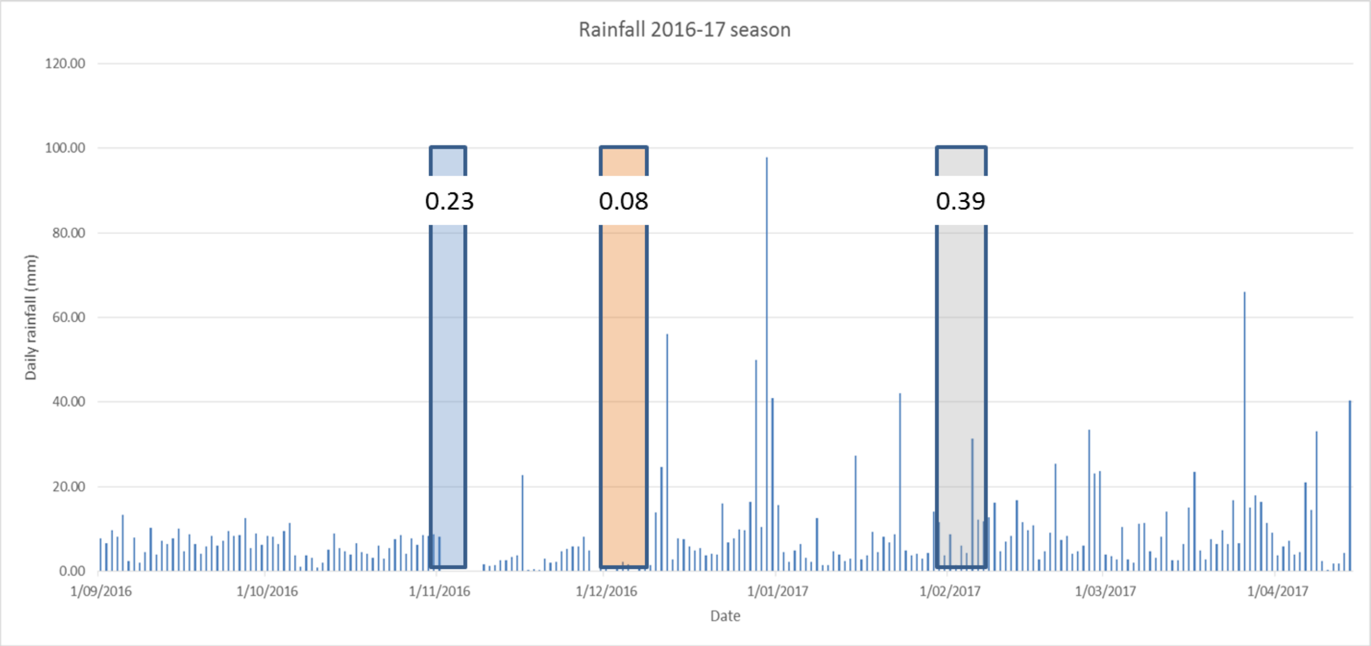

Figure 8. *Daily rainfall levels compared with nesting success in un-modified beach areas for 2016-17*

4. HATCHING SUCCESS

Method:

dGPS clutch marking

Clutches were marked as they were laid using a Trimble differential GP roving unit (dGPS) linked to a base station sited at the north-western end of the island. When a turtle was observed to be finalising its egg chamber the surveyor was signalled over to mark the nest. The dGPS rover unit pole was placed vertically on the rear carapace of the nesting turtle directly above the nest and easting and northing positions were recorded. The height from the base of the pole (on the turtle carapace) to the bottom of the nest was measured using a fibreglass tape measure. A final elevation was recorded for the bottom of each clutch/nest. Ping-pong balls labelled with the nest ID were placed centrally within the clutch to ensure accurate clutch identification upon excavation of nests post-hatching.

Hatching success

Nests marked by dGPS were re-found using the easting, northing and elevation co-ordinates for each nest. The nest site was marked with a surveyor flag marker labelled with nest ID and depth of the top of the eggs below the current surface level. This provided an accurate means to relocate the nests. Clutches were excavated by hand digging > 60 days after laying.

Once the clutch was exposed eggs were removed and sorted into hatched and unhatched eggs and dead hatchlings. Hatching success of an excavated nest is defined in this report as the percentage of successfully hatched eggs emerged as hatchlings from a nest, where:

% hatching success = (hatched/(hatched+unhatched)) x 100%

Emergence success is calculated as = (hatched - (live in nest +dead in nest))/(hatched+unhatched)) x 100%

At Raine Island the numbers of live and dead hatchlings in the nest is extremely low and for succinctness in reporting here, hatching success only is presented.

For n = 587 nests excavated between the 2011-12 and 2016-17 seasons where live and dead hatchlings were

recorded

Mean number of live hatchlings per nest = 1.1 ± 0.2

Mean number of dead hatchlings per nest = 0.3 ± 0.1

Live within nest hatchlings were in almost all cases were fit and viable and expected to emerge successfully. In a small number of cases obstructions had prevented emergence and hatchlings were alive but compromised and not expected to emerge successfully.

Results

Prior to 2011-12 season emergence success studies were conducted by exhuming nests from which emergences had been detected, rather than by marking nests clutches when laid and exhuming post-hatching time. Data from the 2001-02, 2009-10 and 2010-11 season studies show similar results to the more recent studies with high variability between individual clutch hatching success and between-season mean hatching success (Table 10).

Results for the 2011-12 to 2016-17 seasons show low hatching success (24.6%) in the highest nesting population year (Dec 2013) and higher hatching success in re-profiled sector than in un-modified areas in all but Dec 2014 survey periods (Table 11 and Fig 9).

Mapping locations of clutches with hatching success shows low hatching success in December 2013 (Fig 10c) in all areas and low hatching success at the western end of the nesting beach throughout all survey periods.(Fig 10a to i)

Hatching success in 2016-17 (Table 5 highlighted in yellow) is relatively high (for Raine Island) at around 60% and with a higher hatching success in the re-profiled sector (61.2%) compared with un-modified areas (46.5%) only during the later Feb – April period of incubation.

The mapping of clutch hatching success for 2016-17 (Fig 10i) shows the higher success throughout all habitats of the re-profiled sector while in un-modified areas the swale and back swale show consistently low success.

Mapping of clutch hatching success of nests in areas where beach replenishment has not taken place (including the re-profiled sector area prior to its re-profiling in 2014) demonstrates marked variability in hatching success throughout. Nests have a wide range of hatching success throughout all beach habitats and locations around the island.

The beach nesting area at the north-western (37.5%) and south-eastern ends (31.7%) of the island show a lower hatching success than the remainder of the nesting beach (51.3%) (Table 13 and Figures 10a to i)

A comparison of hatching success in un-modified areas with total estimated breeding turtle numbers (early December estimate) is shown in Table 12 and Figure 11. This shows a strong indication that hatching success is inversely related to nesting turtle numbers.

Table 10. *Hatching success from 2001-02, 2009-10 and 2010-11 seasons*

Date laid	Number of nests sampled	Hatching success	Standard error	Range
Dec 2001	13	49.7%	9.0	0 – 100%
Dec 2009	39	78.21%	± 3.8	17.6 – 100%
Dec 2010	175	49.6%	± 1.6	0 – 100%

Table 11. *Hatching success – R in sampling date indicates re-profiled Sector C*

	Number nests sampled	Recovered clutch size		Hatching success %		Nesting population size (Dec)	
	(>30 eggs recovered / clutch)	Mean	S.E	**Mean**	S.E	**Mean**	S.E
All years total	607	85.9	1.0	**51.9**	1.3		
2011 Dec	30	87.5	5.0	**40.6**	7.6	**17167**	± 1632.7
2012 Dec	68	83.7	2.4	**49.6**	3.5	**3600**	
2013 Dec	73	84.1	3.4	**24.6**	3.1	**59933**	± 7024.3
2014 Dec	24	76.0	5.0	**59.4**	4.7	**14439**	± 1174.1
2014 Dec - R	35	83.6	4.6	**51.3**	3.9	**14439**	± 1174.1
2015 Feb	47	90.4	3.3	**46.1**	4.8	**14439**	± 1174.1
2015 Feb - R	19	95.5	4.7	**56.3**	9.4	**14439**	± 1174.1
2015 Dec	56	82.8	3.1	**66.6**	3.4	**5805**	± 88
2015 Dec - R	11	85.4	5.8	**85**	3.1	**5805**	± 88
2016 Feb	46	100.8	3.4	**56.1**	4.8	**5805**	± 88
2016 Feb - R	27	98.4	3.9	**75.9**	4.2	**5805**	± 88
2016 Dec	76	75.6	3.0	**59.4**	3.8	**11960**	± 534.2
2016 Dec - R	13	67.2	8.3	**62**	8.7	**11960**	± 534.2
2017 Feb	69	91.8	2.4	**46.5**	3.6	**11960**	± 534.2
2017 Feb - R	12	96.3	7.2	**61.2**	7.2	**11960**	± 534.2

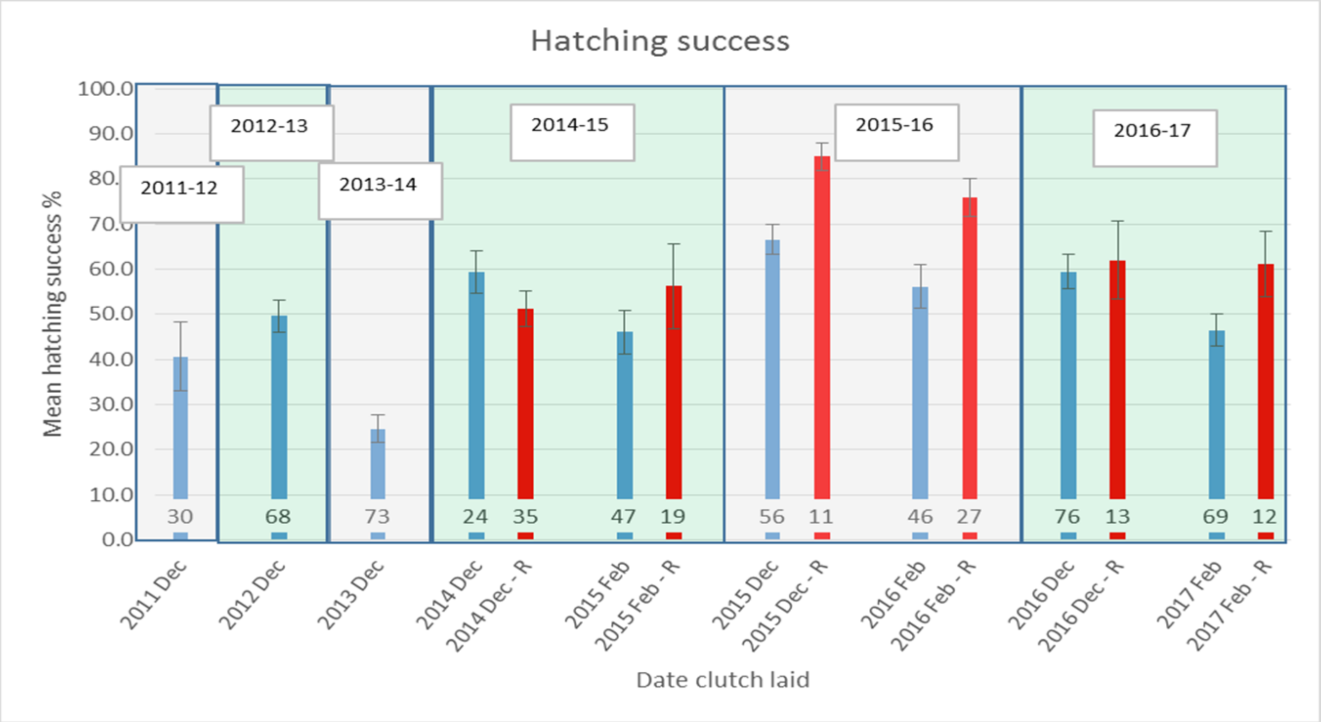

Figure 9. *Hatching success summary and comparison of re-profiled Sector C with un-modified areas*

Fig 10a.

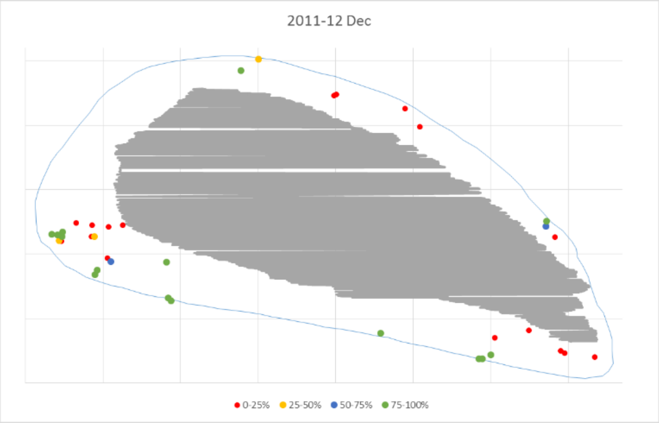

Fig 10b.

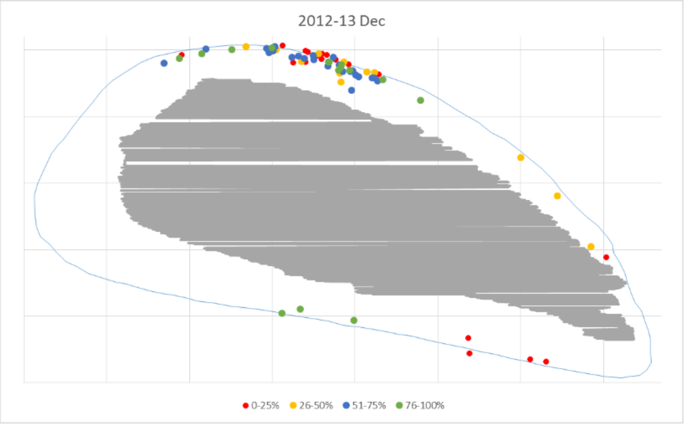

Fig 10c.

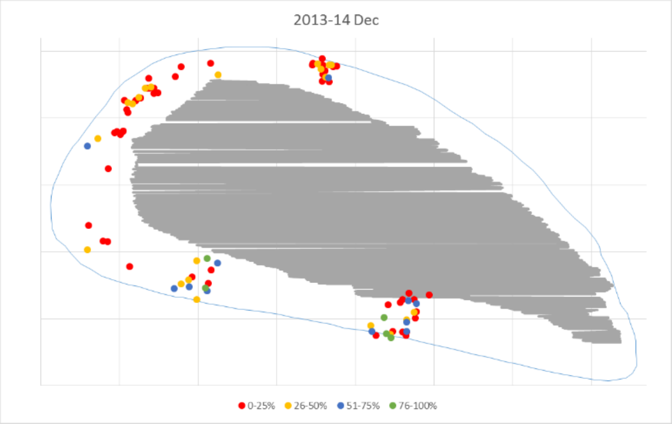

Fig 10d.

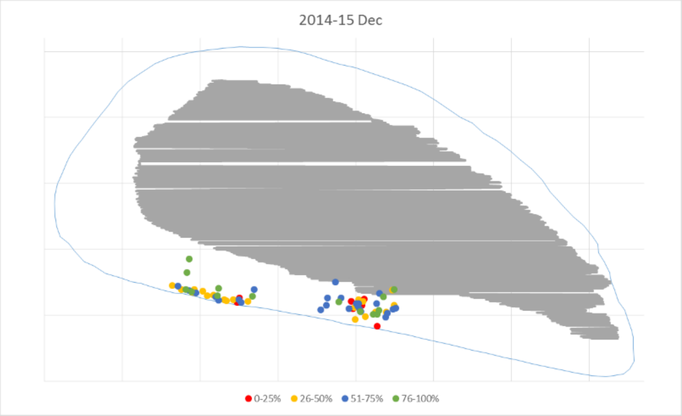

Fig 10e.

Fig 10f.

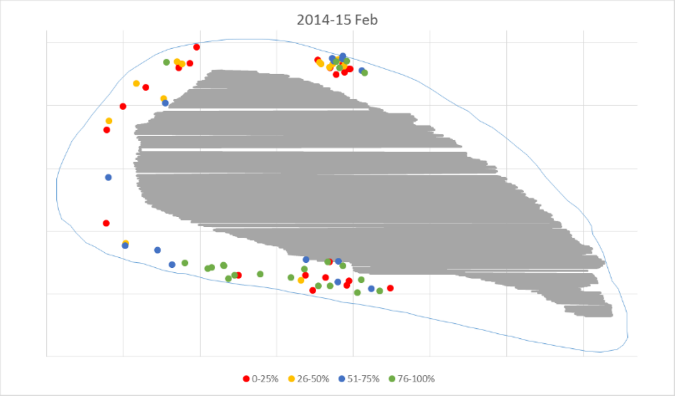

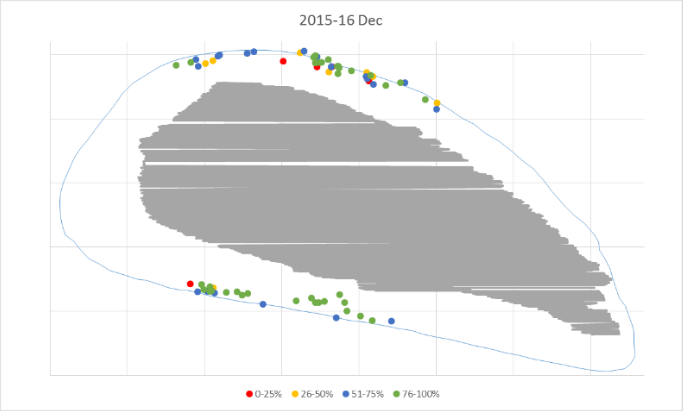

Fig 10g.

Fig 10h.

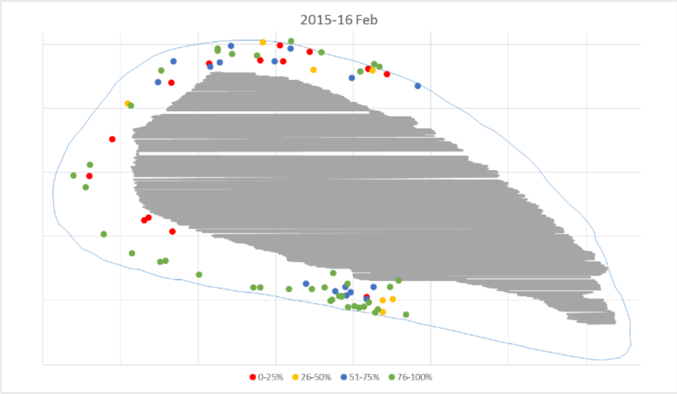

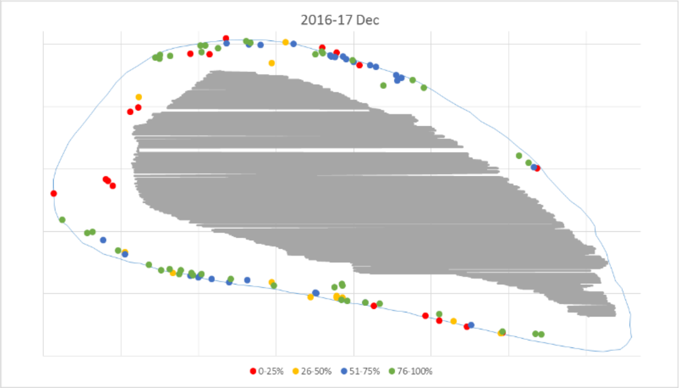

Fig 10i.

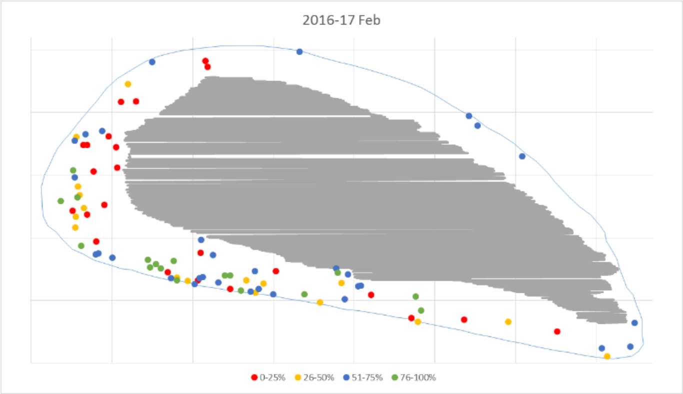

Figures 10. (a, b, c, d, e, f, g, h & i). *Maps of hatching success throughout the nesting beach for each survey period.*

Survey period is identified as date clutch laid, with excavation and analysis of clutch hatching success and timing of embryonic death conducted 60 days later. Maps are oriented to compass standards.

Table 12. *Nesting population size compared with hatching success from survey sectors with unchanged beach profile (non re-profiled areas).*

Survey season	Nesting population size	S.E	Hatching success (%)	S.E
2011-12	17000	±1632.7	48.6%	±7.0
2012-13	3600	±154.3	48.5%	±3.4
2103-14	59933	±7024.3	23.8%	±3.2
2014-15	14537	±1174.1	50.3%	±3.4
2015-16	5804	±88	61.8%	±2.9
2016-17	11960	±534.2	46.2%	±3.8

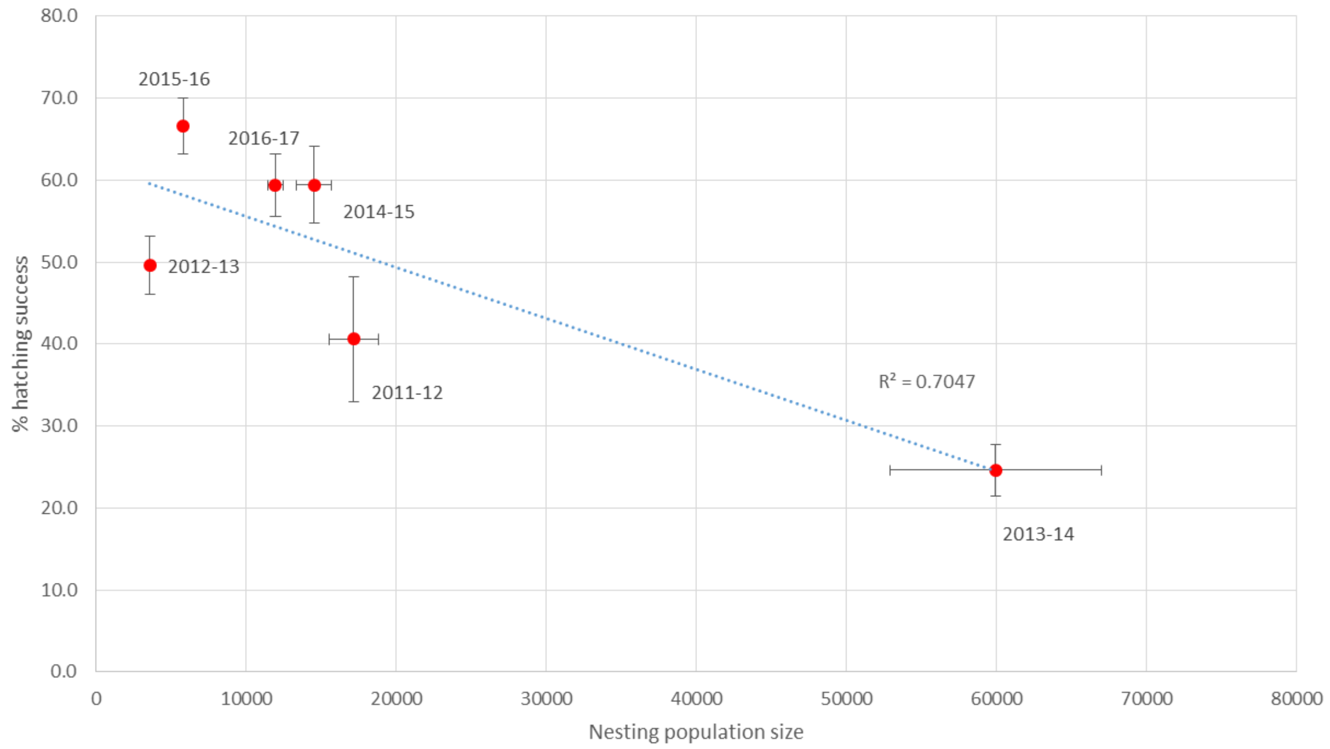

Figure 11. *Hatching success influence by nesting population size*

Table 13. *Reduced nesting success in NW and SE sections of the Raine Island nesting beach*

2011-12 to 2016-17			
	**North-western end nests**	**South eastern end nests**	**All other areas**
**Mean hatching success (%)**	**37.5**	**31.7**	**51.3**
S.E	± 4.2	± 8.2	± 1.9
Number nests sampled (n)	71	21	286

4b. Clutch destruction

Clutch destruction is higher in the early Dec - Feb period of the season compared to Feb – April incubation period (Table 14). During Dec – Feb period, especially in early December, nightly nesting turtle numbers are much higher due to low nesting success and high re-nesting effort (Fig 7 and Table 9). During the Dec - Feb period turtle nesting is also more concentrated in the berm area compared with a greater spread throughout the nesting beach later in the season (Fig 9).

During the Feb – April period clutch destruction is much lower in the re-profiled sector than in un-modified areas which may correlate with a more even distribution of nesting effort throughout the nesting beach in the re-profiled area (Fig 9 and Table 14)

Table 14. *Comparison of clutch destruction between un-modified and re-profiled areas in 2016-17*

Incubation period	Location	Number nests	Ave. clutch size	Clutch destruction (%)
Dec – Feb 2016-17	Un-modified areas	124	48.8 ± 3.7 S.E	**53.1%**
	Re-profiled sector	28	36.7 ± 7.3 S.E	**64.8%**

Feb – April 2017	Un-modified areas	93	70.4 ± 3.4 S.E	**32.3%**
	Re-profiled sector	11	94.5 ± 10.9 S.E	**9.1%**

4c. Inundation influence on emergence success of clutches

Hatching success is compared with height above peak inundation level for the centre of marked and excavated nests for survey sectors A, B, C re-profiled and D (Fig. 12a, b, c, d).

- Sector D has the majority of examined nests from all habitats below inundation level and with low hatching success
- Sector C – re-profiled has the majority of nests above inundation level with a wide range of hatching success across all habitats (tending towards high success in comparison to other un-modified sectors)
- Sector A2 has the majority of nests above inundation level and medium-high relative hatching success. This may be biased due to the lack of nests laid / marked in the low-lying areas of the swale and back swale where inundation is known to occur
- Sector B has the majority of nests above inundation level with high hatching success, however those below inundation level demonstrate low success.

Figure 12a.

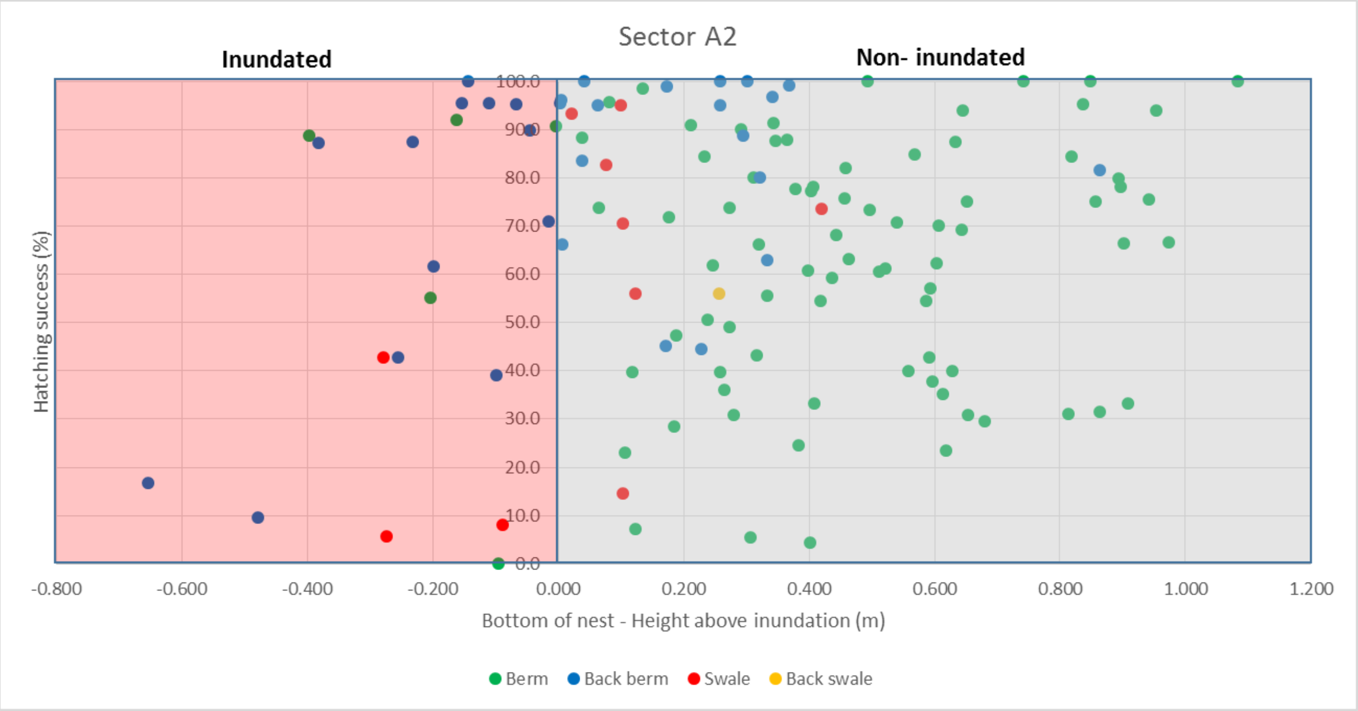

Figure 12b.

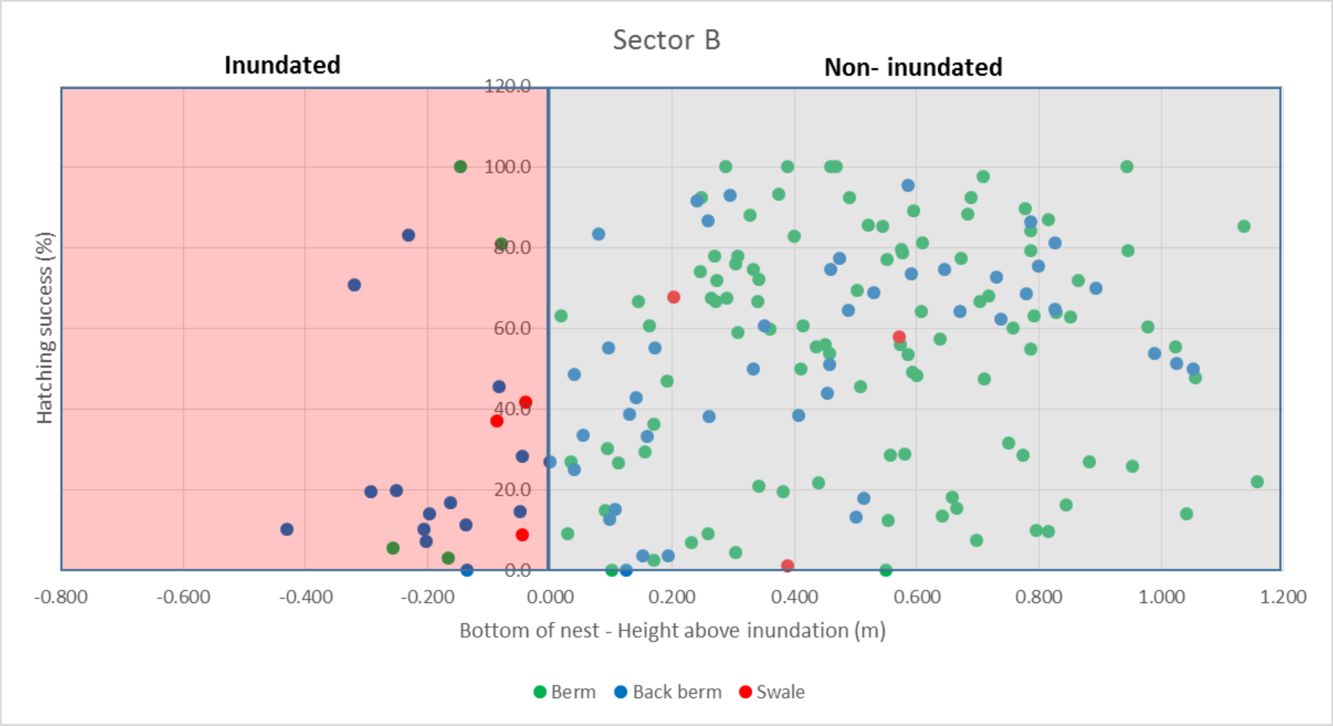

Figure 12c.

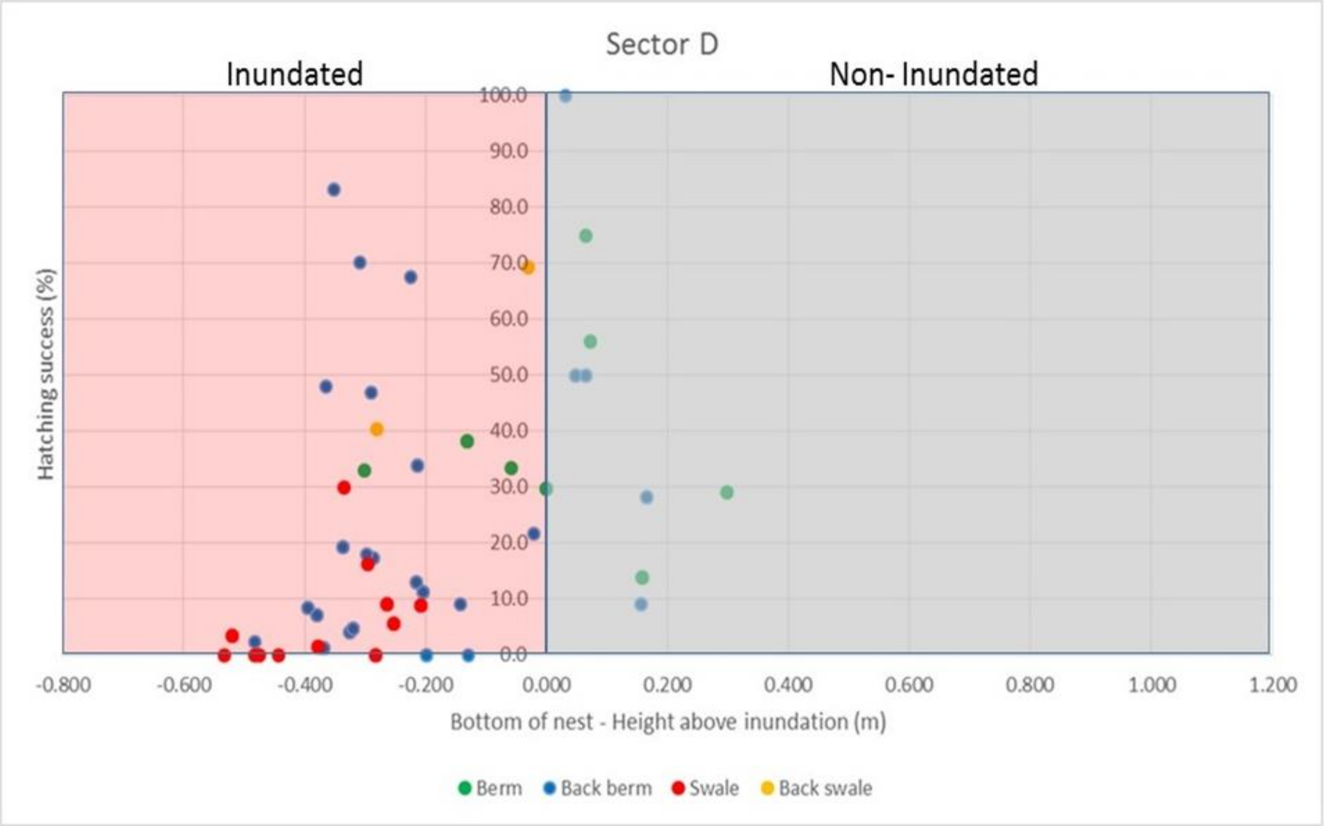

Figure 12d.

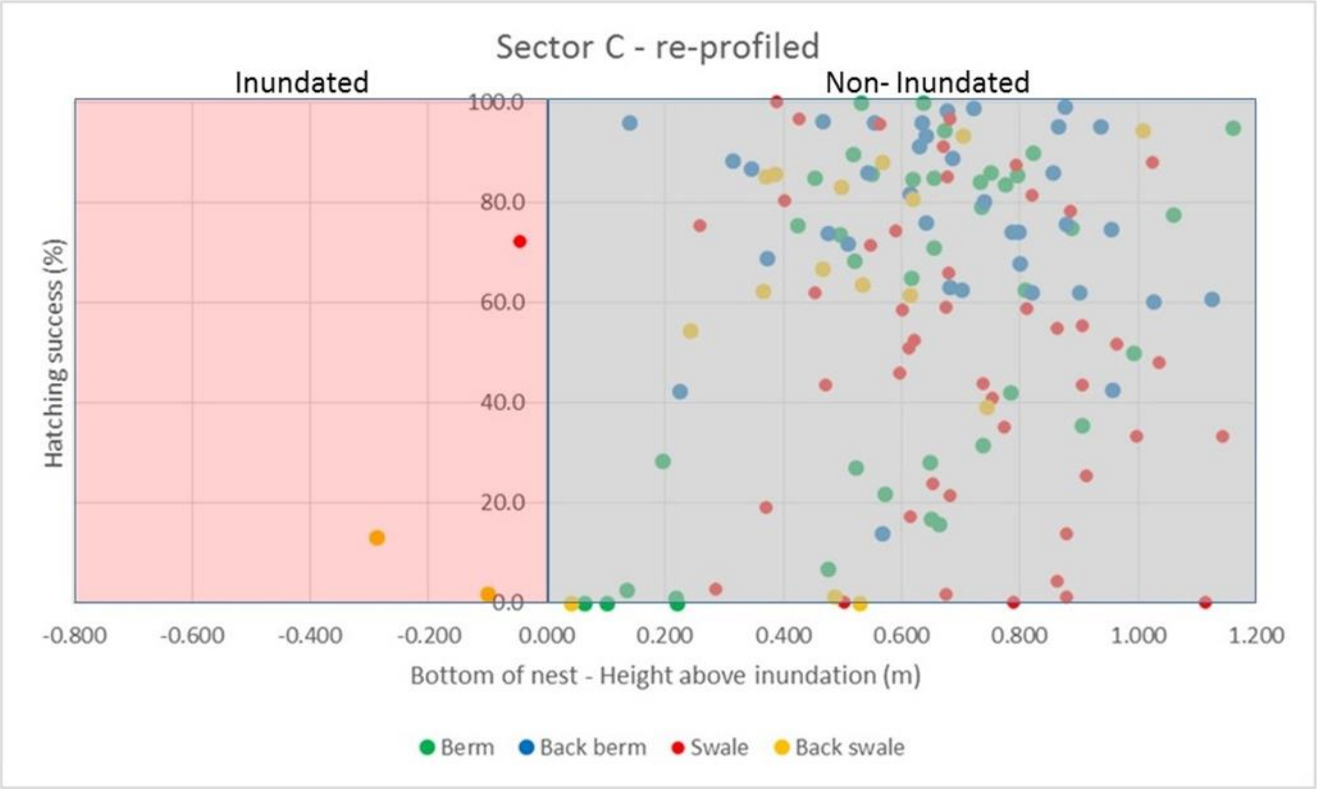

Figures 12. (a, b, c & d). *Hatching success compared with height above peak inundation level for the centre of marked and excavated nests for survey sectors A, B, C re-profiled and D*

On the horizontal axis 0 = centre of nest at peak inundation level. Negative values indicate centre of nests below peak inundation level. Habitat location of nests is designated by colour (berm – green, blue – back berm, red – swale and yellow – back swale).

Note: data presented below is not a proportionate representation of nest numbers in each habitat as nests were marked only by one surveyor covering as representative an area of the nesting beach as possible to mark turtles as they laid, only during early December and early February periods

4e. Embryo mortality

Investigations into the stage of embryonic death in hatching failure

Method:

After excavation of marked nests was completed (as described previously), unhatched eggs were then opened and the stage of development at embryonic death (1 – 6) for each egg was recorded. Stages of embryonic development were adapted from a comprehensive 32 stage key (J.Miller, J.D in review) to a simplified six phase key for field use (Table 15, Fig. 14). Eggs which were unhatched but were decomposed to an extent that precluded staging were recorded as decomposed.

Analysis of the stage of embryonic failure within clutches marked at the time of laying and excavated approximately 60 days later shows that:

- The majority of unhatched eggs have failed during stage 1, the first 0-7 days of development (Table 16, Fig. 15)
- In some seasons there were a large number of unhatched eggs recorded as decomposed. It is probable that these were early stage failed eggs. In recent samples this has been addressed more rigorously, where mid-late stage failed eggs retained evidence of bones as a minimum feature even when decomposed. (Table 16, Fig.15)

Table 15. *Raine Island field guide to stages of embryonic development in un-hatched eggs*

Developmental stage	Morphology	Developmental timeline
Stage 1	No development	0-7 days
Stage 2	Limb buds present	7-17 days
Stage 3	Carapace (shell) present but no scutes on carapace	18-23 days
Stage 4	Carapace scutes, no scales on head and flippers	24-36 days
Stage 5	Head and flipper scales, yolk bigger than turtle	37-43 days
Stage 6	Full embryo yolk smaller than turtle	44-50 days

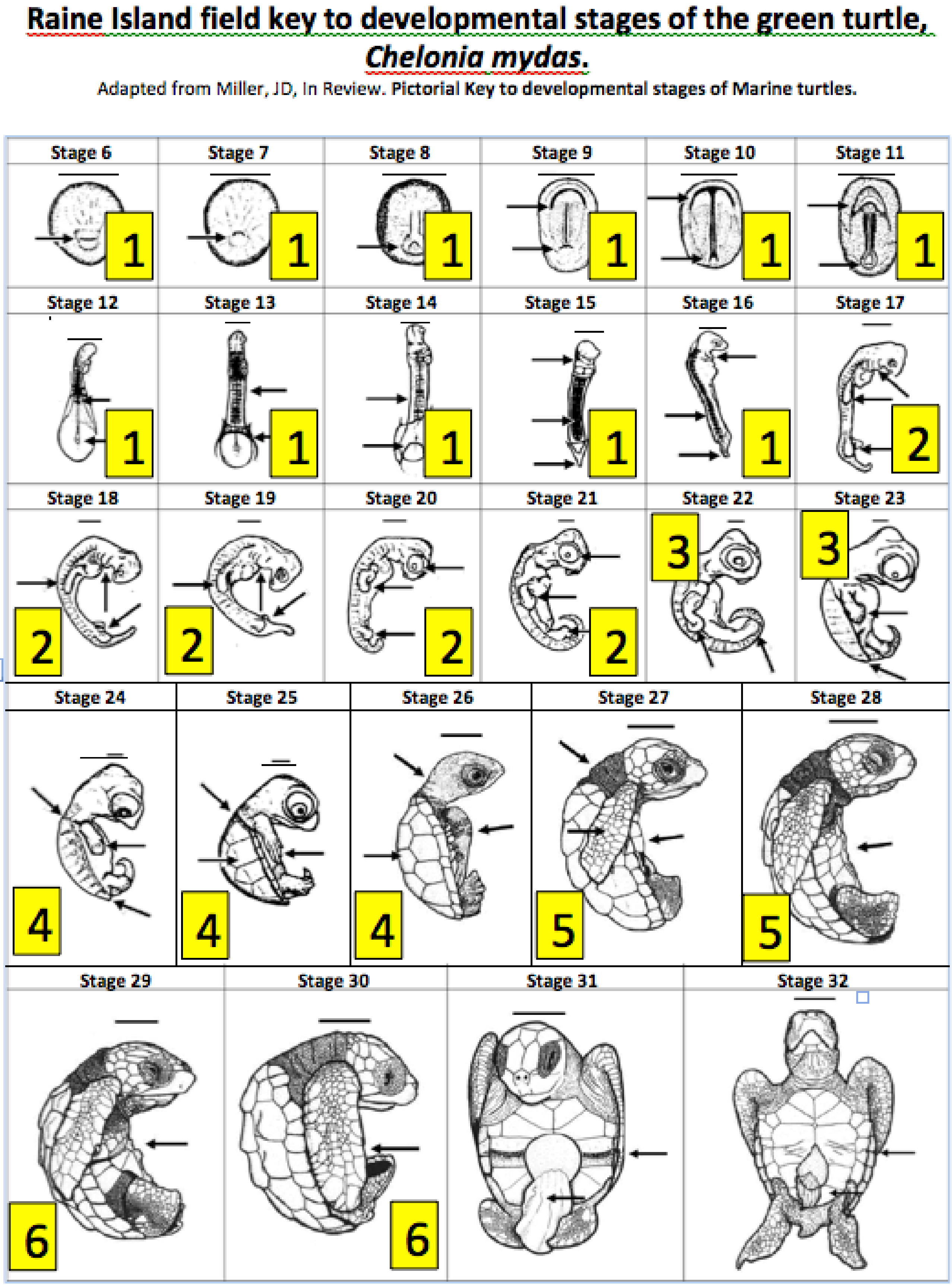

Figure 14. *Raine Island field guide to stages of embryonic development in un-hatched eggs*

Table 16. *Stage of embryonic death for clutches marked and excavated from 2013-2017*

Note: this data has not been presented here in detail for differences between clutches above and below inundation effects. This information is currently being analysed.

Date laid	n	Stage of embryonic death (% of total unhatched eggs)							% hatching success
		1	2	3	4	5	6	Decomposed	
2013 Dec	79	**62.0**	1.2	1.0	3.3	9.5	6.1	16.9	**24.0**
2014 Dec	58	**18.8**	1.3	1.3	5.8	30.0	3.0	39.9	**55.1**
2015 Feb	67	**47.9**	3.2	6.6	9.7	9.4	1.8	21.4	**49.3**
2015 Dec	66	**37.4**	4.3	1.5	3.7	24.0	12.9	16.1	**68.8**
2016 Feb	73	**35.8**	3.1	4.0	4.1	11.1	5.1	36.9	**63.4**
2016 Dec	84	**59.2**	7.9	4.1	4.5	15.4	5.0	3.9	**57.3**
2017 Feb	80	**70.4**	8.6	4.7	2.9	6.3	3.1	2.8	**48.8**

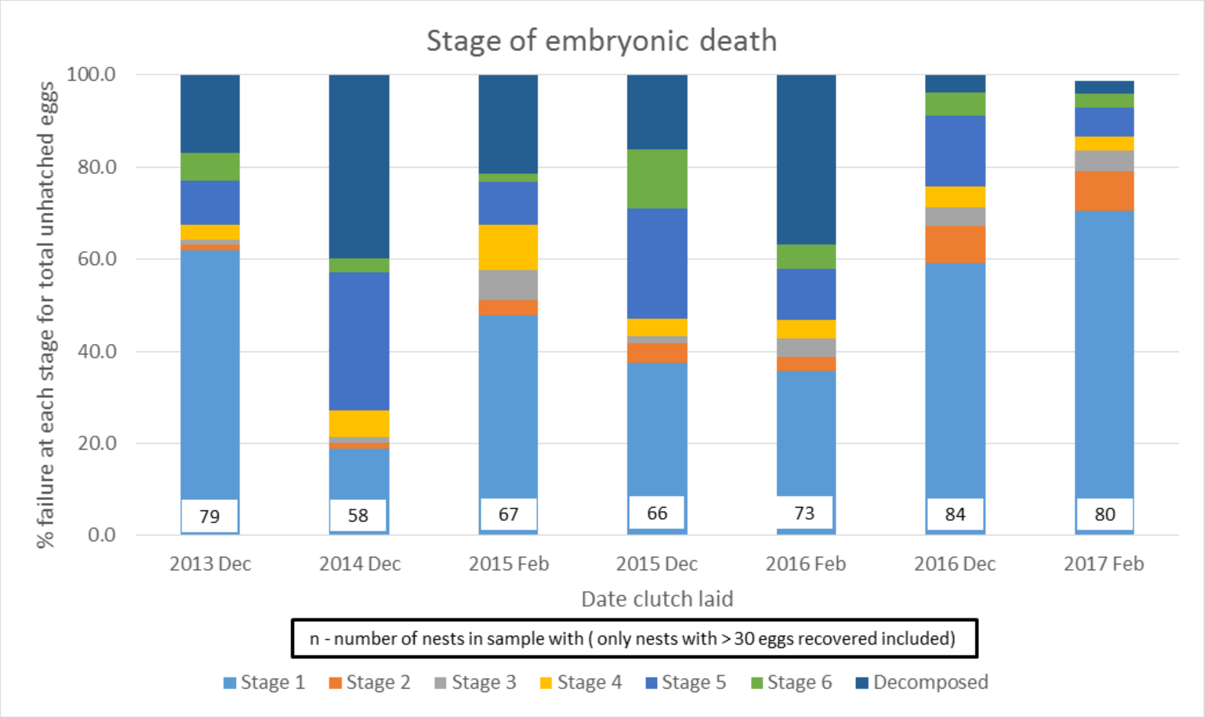

Figure 15. *Stage of embryonic death for clutches marked and excavated from 2013-2017*

5. HATCHLING PRODUCTION

Methods

Hatchling count surveys were conducted during Feb 2-4 and Apr 3-6, 2017 in the same survey sectors as the nesting success surveys of Nov/Dec, 2016 and Jan/Feb, 2017 at both Raine Island and Moulter Cay.

Moulter Cay hatchling counts were restricted by tides and weather and were conducted only on one night from 1800 – 2115 hrs.

Pitfall traps were prepared or repaired prior to 1800hrs. Traps consisted of trenches with vertical sided walls, 50m long and approximately 15cm wide and 15cm deep (Figs. 16a & b). Deep pitfall holes with vertical walls and approximately 60cm in depth and diameter were constructed at each end and at the centre point of the trench. Traps were monitored by 2 people throughout the night to record and release hatchlings and repair the trench after nesting turtle crossings. Adult turtles were encouraged to nest elsewhere as they emerged from the water, to minimise trench repair efforts.

Hatchlings were collected, recorded and released approximately every 15 minutes with total hatchling counts recorded for each hour from 1800 – 3000 hours each survey night.

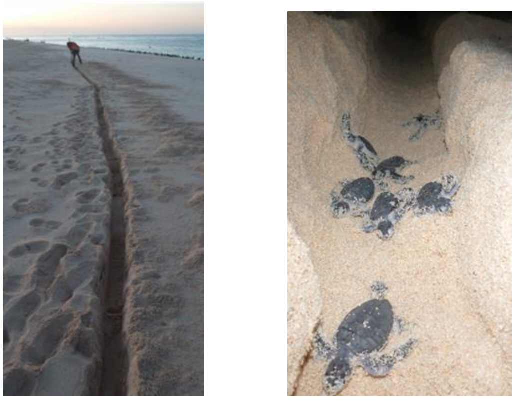

***Figure 16a & b**. 100m pitfall traps*

The measured number of eggs laid in December can be translated to an expected number of hatchlings 56 days later.

Hatchling production is the percentage of eggs which actually produce hatchlings when the same sector is surveyed at least 56 days after eggs were laid.

% hatchling production = total hatchlings per 50m sector / total eggs laid per 50m sector on a ‘per night’ metric

A separate calculation can be made to estimate the maximum number of eggs which could be expected to be laid in perfect circumstances by the nesting population at Raine Island. This maximum expected reproductive output for 50m study sectors given a known number of nesting females present at Raine Island (Peterson estimate) can be calculated.

This is based on a number of assumptions:

- Assumptions already covered in Peterson estimate methods
- A constant total number of nesting female turtles present at Raine during the nesting period of interest
- Each individual female turtle nests successfully every 12th night
- Individuals turtles lay repeated clutches during the nesting period of interest
- There is an equal chance of a turtle coming ashore and nesting anywhere around the 1800m island shoreline.

There is little doubt that these assumptions are not met at Raine Island with low nesting success and repeated nesting attempts most likely increasing time between successful nesting efforts and reducing numbers of clutches laid per season. Rocky shoreline in places limits nesting beach access and results in an uneven nesting distribution around the island.

This is however a best-case scenario reproductive output, which does come close to the reproductive success occurring at rookeries with lower nesting densities. It is however a valuable estimate to compare with what is actually being observed at Raine Island.

maximum eggs per night = Total breeding females x 104.3 eggs x 1/12 night

The total Raine Island shoreline distance is approximately 1800m therefore:

maximum reproductive output per 50m per night = maximum eggs per night x 50m/1800m

and:

% maximum reproductive output = (total hatchlings /50m/night) / (maximum reproductive output / 50m / night)

Results

5a. Hatchling counts 2016-17 season

Hatchlings counts for 2016-17 were the highest since hatchlings counts were initiated using standard methods in 2012-13. The exception to this was in April 2014 when a large percentage of hatchling production was from above the cliff area resulting from a large nesting season and high levels of nesting in this area (Table 17 and Fig. 17).

The comparison of hatchling counts for the re-profiled area with un-modified areas shows similar levels of hatchling production in the Dec – Feb period. It is very different in Feb – April incubation period with approximately double the hatchling numbers counted in April in the re-profiled area (Table 17 and Fig. 17). Of note is the low hatchling production in Sector D in both February 2016 and 2017.

Table 17. *Raine Island mean hatchling counts for 100m survey sectors for 2014-15 to 2016-17 seasons (Sectors A, B and re-profiled C)*

	2015 - Jan	2015 - Feb	2016 - Feb	2016 - April	2017 - Feb	2017 - April
Sector A2	1012	1490	2089	620.0	2650	2695
S.E	± 176.9	± 100.3	± 503.9	± 95.3	± 129.8	± 504.1
Sector B	529	1192	2206	1077.0	2922	1995
S.E	± 163.5	± 215.2	± 508.5	± 223.7	± 310.5	± 253.6
Re-profiled	1342	2676	2369	1147.0	2832	3943
S.E	± 195.7	± 283.8	± 741	± 67.4	± 164.8	± 640.2
Sector D			310			775
S.E						± 0.0

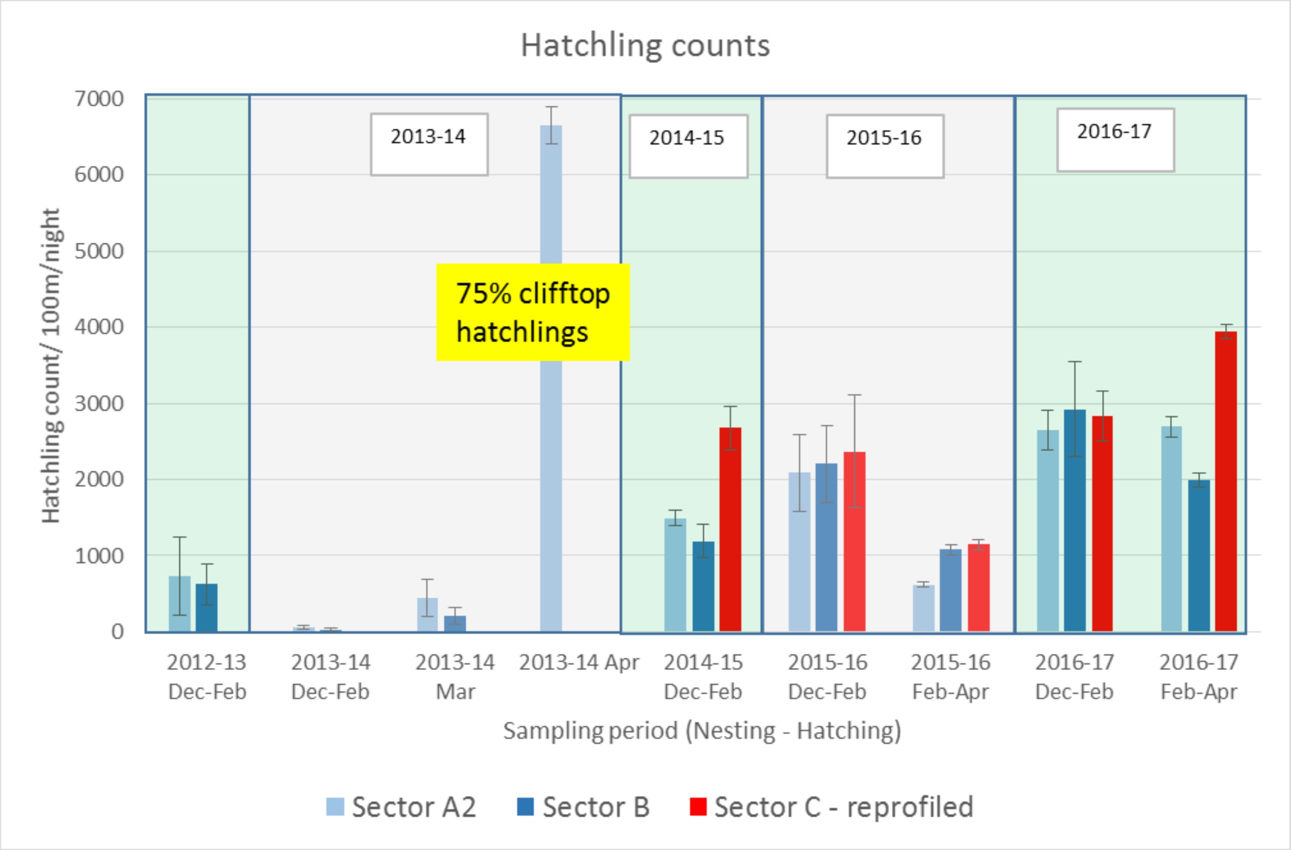

Figure 17. *Mean hatchling counts for full night sampling in Sectors A & B and the re-profiled Sector C for 2012-13 to 2016-17 seasons. Standard error bars are shown. Note that in 2013-14 April period 75% of hatchlings came from above the clifftop area in the single sectorA2 surveyed.*

5b. Reproductive success for the 2016-17 season

Reproductive success is defined in this report and in general within the Raine Island Recovery Project as follows:

Reproductive success = (Total recorded hatchlings per night / total expected hatchlings per night) x 100%

Total recorded hatchlings per night = mean hatchlings recorded per 100m survey sector x 18 (for estimation of total island hatchlings per night).

This calculation uses a berm distance of 1800m instead of the actual 2000m distance around the island because some areas are less productive than survey sectors due to beachrock restrictions to nesting and therefore hatchling production.

Total expected hatchlings per night is calculated from the measured nesting turtle population as if there is

- 100% nesting success, with nesting once every 12 nights
- 104 eggs laid per clutch
- an average of five clutches laid per individual during the season
- 100% emergence success
- No beach predation

Reproductive success, measured by hatchlings per clutch laid, did not show an increase in the re-profiled sector when compared to controls in any of the three seasons to date season (Table 18 and Fig. 18).

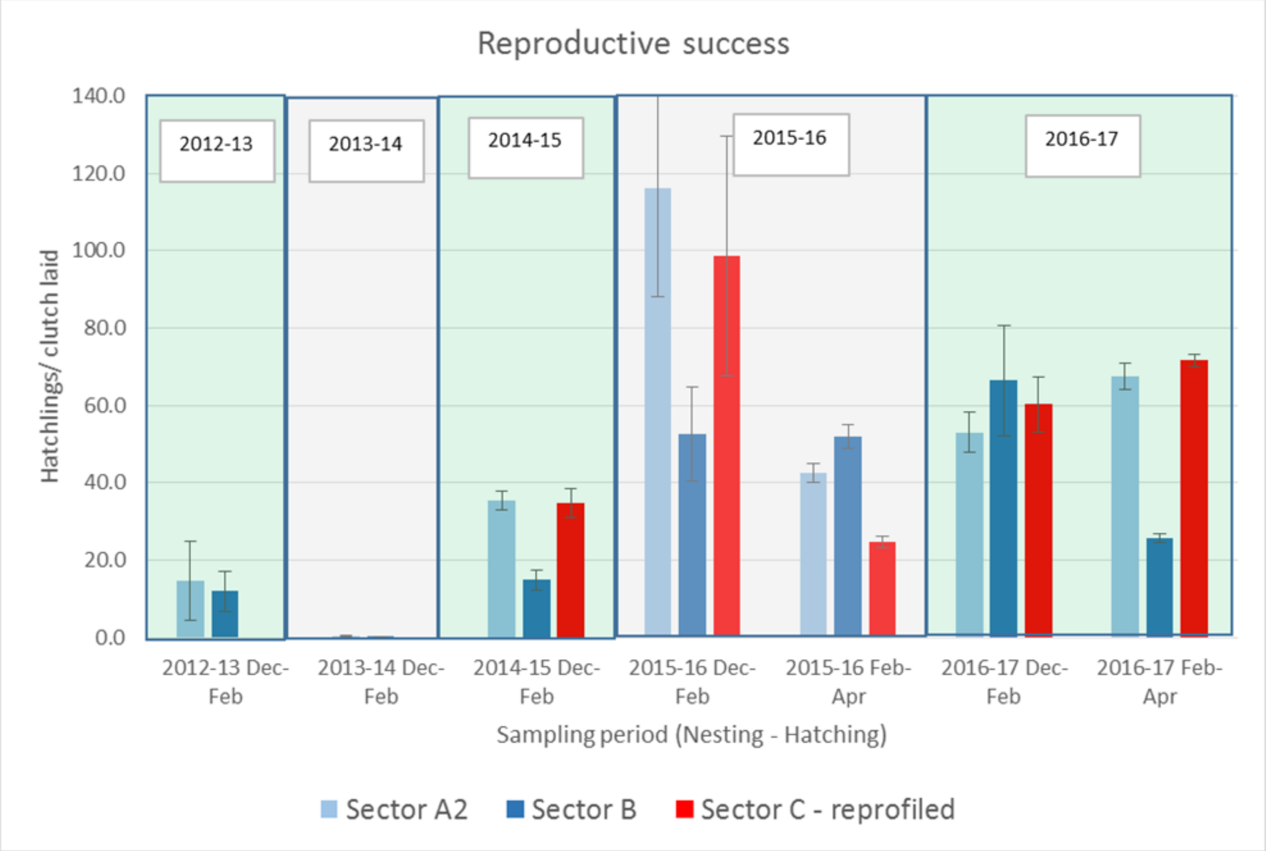

Figure 18. *Hatchlings produced per clutch laid for survey sectors. Standard error bars shown*

Table 18. *Summary of hatchling production data and reproductive success for 2012-13 to 2016-17 seasons*

Sampling period	Sector A2					Sector B					Sector C - reprofiled				
	**Mean total clutches / night**	**Mean total hatchlings / night**	**S.E**	**Hatchlings per clutch laid**	S.E	**Mean total clutches / night**	**Mean total hatchlings / night**	**S.E**	**Hatchlings per clutch laid**	S.E	**Mean total clutches / night**	**Mean total hatchlings / night**	**S.E**	**Hatchlings per clutch laid**	S.E
2012-13 Dec-Feb	50	731	513.2	**14.6**	10.3	52	626	270.2	**12.0**	5.2					
2013-14 Dec-Feb	132	52	24.0	**0.4**	0.2	200	30	20.0	**0.2**	0.1					
2013-14 Mar		442	249.4				202	110.9							
2013-14 Apr		6656	251.7												
2014-15 Dec-Feb	42	1490	100.3	**35.5**	2.4	80	1192	215.2	**14.9**	2.7	77	2676	284.1	**34.8**	3.7
2015-16 Dec-Feb	18	2089	503.9	**116.1**	28.0	42	2206	508.5	**52.5**	12.1	24	2369	741.0	**98.7**	30.9
2015-16 Feb-Apr	16	620	35.5	**42.6**	2.4	21	1077	64.5	**52.0**	3.1	44	1148	67.5	**24.7**	1.5
2016-17 Dec-Feb	50	2651	259.8	**53.0**	5.2	44	2921	621.0	**66.4**	14.1	47	2831	329.7	**60.2**	7.0
2016-17 Feb-Apr	40	2695	135.8	**67.4**	3.4	78	1995	89.6	**25.6**	1.1	55	3943	88.6	**71.7**	1.6

6. ECOLOGY OF RAINE ISLAND BEACH

6a. Tidal inundation of the nesting beach

Methods:

Troll 500 water level loggers were placed in 90mm galvanised pipe which was sited below the sand surface with the base at the lowest elevation feasible. Four logger transects were installed (T2, T3, T4 & T5) with loggers located from the berm to back swale in the nesting beach (Figure 19). Loggers recorded height of water above the sensor at 15 minute intervals during 2012-present. Reliability of the loggers' performance and durability has been an issue. Data has been reduced to that which is reliable and accurate for use in analysis.

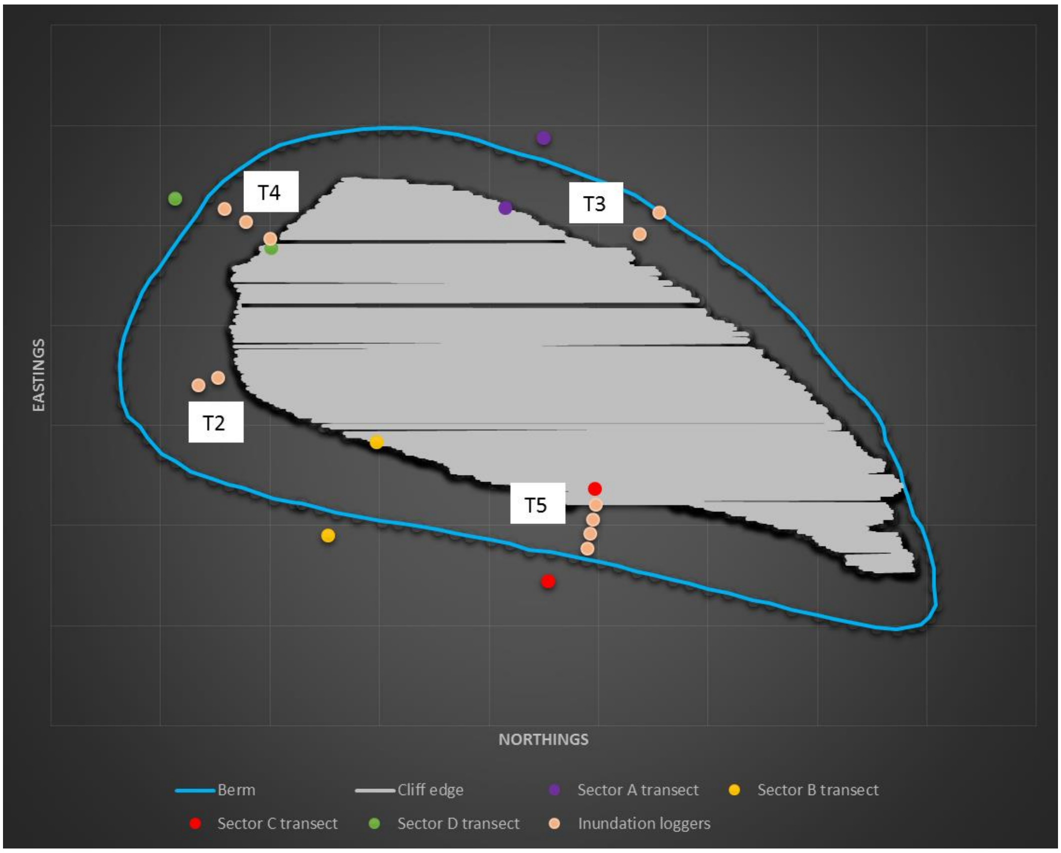

Figure 19. *Location of cross nesting beach transect profiles and inundation loggers. Logger transects are marked.*

Wells were dug around the nesting beach and water level measured at highest astronomical tide (HAT) with dGPS to (Fig. 25):

- investigate whether levels were consistent around the entire nesting beach
- ground truth water level loggers

Wells were dug across the nesting beach from berm to cliff in Sector D and sampled at HAT to (Fig. 22):

- determine height and time lag peak water level differences from berm to cliff across the nesting beach
- estimate length of inundation time for nests below inundation level.
- further ground truth water level loggers

Weather station data was recorded as described later in this document.

Inundation - influence by tide, rainfall and weather

- Inundation levels follow tide levels very closely

- The berm is has raised inundation levels corresponding to high winds/overtopping/surge events - high WNW winds observed during fieldwork in February 2017 seem to influence the T4 (NW) berm (T4.2) and swale location (T4.3) but not in T5 (SSE) ot T2 (SW) swale locations (T5.4, T2.3). (Fig. 20 a &,b)
- Inundation across the nesting beach is affected by major rainfall events but is more pronounced in the swale and back swale (Fig 20b. and Fig 21 a,b, c)

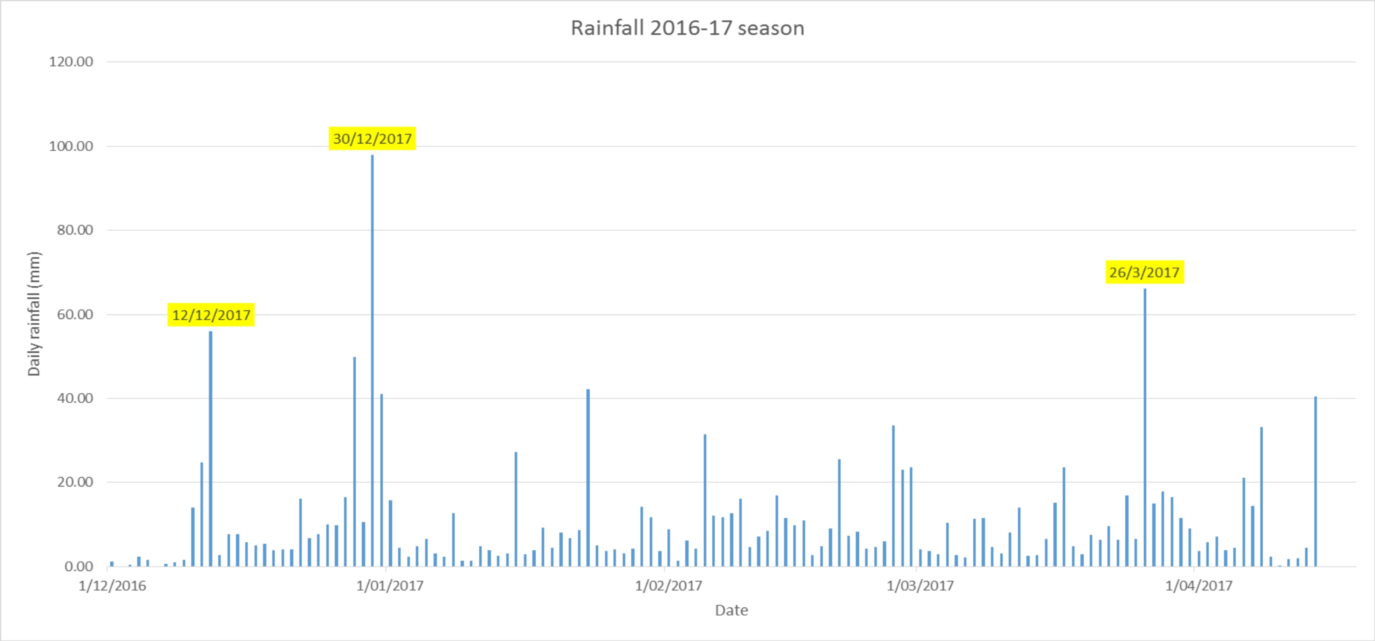

Figure 20a. *Rainfall records from Raine Island vaisala weather station for 2016-17 season. Labels highlight peak rainfall events.*

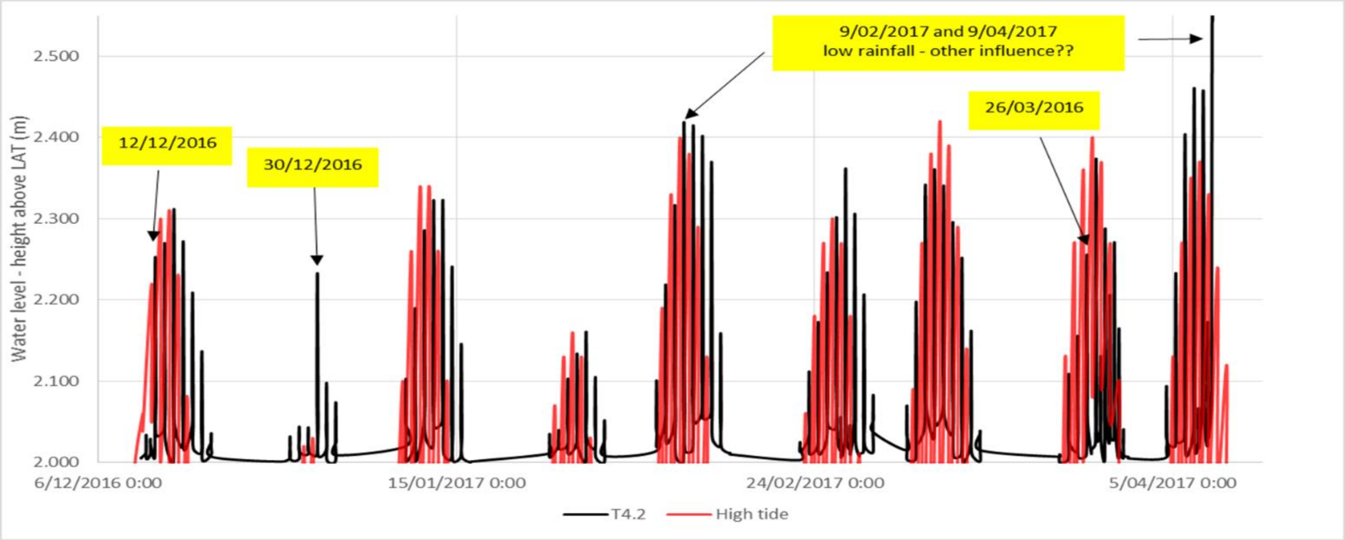

Figure 20b. *Troll 500 water level sensor records for berm logger T4.2 in Transect 4 (NW section of the nesting beach for the 2016-17 season. Labels highlight inundation levels at times of peak rainfall events and also where inundation does not follow tide levels and there is no significant rainfall.*

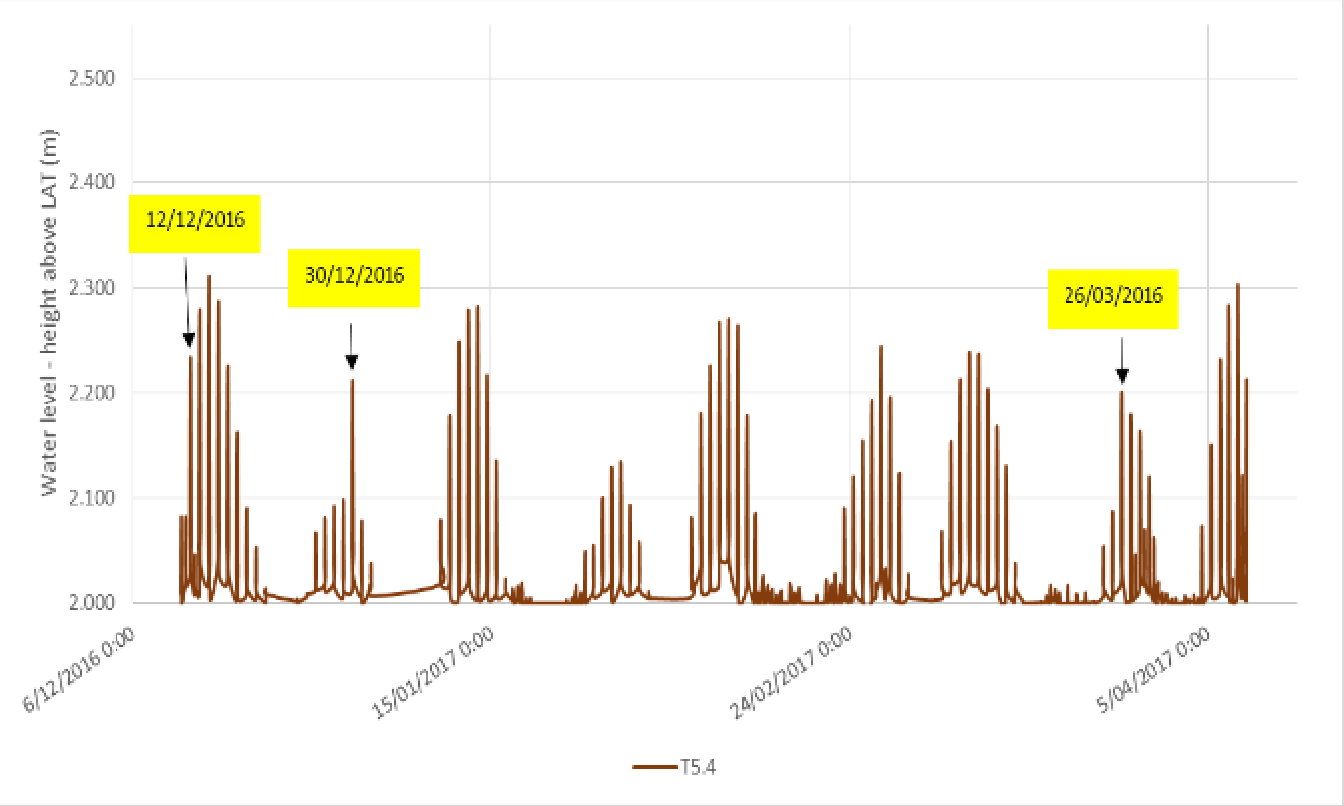

Figure 21a. *Troll 500 water level sensor records for swale logger T5.5 in Transect 5 (Sth) section of the nesting beach for the 2016-17 season. Labels highlight inundation levels at times of peak rainfall events.*

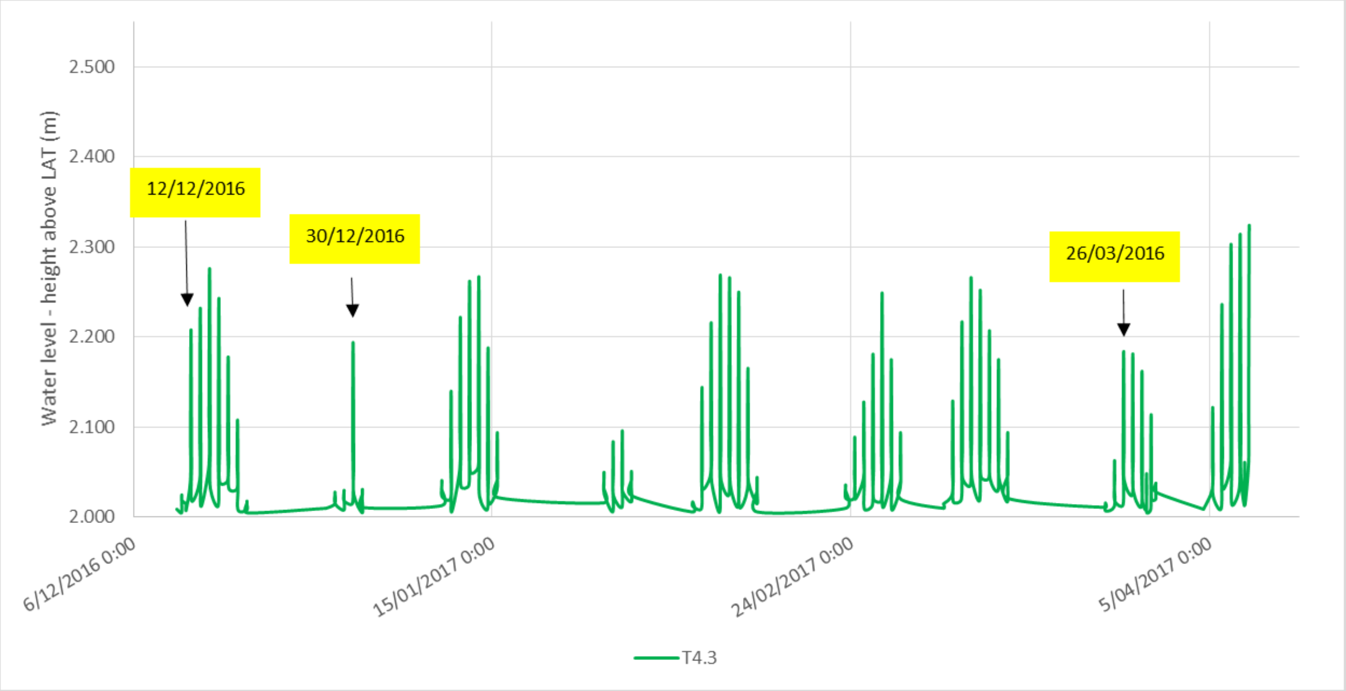

Figure 21b. *Troll 500 water level sensor records for swale logger T4.3 in Transect 4 (NW) section of the nesting beach for the 2016-17 season. Labels highlight inundation levels at times of peak rainfall events.*

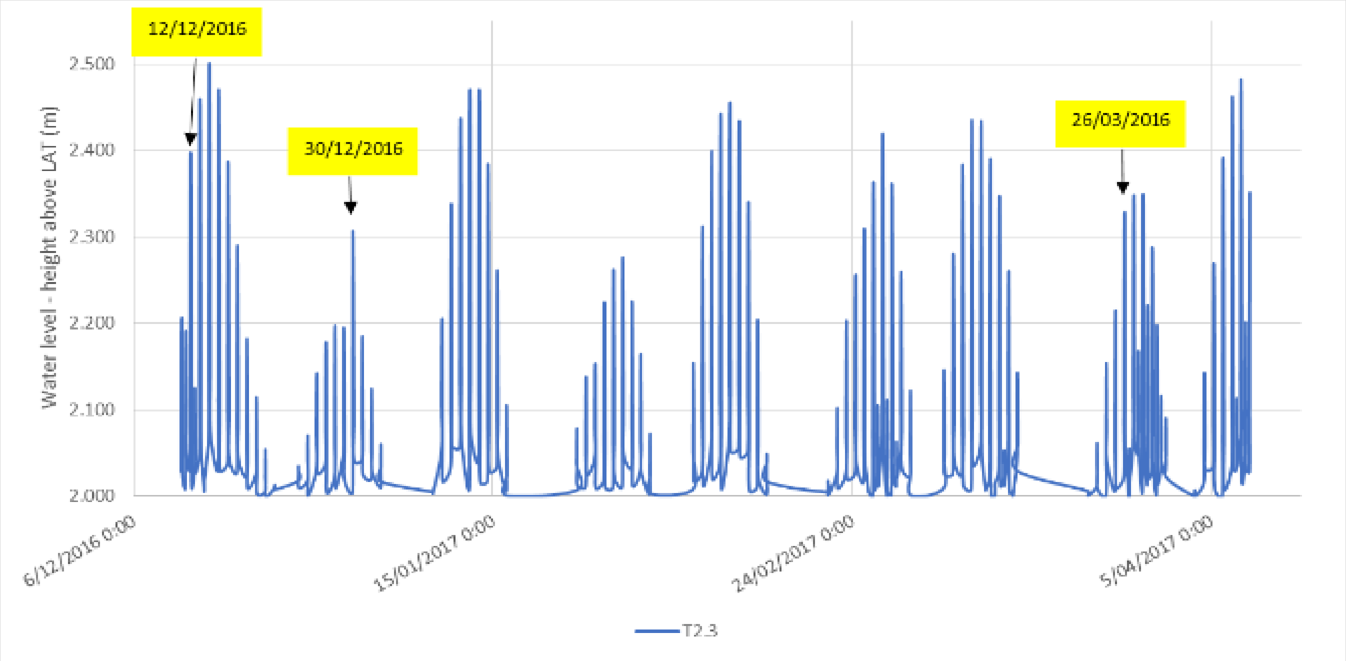

Figure 21c. *Troll 500 water level sensor records for swale logger T2.3 in Transect 4 (SW) section of the nesting beach for the 2016-17 season. Labels highlight inundation levels at times of peak rainfall events.*

Direct inundation measurements of sampling wells at peak tide levels

- Water levels are consistent throughout the nesting beach and increase from cliff towards berm. (Fig.23, Fig. 25)
- Readings within the south western section of the nesting beach swale and back swale are approximately 0.2m higher than elsewhere. These correspond to inundation logger records and have been taken into account in analysis of inundation levels and hatching success calculations relating to this.
- Peak water level is approximately 0.45m higher in the berm than the back swale. A lag period of approximately one hour occurs between peak berm and peak back swale water level. (Fig.23)
- Minimum duration of inundation for eggs laid 0.1m below peak inundation height 2 hrs 40 minutes at peak inundation periods. (Fig 24.)

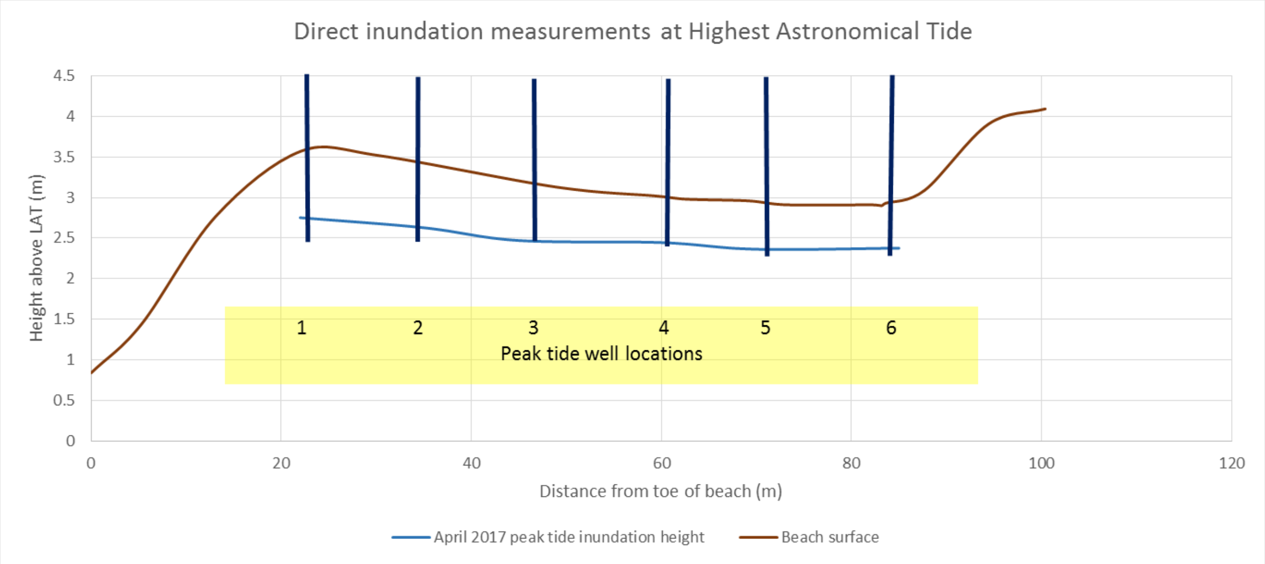

Figure 22. *Location of HAT sampling wells across Sector D (Transect 4) NW nesting beach in April 2017*

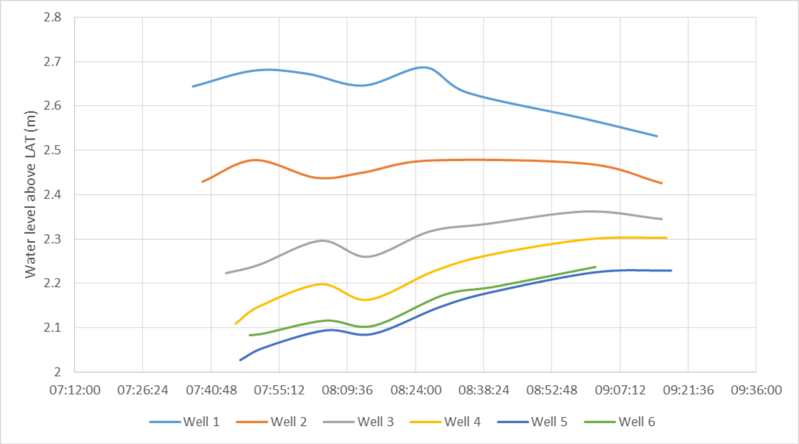

Figure 23. *Timing and height of water level in HAT sampling wells in sector D*

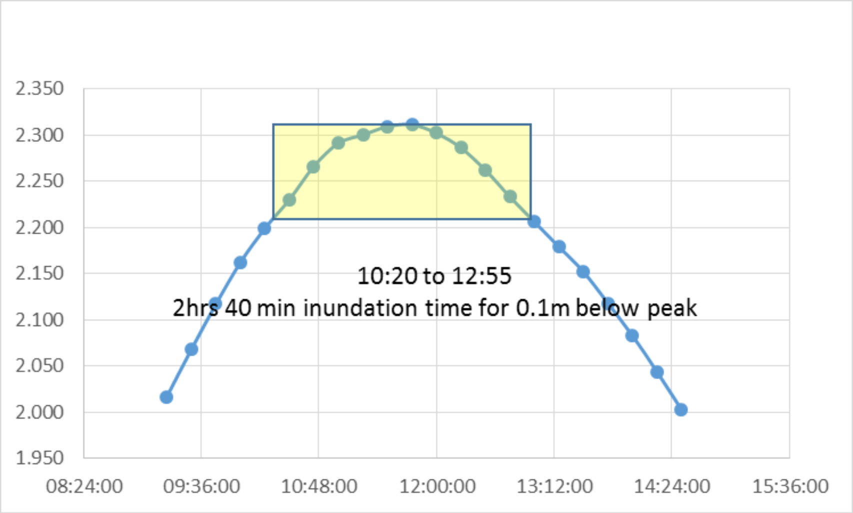

Figure 24. *Inundation timing - minimum inundation time for eggs 0.1m below peak inundation height 2 hrs 40 minutes*

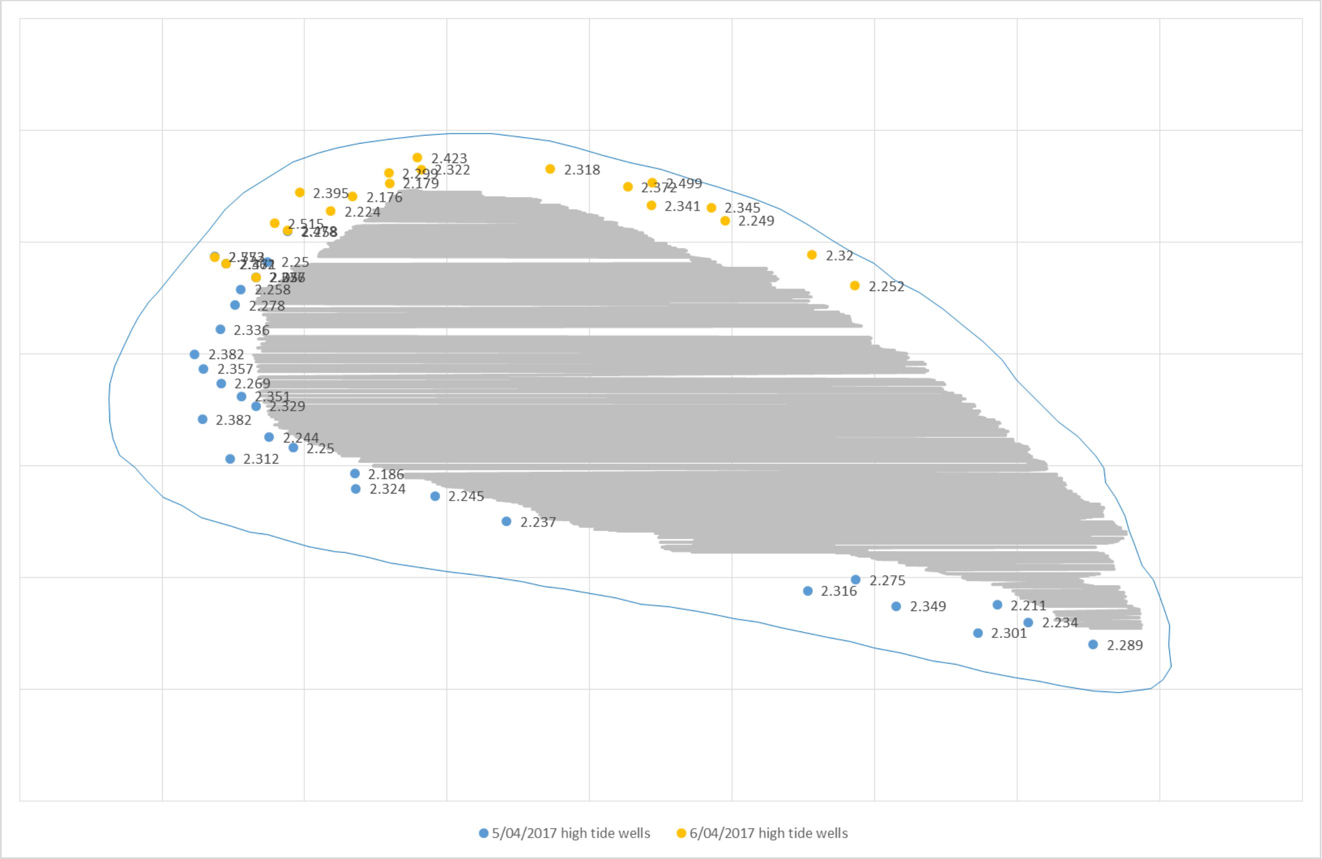

***Figure 25**. Direct measure of water level throughout the nesting beach on highest astronomical tide (HAT) 5-6 April 2017. Well measurement points are labelled with water level height (m above LAT)*

Profiles of beach surface and peak inundation level with the depth marked at which nests would on average be laid provide a good indication of the areas of impact due to inundation. (Fig. 26 a, b, c & d and Fig 27). This indicates that most of the swale and back swale area and some of the back berm is inundated at peak tides to above nest level. The berm in the north-western Sector D is vulnerable to inundation and the re-profiled area is well above inundation level throughout its entire nesting beach area (Fig. 27).

- Sector A2 has a major area of the back berm, swale and back swale where inundation level is above nest level.
- Sector B is affected by inundation but to a lesser extent
- Sector, the re-profiled sector is above inundation in all areas
- Sector D has most areas affected by inundation at nest level

Figure 26a.

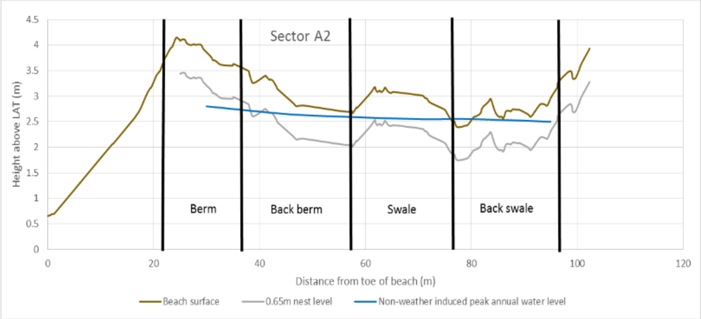

Figure 26b

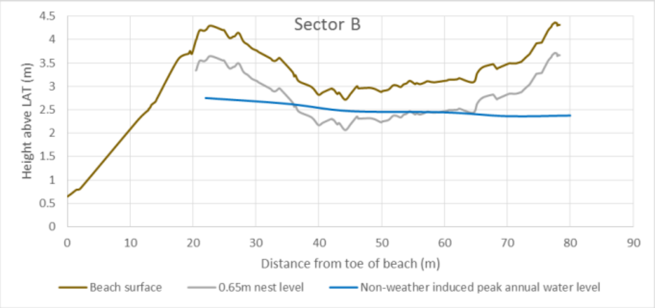

Figure 26c.

Figure 26d

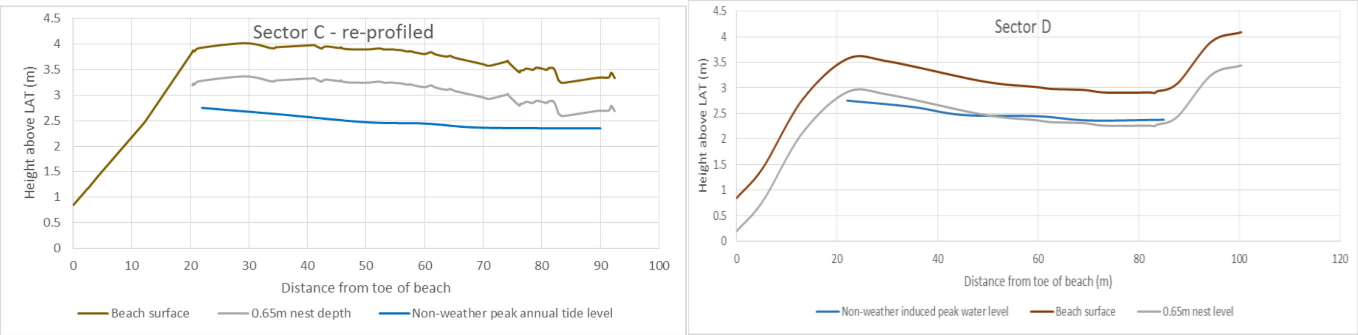

Figures 26 (a, b, c & d). *Cross beach profiles for survey sectors A, B, C re-profiled and D with inundation level*

Beach surface profile is shown (brown line) with a grey line representing the average level for the middle section of green turtle clutches laid 0.65m below the beach surface. The blue line represents the water level at peak tidal inundation time.

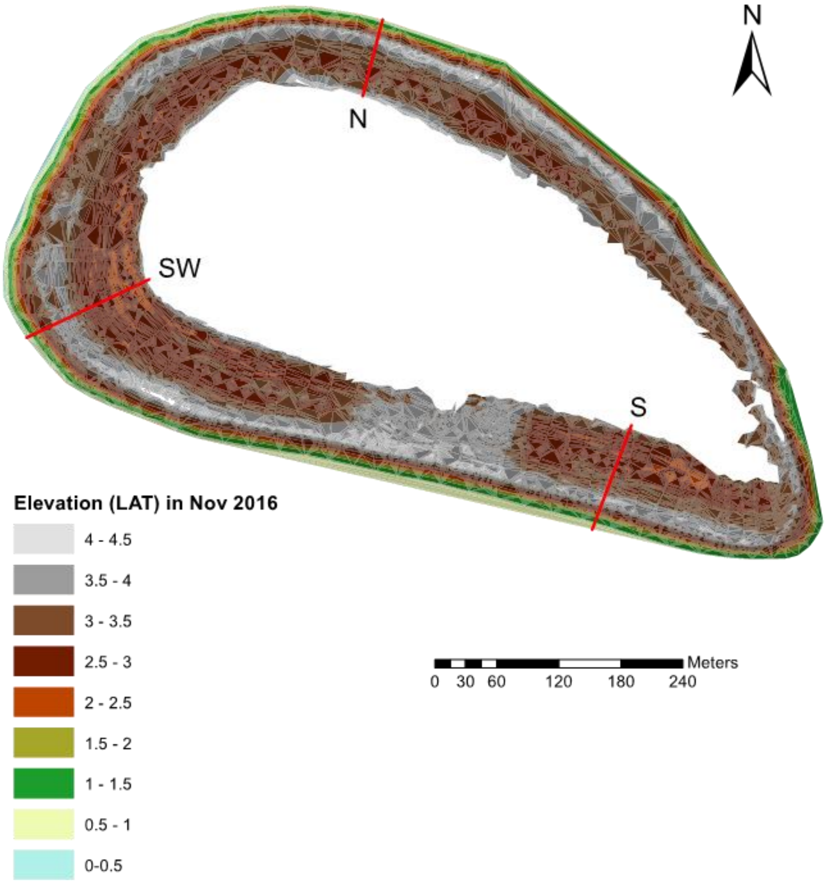

Figure 27. *Nesting beach surface height above LAT (m).*

Note that grey areas are above inundation level at all times while brown areas are generally below inundation level at peak spring tides.

6b. Sand temperature at nest depth

Method:

Temperature loggers (TinyTag Plus2) were fixed to timber stakes driven beneath the nesting beach surface with loggers located 0.65m below the surface to represent median nest depth and centre of clutch for green turtles. Three

transects were chosen to match with survey areas for nesting success and hatchling production surveys. Loggers were positioned at four locations across the nesting beach, on the berm, back berm, swale and back swale (Fig. 28).

Results:

Temperatures at nest depth were generally higher in the swale and back swale which may correlate with sand colour as these areas have higher organic levels within the sand content and darker colour.

Temperatures during the peak incubation period of clutches during the breeding season were generally above the sex determination threshold temperature of 29.3°C (Ian Bell, unpublished data). This indicates that most hatchlings produced would be females however there are spikes of lower temperatures which occur due to major rainfall events and which last for multiple days (Fig. 29). Such a lowering of temperature during the sex determination period in the middle stages of embryonic development would most likely result in production of male hatchlings.

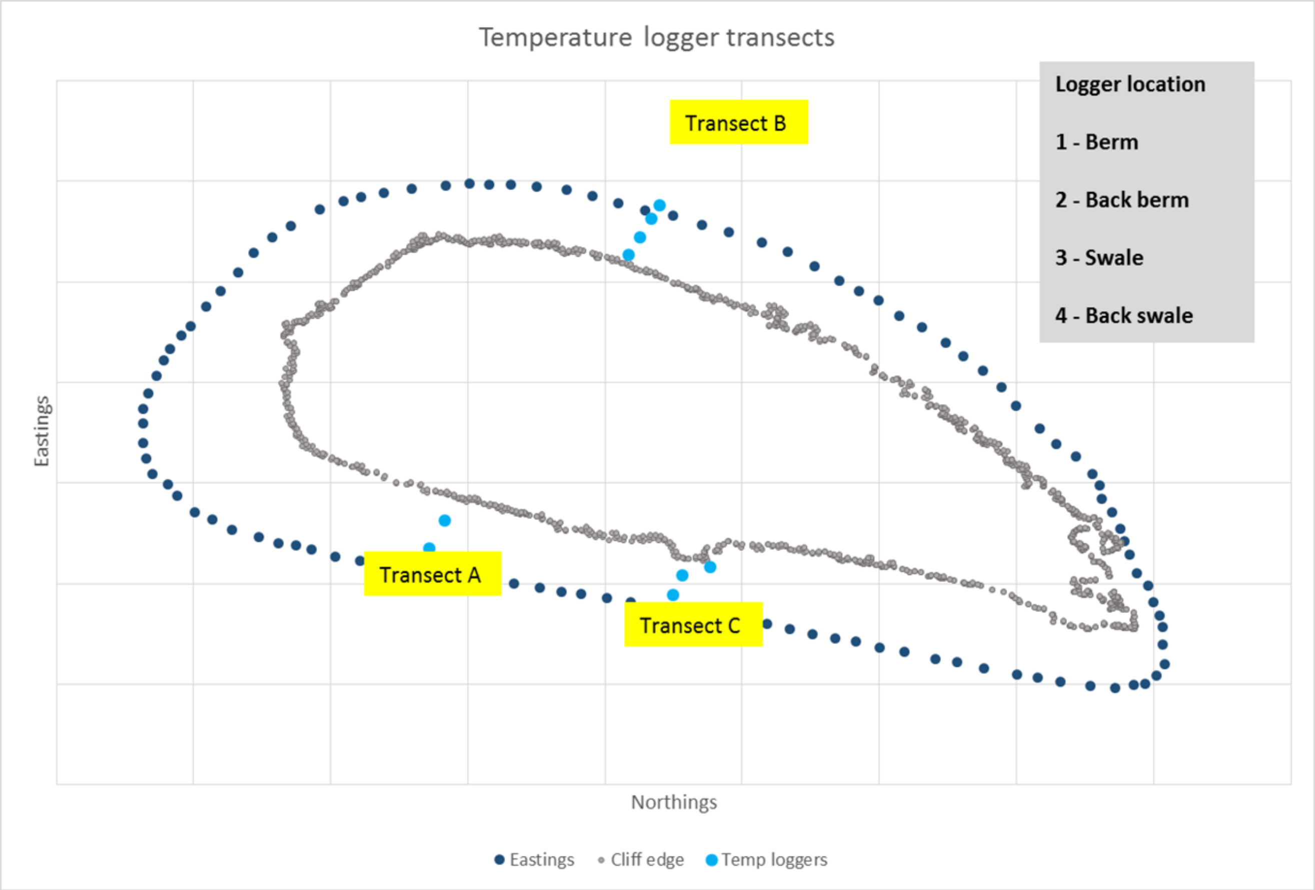

Figure 28. *Location of nest depth temperature loggers*

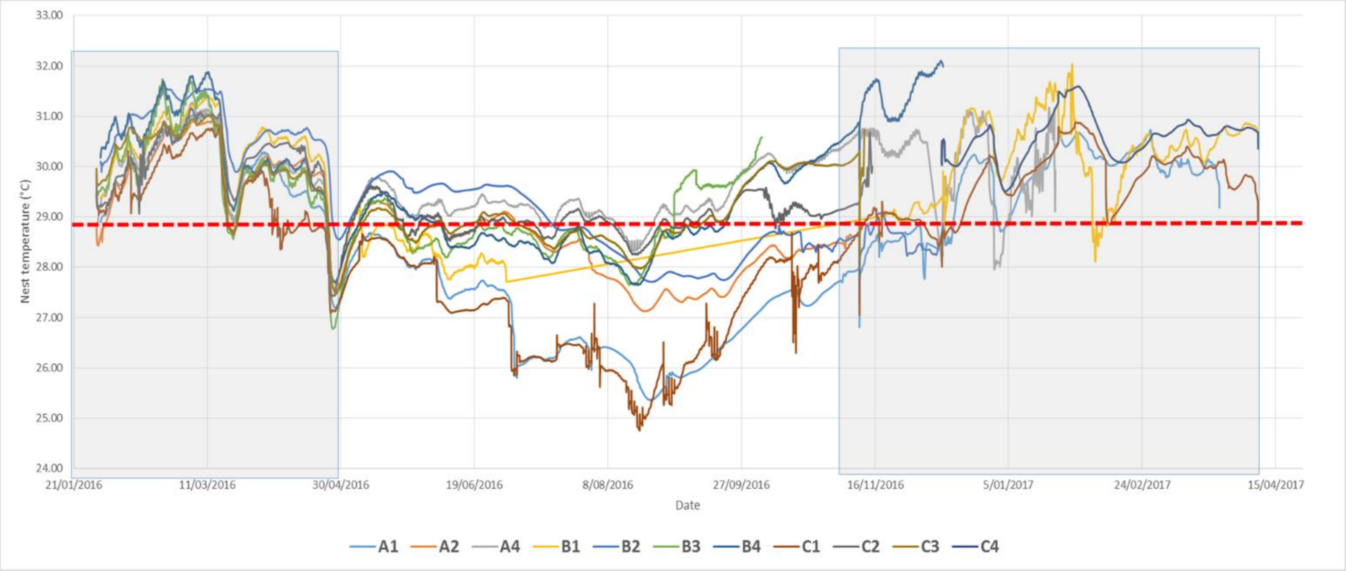

Figure 29. *Temperature at nest depth (0.65m below beach surface) from Jan 2016 to April 2017 at berm, back berm, swale and back swale locations in three transects across Raine Island nesting beach (Fig 28)*

6c. Stability of the re-profiled area

A 150m x 100m sector of the Raine Island nesting beach from berm to cliff was raised above tidal inundation level by moving sand from the berm face to rearward lower lying areas in September-October 2014.

Results

The re-profiled sector sand profile is still intact following the 2016-17 nesting season. Vegetation regrowth prior to this nesting season was limited and patchy.

Re-profiled area models from start to most recent to be inserted

7. TOPOGRAPHIC SURVEY

7a. dGPS

Methods

Trimble dGPS units were used by a qualified surveyor on each Raine Island field trip to:

- survey the entire beach nesting area and cliff edges
- conduct fine scale surveys of the re-profiled area of the nesting beach and control survey sectors
- mark and relocate 2016-17 season turtle nests
- mark key infrastructure features including fencing, remote sensing structures, water level measurement points and tidal elevations, red-tailed tropicbird nests and seabird monitoring locations.

Results

Data was analysed and mapped in ArcGIS for digital elevation models, including nesting beach inundation and used in calculations and mapping of turtle nests for emergence success, red tailed tropic bird nests, infrastructure locations and turtle mortality.

7b. Drone survey

Methods

An Inspire quadcopter (Biopixel) was used to

- topographically map Raine Island to 2cm resolution
- conduct seabird census surveys (simulatenously with topographic mapping)
- conduct surveys for painted turtle in-water counts for nesting population census in parallel with surface observer and GoPro methods
- conduct dawn nesting beach turtle surveys

Ground control markers were placed around the island, location recorded by dGPS and used during analysis to rectify drone imagery.

Flights were conducted using the following standard operating procedures developed following research to assess nesting seabird disturbance and set minimum disturbance methods.

Drone Standard Operating Procedures

- drone to take off and land vertically on the beach berm adjacent to Raine base station or from vessel as distant from nesting seabirds as possible
- drone survey altitude to be at a minimum of 60m
- surveys not to be conducted at times when seabirds are undertaking daily migratory departure from or return to the island
- surveys to be stopped if significant disturbance is observed

Results

3D topographic images were produced from drone image data by Rob Beamann (JCU) and Dan Breen (AUT). Data was used in calculations for inundation, nest mapping and nesting seabird surveys.

Painted turtle counts were conducted and analysed as described in relevant section of this report.

Bathymetric mapping of the mooring and beach landing areas

Method:

The bathymetry of the western end of Raine Reef, in the vicinity of the mooring, was mapped using a singlebeam echosounder (SonarMite-MilSpec) with a 200-kHz transducer attached to a survey pole with a Trimble R8 GPS receiver. The survey pole was mounted to the transom of a small vessel. Depths were recorded directly to a Trimble TSC3 survey controller and corrected in real time to elevations relative to lowest astronomical tide using the permanent base station installed on Raine Island. Point data were generally recorded every 5 seconds and in an approximate criss-cross pattern covering a region of approximately 300m by 100m. Point data were used to create a digital elevation model and contour map. Accuracy is considered ±0.025m RMS.

Results:

A bathymetry map (Fig.30) and bathymetry transect (Fig.31) of the mooring area and approach to the nesting beach were produced. This will provide information to ensure minimum impact of any future barge access requirements to the Raine Island beach and adjacent reef shallows.

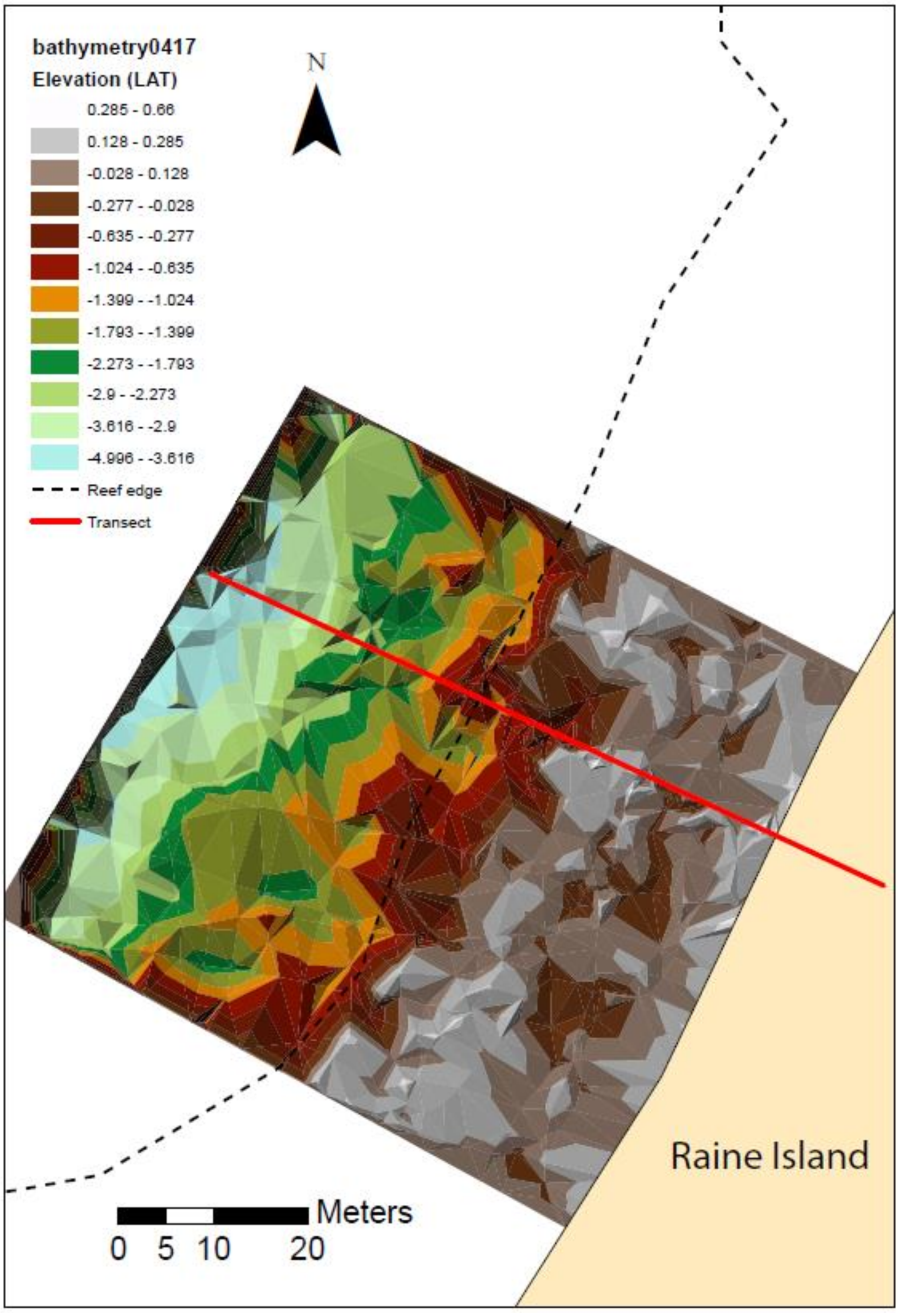

Figure 30. *Bathymetric profile of the morring area and approaches to Raine Island beach*

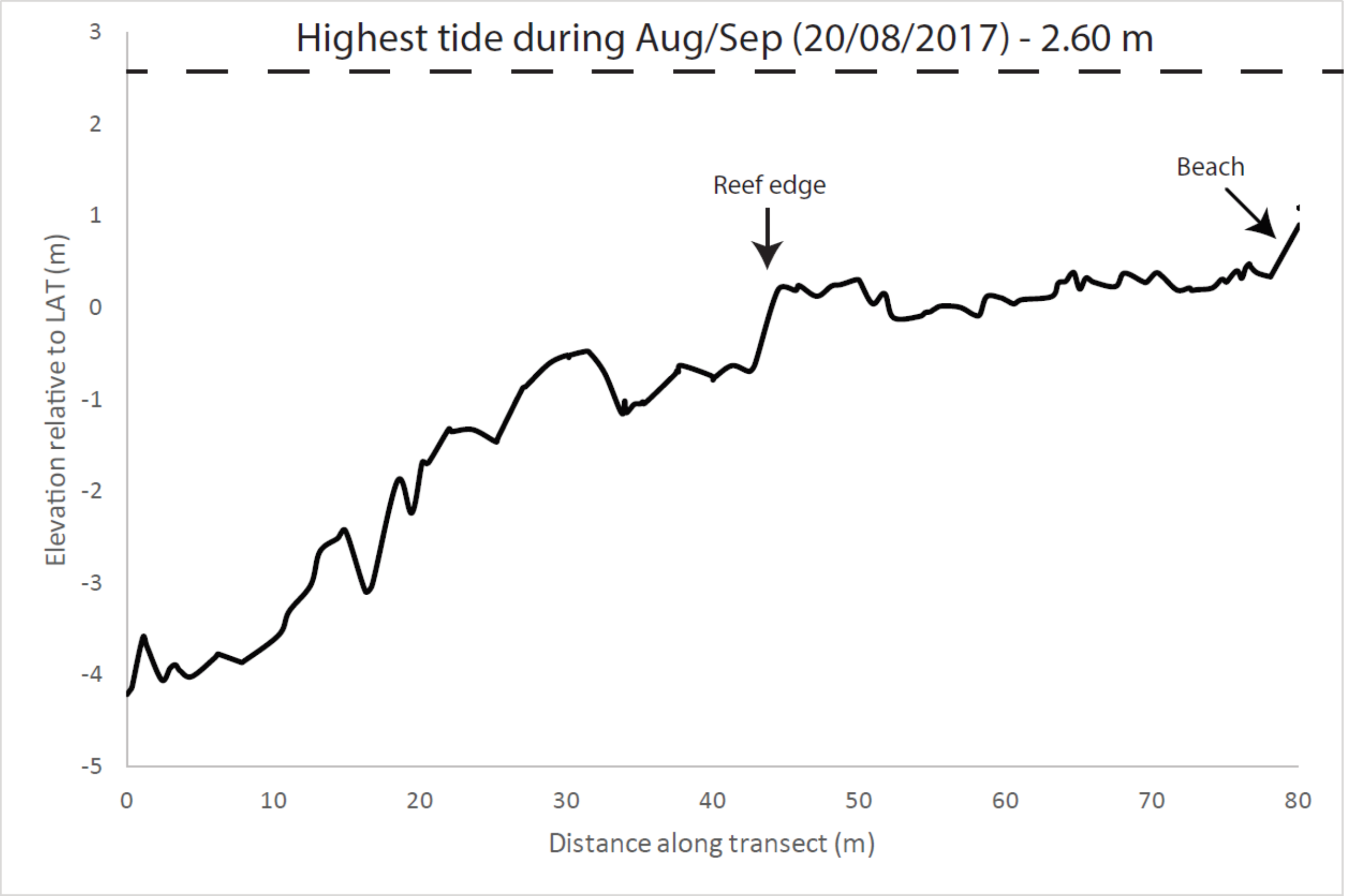

Figure 31. *Bathymetric transect of the approach to Raine Island beach from the mooring area*

8. Satellite telemetry of nesting mature female green turtles at Raine Island 2016-17

Summary adapted from: Shimada et al 2017. Assessment of the potential for Fastloc GPS satellite telemetry to quantify the breeding success, habitat use and effectiveness of habit restoration for green turtles nesting at Raine Island: a report on use of satellite telemetry at Raine Island, 2015 and 2016 breeding seasons.

The complete report for this project is presented in the appendix.

Introduction:

Investigation of green turtles based on tagging studies have been long been used to describe nesting behaviour and estimate population size of the Raine Island nesting turtles using data collected by surveys on the island and in the waters around the island. It was not possible with these studies to quantify key demographic parameters such as the number of clutches laid per female per breeding season and adult survivorship. In addition, a key assumption of the population estimation methods is that the turtles remain in the vicinity of the island during the nesting season. However, flipper tag recovery records indicate that some turtles may not stay close to Raine Island, instead spending some time elsewhere between successive nesting occasions where they can't be sampled using the existing techniques. The potential departure from the area adjacent to Raine Island indicates that the assumption for the current methods for population estimation may be violated, and subsequently the estimates may not be reliable. Clearly the movements of turtles within the inter-nesting period need to be taken into account to accurately estimate the population size of green turtles nesting at Raine Island.

Until recent years, no methods had been available for tracking marine turtles at sea with sufficient accuracy to adequately resolve their positions within the scale of Raine Island beach and its associated reef and the surrounding in-water survey area. A location resolution of <100 m is required to achieve this. High-resolution tracking is now possible using the new Fastloc GPS technology (FGPS).

In the present study, we used a small number of FGPS tags to demonstrate their capacity for collecting accurate tracking data from female green turtles nesting at Raine Island and providing results not available from previous studies. These tags have the capacity to record the timing and location of a turtles nesting activities on the island and hence provide a minimum estimate of the number of clutches laid within a nesting season by individual females and a measure of their nesting success (proportion of visits to the island that result in egg laying). The

satellite telemetry data can be analysed to estimate the probability of a turtle's presence and absence within the in-water survey area where mark-recapture studies occur for estimation of population size. These availability estimates have the potential to improve the estimation of turtle population size within the inter-nesting habitat at Raine Island, and will thus allow for more accurate estimate of the past and present population sizes within the nGBR green turtle stock. Once the tagged turtles cease nesting for the season, the FGPS data also allows for precise definition of migratory pathways, locations of their foraging habitats and the geographical extent of these foraging areas.

This report primarily focus on the habitat use of female green turtles at the Raine Island region during their inter-nesting periods and provides biological insights inferred from satellite tracking data, which have never been recorded for the Raine Island turtles using other conventional methods.

Methods:

Adult female green turtles were captured during their nesting attempts at Raine Island in December 2015 (n = 2) and November 2016 (n = 3). Given that the peak nesting density occurs during December and January at Raine Island (Limpus et al. 2003), the turtles captured in 2016 were presumed to be near the commencement of hat nesting season, and those in 2015 were likely at the middle of their nesting season. Each turtle had been sighted at Raine Island at least once during the previous surveys (Table 19). K74859 had also been recorded once at her foraging habitat in the Coombe Reef (latitude -14.425, longitude 144.947) prior to have been recorded nesting at Raine Island. The curved carapace length (CCL) of the turtles ranged from 98.0 to 109.9 cm with a median of 103.2 cm. One turtle (I759) was missing about a half of its right hind flipper. The other turtles did not have significant flipper damage.

Each turtle was deployed with an Argos-linked Fastloc GPS tag on the carapace (Table 19). The two turtles captured in the 2015 trip were taken on a small barge, which was secured to the Reef Ranger during attachment of the tags. During the 2016 trip, the turtles (n = 3) were immobilised using the Gyuris and Limpus (1986) method and kept in a timber enclosure on the beach close to areas where they were captured while the tags were attached. The turtles were released to the nearby water on the following morning. The satellite tags were configured to obtain a GPS location every 30 minutes using the Fastloc technology.

Results:

The Raine Island Recovery Project purchased three satellite tags this season and two tags for the 2015-16 season primarily as a communication and education purposes, as agreed at last year's meetings prior to the nesting season. A naming competition for these turtles was run with students from the four primary schools in the regions representing the Wuthathi Nation and Kemer Kemer Meriam Nation (Ugar, Mer, Erub). The third turtle was named by BHP staff as partner in the Project. This tracking was led by Dr Col Limpus from Environment and Heritage Protection in collaboration with James Cook University.

Table 19. *Summary data for female green turtles deployed with a Argos-linked Fastloc GPS tags at Raine Island in December 2015 and November 2016. CCL = curved carapace length.*

Turtle ID	Argos ID	Capture history		
		Site	Month Year	CCL (cm)
I759	133766#	Raine Island	Dec 1992	109.0
		Raine Island	Dec 1997	109.5
		Raine Island	Dec 2002	109.7
		Raine Island	Nov 2016	109.9
I22154	133765	Raine Island	Nov 1993	103.0
		Raine Island	Dec 1997	103.4
		Raine Island	Dec 2001	102.1
		Raine Island	Dec 2015	103.2
K74859+	133763#	Coombe Reef	Jul 2006	104.7
		Raine Island	Dec 2006	104.8

		Raine Island	Nov 2016	105.5
		Raine Island	Nov/Dec 1984	100.5
T7159	133762#	Raine Island	Dec 1999	101.9
		Raine Island	Nov 2016	101.7
T90143	133764	Raine Island	Dec 1995	97.4
		Raine Island	Dec 2015	98.0

The satellite tags were still in operation as of 8 March 2017.

+The turtle returned to Coombe Reef following her breeding season at Raine Island, demonstrating over 10 years of site fidelity both to her nesting habitat (i.e. Raine Island) and to her foraging habitat (i.e. Coombe Reef).

Data has provided valuable information relating to nesting effort, clutches laid and migration patterns. After being tagged on either the 2nd or 3rd of Nov all three turtles spent approximately three months at Raine Island, departing in late Jan or early Feb (Figs 32, 33, & 34).

Mertle - I759 (a combination of 'Mer' and 'turtle', was named by three students from Mer)

- Was first tagged at Raine Island in Dec 1992, returned to Raine Island five years later in 1997 and again in 2002. She was fitted with a satellite tracker on 2nd Nov 2016, 14 years after she was last sighted at Raine Island.
- She attempted to nest at both Raine Island and Moulter Cay, and used an inter-nesting habitat across four widely separated reefs—Moulter Reef, Saunders Reef, Raine Island Reef and Great Detached Reef.
- Left Raine Island on 28th of Jan, and migrated south down to Jubilee Reef, swimming 200km in six days.

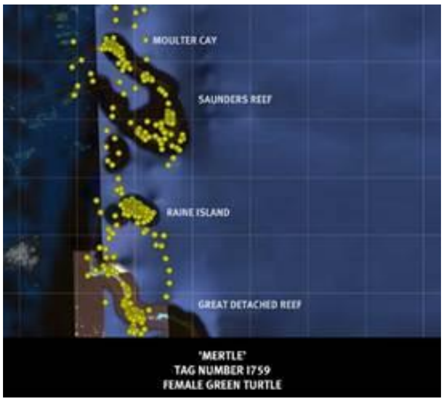

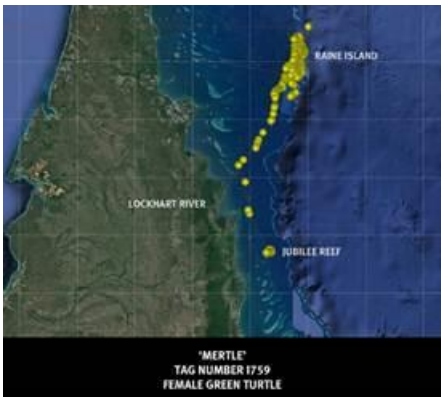

Figures 32 a & b. *Data records for GPS locations of 'Mertle' in 2016-17*

Tokolou - K74859 (named by three students from Lockhart River State School as it means turtle)

- Was first tagged in July 2006 in the foraging grounds of Combe Reef and nested at Raine Island in Dec 2006.
- Mainly used a restricted area of inter-nesting habitat on the western end of Raine Island reef, coming ashore only on the western and southern sides of the Raine Island. She was sighted by the team at Raine Island on the morning of the 1st of Feb.
- Left Raine Island on the 7th of Feb, migrating south back to Combe Reef, swimming 420km in 11 days

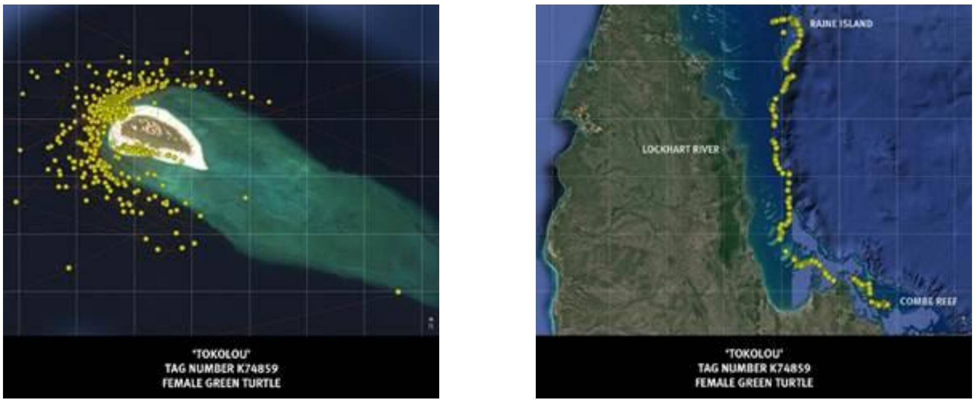

Figures 33 a & b. *Data records for GPS locations of 'Tokolou' in 2016-17*

Turturi - T7159 (a shortening of the Latin word 'turturibus', chosen by BHP Billiton)

- Was first tagged in Nov 1984 and seen again in Dec 1984, was next seen at Raine Island 15 years later in Dec 1999.
- Mostly used a restricted area of inter-nesting habitat on the north and eastern end of Raine Island reef and mainly came ashore at the western end of Raine Island.
- Left Raine Island on the 29th of Jan, and migrated south to Corbett Reef, swimming 282km in 10 days.

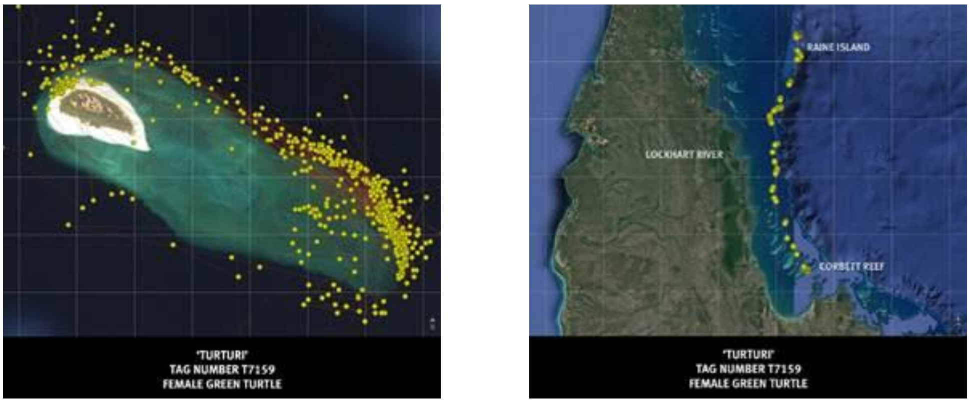

Figures 34 a & b. *Data records for GPS locations of 'Turturi' in 2016-17*

Nesting success and clutches laid:

Using the data from these three satellite tags and the two tags applied the season prior, the number of clutches laid and nesting success rate of each turtle tagged was analysed and number of clutches laid and nesting success was measured for the entire nesting season at Raine Island. The three turtles from this season, laid either seven or eight clutches over the three month period however an equal or more nesting attempts for made that were unsuccessful nesting- nesting success ranged between 26%-50%.(Table 20)

Table 20. *Successful and unsuccessful nesting attempts of satellite tagged turtles nesting at Raine Island in 2015-16 and 2016-17:*

Turtle Tag	Capture date	Successful nesting	Unsuccessful nesting	Total nesting emergences	Nesting success rate
I759	02/11/2016	7	20	27	0.26
K74859	03/11/2016	8	13	21	0.38
T7159	03/11/2016	8	8	16	0.50
T90143	01/12/2015	6	4	10	0.60
I22154	01/12/2015	2	6	8	0.25

8. REMOTE SENSING NETWORK

Methods

The remote sensing network consists of:

- Base station above the cliff at the NW end of the island with
- 2kW solar panels producing 12V and 240V power with battery bank storage
- Vaisala weather station recording wind speed and direction, rainfall, humidity, barometric pressure every 10 minutes.
- WiFi receiver station with NAS storage for remote cameras
- 2 x Satellite dishes and modem for data and image transmission and 1 x WiFI modem for local connection for IPad data collection device link to Cloud update and storage of data.
- Three remote WiFi linked monitoring stations with 0.5kW solar 12V and 240V power, 1 x HD PTZ video camera (day images) and 3 x Canon D6 still camera (night images every 15 minutes)

A team consisting of Andy Dunstan (QPWS), Rob Galessio (Activ8Me), Scott Bainbridge (AIMS) and QPWS IT section are working on:

- Upgrading the camera systems,
- on-site data and image storage,
- WiFI communications on-site,
- External data storage and sharing with research collaborators
- External links to data and images for access by departmental staff
- Links to mooring observations via PTZ video camera including SMS alerts of vessel arrivals to QPWS/GBRMPA compliance team, and
- Limited web-based access of data and images for general public and education programs

It is anticipated that this system upgrade will be in place by November, 2017.

Results

NBN satellite installation

During December 2016 a new satellite connection system was installed at Raine Island by Activ8Me in conjunction with NBN to link through the SkyMuster satellite. This dual installation provides redundancy and the ability to use either system to ensure confidence of transmission from the harsh Raine Island conditions. Modems and WiFi units are durable to suit the conditions and provide WiFi access from the vessel at the mooring and also around the island up to 300m distant. This provides valuable WiFi connectivity for upload of all field research data collected electronically on IPads or remote sensing devices immediately to the cloud for safe storage.

The systems have been running continuously and reliably even during the most challenging conditions of torrential rain and extreme cloud cover since installation.

The capabilities of each of the two systems are

- 150GB Anytime data
- Speed Mbps 25 download /5 upload
- Monthly Plan Charge $296.99ex

Weather station data

The weather station data has been downloaded and is included within data analyses and relevant presentations within this report. The most relevant data collected is for rainfall, barometric pressure, wind speed and direction and air temperature. Wind data has been limited due to sensor damage by roosting seabirds but new protective measures are about to be installed to hopefully rectify this.

dGPS base station

A permanent dGPS base station is in place, powered through the base station and linked through the satellite system for control, adjustments and checking off-site. This greatly improves the ease and efficiency of the surveying required for various elements of the RIRP research and monitoring program.

Remote still cameras

Images from the re-profiled sector camera have been analysed for total turtle counts and nesting turtle distribution within a standard area of the image frame.

Remote PTZ video cameras

Are not active now but are planned to be upgraded and re-installed in August 2017.

9. SEABIRD MONITORING

8a. Red-tailed tropicbird monitoring

Methods

A separate survey is conducted by a team of two or three persons for the red-tailed tropicbirds as their nests are located in the cliff perimeter and associated outlying rocks of the Island. Full red-tailed tropicbirds surveys were conducted during July 2016, Nov 2016, Dec 2016, and April 2017. All potential nesting and roosting sites in the escarpment and associated scree were searched and new sites locations recorded by differential GPS. All occupied sites by adult birds were then inspected to ascertain whether the birds was on a nest.

Results

A total of 208 adult tropicbirds were found and 261 active nests (egg, chick, young or adolescent) were identified across the four surveys in 2016-17 (Figs. 35 & 36). Note active nests is the cumulative total of all active nests found in the four surveys, it does not take into account reproductive success of individual nests. Some nests are present in multiple surveys as previously found nests from earlier surveys were re-visited and the current status of breeding effort recorded.

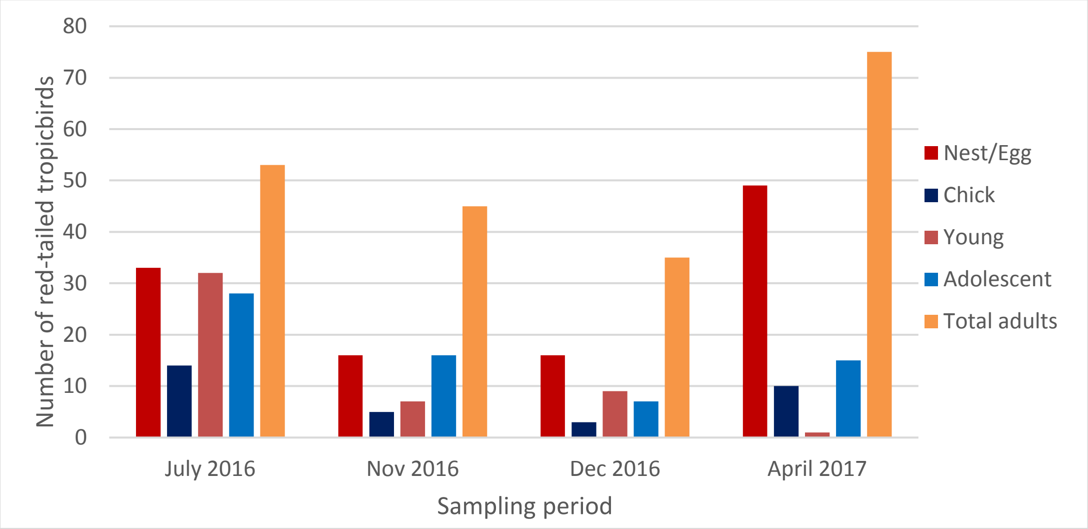

Figure 35: *The number of red-tailed tropicbirds by status of breeding effort and number of adults found across the four surveys*

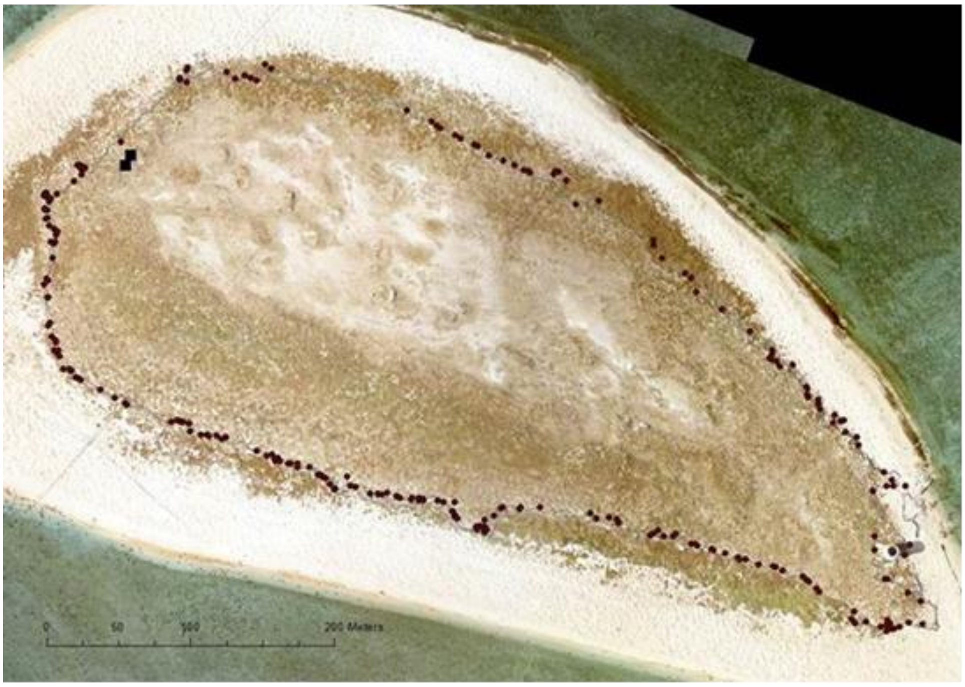

Figure 36: *Location of red-tailed tropicbird nests for 2016-17 surveys*

8b. Standard seasonal seabird monitoring

Methods

Seabird surveys, as per the Raine Island Standard Operating Procedure, were conducted as soon as possible upon arrival. These surveys require a team of two or three persons, with at least one member having completed the QPWS Coastal Bird Monitoring Training Program. The team moves around the Island using vantage points (i.e. any area where a large area of the Island is visible and results in minimal disturbance to birds) to accurately count birds and determine breeding effort. All adults regardless of nesting or loafing are counted and breeding effort is determined by number of nests, chicks and young counted. Surveys were conducted at Raine Island in July 2016, Dec 2016, and Jan 2017, and at Moulter Cay in Dec 2016 and Feb 2017. Separate surveys were undertaken to monitor red-tailed tropicbirds as reported in a previous section.

During winter months, vegetation is present on the swale requiring a modification to the seabird survey to count common noddies. A transect method was used during the July 2016 survey to estimate the density of the common noddies breeding at Raine Island. A zig-zag transect between the cliff and outside of the vegetation was walked for the entire island, followed by four 100m straight line transects above the cliffs. An estimate of the total number of common noddies breeding on the island is calculated from these counts based on the area of swale vegetation and area above the cliff.

Results

Raine Island supports at least 20% of the proportion of breeding populations occurring along Queensland's east coast for eight species of seabirds that nest there. The breeding effort determined from the three Raine Island surveys for these eight species are presented in Figure 37. Note that breeding effort does not include the number of adults present during the surveys although adults may have been counted during the survey they are only included if they were nesting. As such, in the July 2016 survey 6 herald petrels were recorded however there were no signs of nesting and therefore are not included in Figure 37.

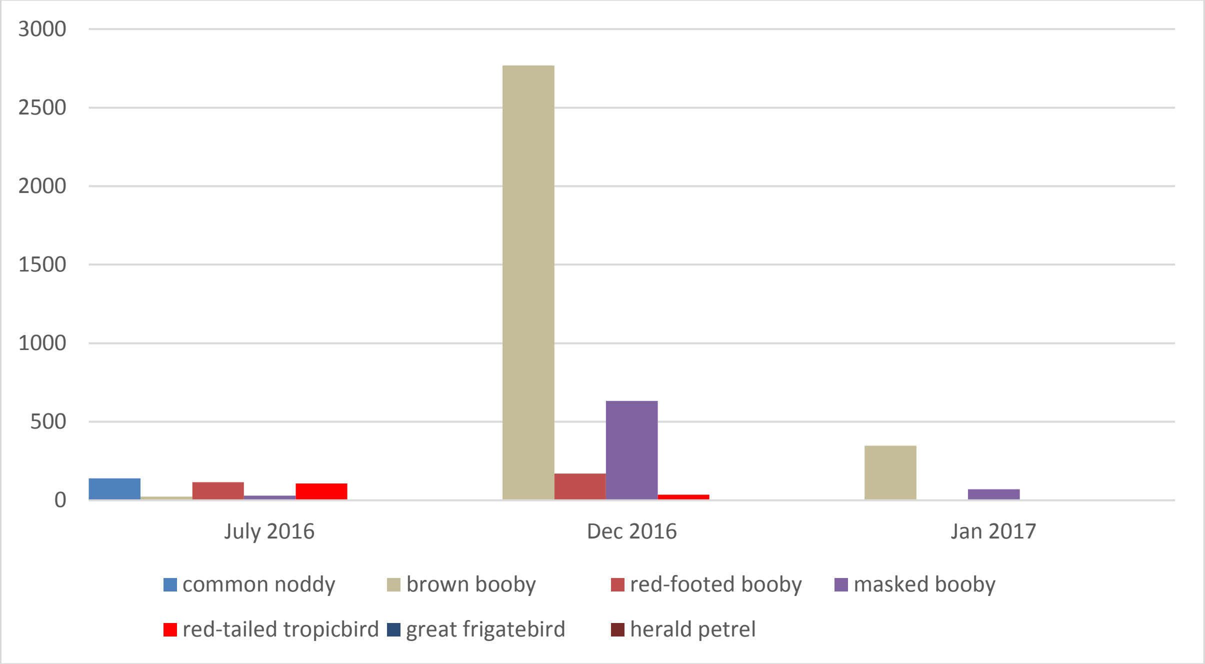

Figure 37. *The total number of breeding pairs for eight seabird species counted at Raine Island across the three surveys. Note breeding effort does not include number of adults.*

8c. Red-tailed tropicbird artificial nest boxes

Red-tailed tropicbirds nest in the cracks and crevices at the bottom of the cliffs and to mitigate possible disturbance from management actions such as sand re-profiling, artificial nest boxes have been installed above the cliff. Ten nest boxes were installed in July 2016. Data-loggers (temperature and humidity) were placed in the natural nests and the artificial nest boxes to monitor how closely the boxes match natural conditions on the island as climate within the boxes was identified as likely major influencing factor for their use. Natural nests were selected as they had previously been used as nest site but was not being used as a nest site at the time of selection and were as close to the artificial nest box location as possible. The uptake of the artificial nest boxes are monitored as part of the red-tailed tropicbird survey.

Results:

No red-tailed tropicbirds have been observed utilising the nest box. One adult brown booby with chick were recorded utilising a box however it is unknown whether this was an opportunistic use during the heat of the day or whether the bird nested in the box.

Initial results from the data from the data-loggers show that the nest boxes mirror the climatic conditions in natural nests (Fig. 38).

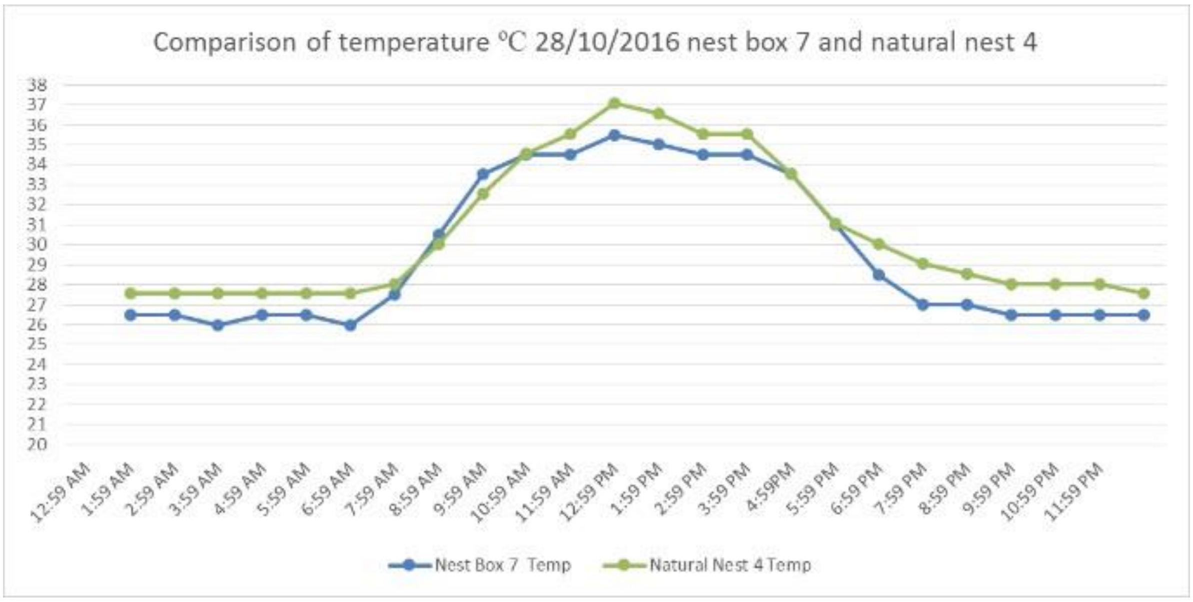

Figure 38. *Comparison of temperature recorded over a 24 hour period in nest box number 7 and a natural nest nearby.*

8d. Acoustic monitoring of nesting seabirds

The acoustics offer an opportunity to generate reliable bias free estimates of nesting birds species, particularly those that breed in large homogenous aggregations such as common noddies and boobies. They are probably less useful for birds in discrete colonies as the proximity to microphones may be a significant issue, however, the most important colonial nesters (lesser frigates) do nest in the same three or four locations each year so it may be possible to place microphones in each of these sites.

Complex learning algorithms covert raw noise to a calls per minute for each species. Ground/drone counts will be continued around each site to validate these correlations to generate reliable scaling methods to convert acoustic data to nesting density data. The overall aim is to provide year-round monitoring of as many species as possible without observer bias.

Methods

25 acoustic logger units were installed in April 2017 with sandbags at edges of four quadrants 10m from the logger (Figs. 39 & 40). Ground observers and drones were used to obtain counts of seabirds within this 10m radius and these counts used to validate signals. A contract is in place with Conservation Metrics to analyse data using a Deep Neural Network.

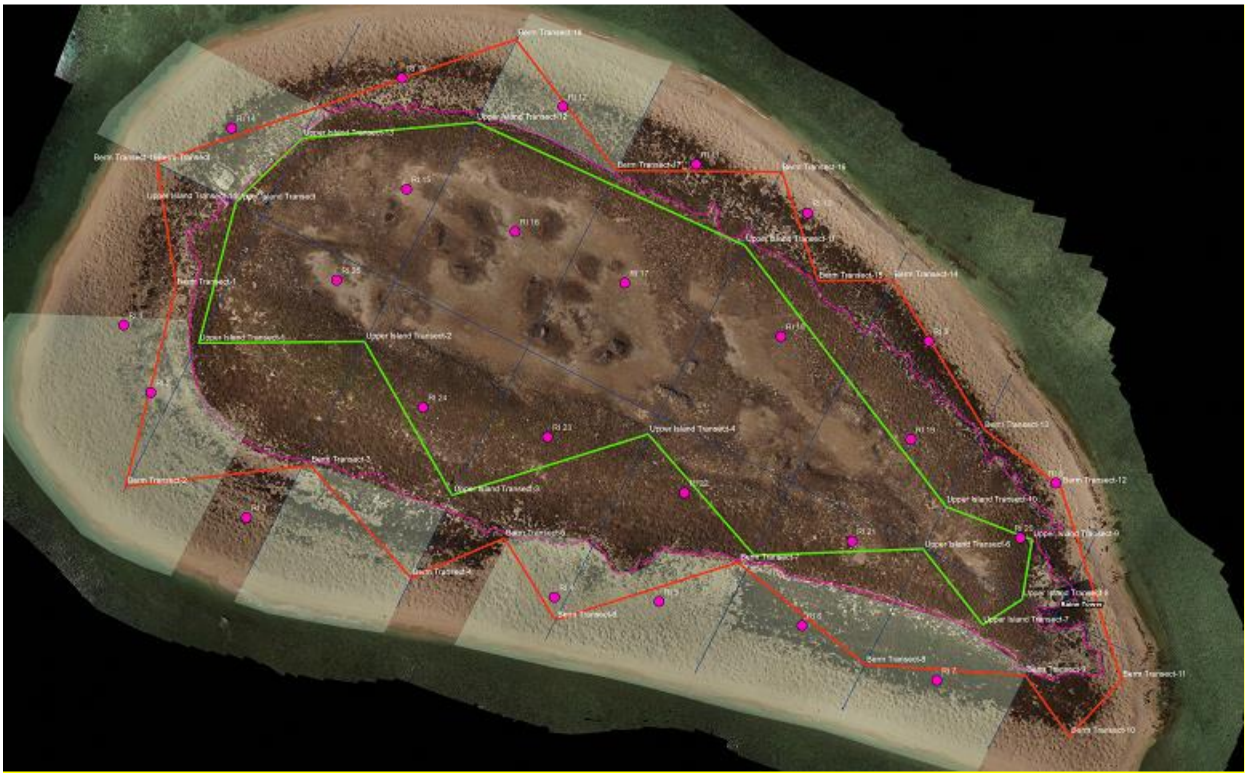

Figure 39. *Map of acoustic logger locations and seabird survey transects at Raine Island.*

Red lines are current island top transects, yellow are beach transects, red dots are acoustic sensors, pink grid is original monitoring grid and green lines are extents of vegetation as measured on field trips. Note we assume we can count all birds in open areas (white shapes in centre of island) with 100% accuracy hence no transect therein.

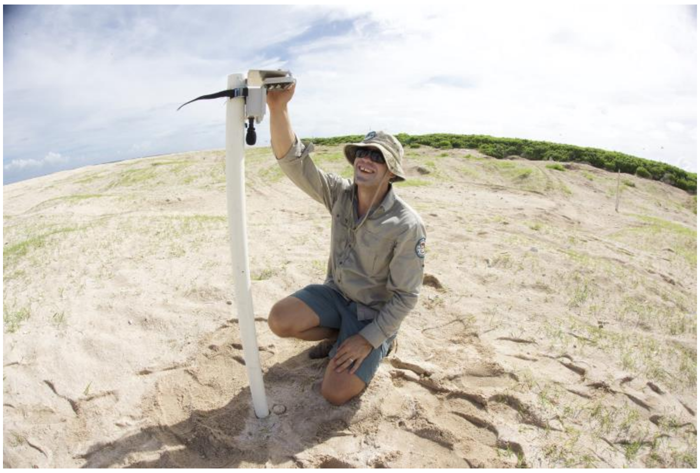

Figure 40. *Installation and initialisation of an acoustic monitoring station on Raine Island beach*

9. ELECTRONIC DATA ENTRY AND MANAGEMENT

- Five Ipads were programmed for data entry for 2016-17 season
- Re-profiled successfully with supportive feedback from staff – easier, more efficient and with increased capacity for new data inclusion. E.g GPS locations for dead turtles
- Efficient upload via WiFi / Satellite to cloud data storage immediately on-site
- Standard electronic fields and forms replicate original paper data sheet forms for turtles, seabirds, weather, trip details, environmental sensors and standard island images
- 2016-17 season data was collected both on paper and electronic data sheets
- Database development is underway for management, analysis and reporting of the data.

10. TIGER SHARK RESEARCH

No tiger shark research was conducted during the 2016-17 season

11. BIOSECURITY

All Raine Island trips now more formally address biosecurity issues and the potential introduction of pest flora and fauna species to Raine Island. This includes:

- briefing documents, permits and presentations
- quarantine level checks and cleaning of anything to be brought onto the island
- environmental protocols (such as seabird nesting area access restrictions)
- cultural protocols
- tower access rules
- mooring restrictions
- vessel crew island access restrictions

12. EQUIPMENT AND INFRASTRUCTURE

FILMING

Biopixel filming

An MOU agreement is being formalised between Biopixel and EHP media department to have Richard Fitzpatrick join Raine Island expeditions to film in highest quality 4K digital vision. He will be directed to film everything possible relevant to management and creation of vision to document the Raine project. This vision will be owned jointly by EHP and Biopixel. Natural history vision will also be recorded and be owned by Biopixel but raw footage will be supplied to EHP for non-profit use. 20% of profits from sale of all footage will go to the Raine project.

Richard was present on most of the Raine trips this season and excellent footage is now stored at EHP/QPWS media unit. A Digital Asset Management system is currently being implemented to catalogue imagery for safe storage and ready access.

RAINE ISLAND RECOVERY PROJECT CORPORATE SPONSORSHIP

A contract is now in place between The Great Barrier Reef Foundation and the Queensland Government for sponsorship of $7.95M over 5 years for the Raine Island Recovery Project. The main project sponsor is BHP.

13. INDIGENOUS TRAINING AND RELATIONSHIPS

Strong relationships within the Raine Island Project now exist between EHP, QPWS, GBRMPA, Balkanu and TSRA. Involvement of Indigenous rangers and the Raine Island Traditional Owners from the Kemer Kemer Meriam Nation (Ugar, Mer, Erub) and Wuthathi Nation occurred in all trips this season, with 140 paid days of employment.

14. MOULTER CAY MONITORING AND COMPARISON TO RAINE ISLAND

Nesting success

Nesting success surveys have been conducted during December at both MoulterCay and Raine Island five times. At no time were these surveys conducted on the same nights for exact comparison. In general Moulter Cay has demonstrated higher nesting success levels than Raine Island, however the overall outcome has still been that of low nesting success levels at Moulter Cay in early to mid-December (Table 21 & Fig.41).

Table 21. *Nesting success data from all seasons where data was collected*

	Nesting success	
**SEASON**	**Moulter**	**Raine**
2006	0.13	0.04
2007	0.74	0.42
2008	0.06	0.10
2015	0.48	0.28
2016	0.14	0.12

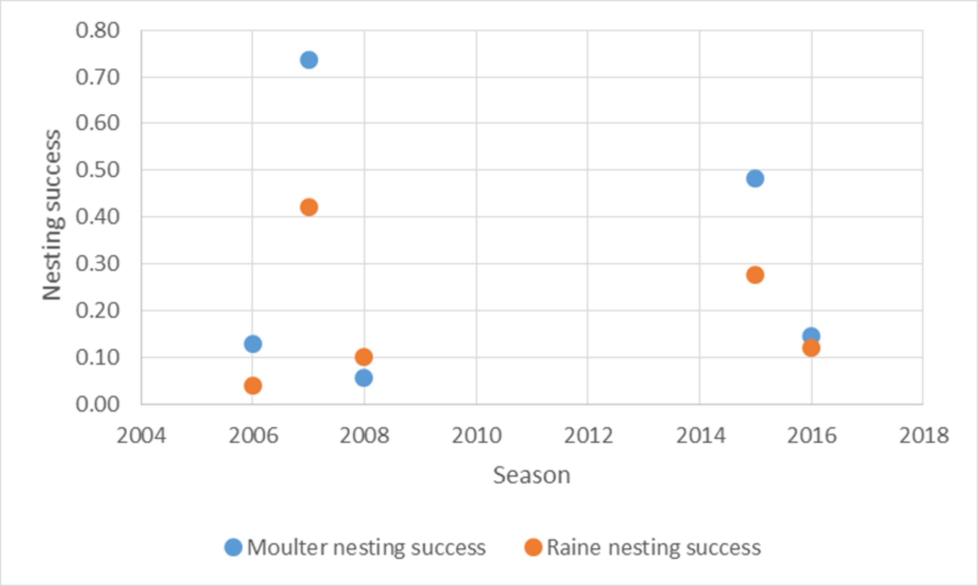

Figure 41. *Comparison of Raine Island and Moulter Cay nesting success data*

Tally count

Seasons where tally counts were conducted at around the same time in early December at both Raine and Moulter are compared.

Moulter Cay has around 50% of the tally count numbers of nesting turtles compared to Raine Island during December (Table 22).

Table 22. *Comparison of tally count data from Raine Island and Moulter Cay*

SEASON	Moulter	Raine	Ratio
1976	430	872	0.49
1977	13	32	0.41
1982	402	925	0.43
1985	157	151	1.04
1986	1807	3957	0.46
1988	627	731	0.86
1992	180	1323	0.14
1993	5057	8593	0.59
1995	2495	4648	0.54
1996	4043	12100	0.33
1997	1624	4065	0.40
1999	3665	7292	0.50
2000	67	131	0.51
2001	2022	3601	0.56
2002	474	1813	0.26
2004	3824	6265	0.61
2006	6099	17381	0.35
2007	90	119	0.76
2008	1562	10836	0.14
2010	4130	6948	0.59
2011	3313	6144	0.54
2013	6738	10584	0.64
2016	1384	5496	0.25
		**Mean**	**0.50**
		**S.E**	**± 0.04**

Turtle mortality

Mortality recorded at Moulter Cay was less than that recorded at Raine Island to a ratio similar to that of nesting turtle (tally count) ratios recorded.

A large percentage of the mortality at Moulter Cay is due to beachrock entrapment on the northern side of the island. This is underestimated as dead turtles are washed away and/or predated on the spring tides and not recorded (Table 23).

Table 23. *Comparison of Raine Island and Moulter Cay turtle mortality numbers and causes of death for 2016-17*

Date	Heat	Cliff falls	Cliff entrapment	Beachrock	**TOTAL**
RAINE	103	51	13	2	**169**
MOULTER	35	6	1	23	**65**

Hatchling production

The 2016 season comparison of hatchling production at Moulter Cay was hampered by adverse weather. A single night of counts was conducted from 1800 – 2100 hours.

Moulter Cay recorded a higher hatchling production than on any of the three sampling nights at Raine Island however these were conducted more than a week earlier. The April Raine Island data is presented for comparison below (Table 24). Timing of rainfall to induce hatchling emergence may be an influencing factor.

Table 24. *Hatchlings counts from Raine Island and Moulter Cay in February 2017*

Time	Raine Island							Moulter Cay		
	Sector A2			Sector B				Sector A	Sector B	Sector C
	2/02/2017	3/02/2017	4/02/2017	2/02/2017	3/02/2017	4/02/2017		13/02/2017		
pre-1800	19	0	0	0	0	0		0	0	0
1900	7	0	0	0	18	2		73	49	12
2000	100	99	45	27	71	83		314	203	67
2100	73	65	183	185	212	306		157	647	400
**TOTAL**	**199**	**164**	**228**	**212**	**301**	**391**		**544**	**899**	**479**

Hatching success – Clutches laid early Dec and excavated early Feb

14 nests dug successfully (12 berm, 1 back berm and 1 swale) – 72.3 ± 7.8% emergence success compared to Raine Island: 60.6 ± 3.4%

Swale nest inundated with total emergence failure at stage 1.

25 nests not found – 11 gone, two washed away on front of berm and 14 with newer nests in same location.

Clutch destruction – 59.9% compared to Raine Island – 59.1%

Nesting beach inundation and sub-surface rock layer

Inundation is of a similar height as that seen at Raine Island and the Moulter Cay nesting beach is of a similar topographic profile. Most of the swale is inundated at nest level during spring tides.

Holes were augered in representative locations of the berm, back berm and swale throughout the nesting beach to sub-surface rock layer level and mapped with dGPS. Most of the swale and parts of the back berm had a rock layer much shallower than the 50-80cm required for successful green turtle nesting.

Data is currently being analysed.

Discussion

The 2016-17 fieldwork season at Raine Island has produced valuable outcomes for directing future management actions, evaluation of adaptive management work and the continuation of existing research and formulation of future research needs. It has resulted in much better understanding of the factors influencing nesting and hatching failure of turtles on Raine Island. It has also trialled and implemented new research methods, including use of innovative new technology.

Results of adaptive management actions already undertaken demonstrate their effectiveness.

Reducing nesting female turtle mortality:

- Cliff-top fencing installation has reduced overturned turtle mortality by more than 70%, saving over 400 turtles.
- Machinery assisted turtle rescues now enable staff to rescue all turtles during their presence at Raine Island equating to hundreds of mature female turtles during peak seasons.

Increasing reproductive output. Nesting beach re-profiling has:

- Created four times more viable nesting area in the re-profiled sector than previously
- Increased hatching success
- Increased nesting efficiency
- Decreased nesting density (turtles spread out to nest throughout the entire area), resulting in reduced clutch destruction and nesting turtle disturbance

- Increased hatchling production

New experimental methods are answering or coming closer to answering some of the major questions. The key questions remain focused on quantifying nesting and hatching failure and identifying the cause(s) of this.

More robust techniques introduced in 2013-14 have been continued and/or refined and include:

- quantifying nesting success, numbers of eggs actually laid and distribution of nests and nesting effort within the nesting area
- estimating total numbers of breeding female turtles aggregating to nest at Raine Island
- estimating hatchling production
- quantifying clutch success and developmental stages where egg mortality occurs and
- quantifying clutch destruction
- measuring water table levels and 3D mapping nests and inundation impacts

Results of nesting behaviour studies clearly show more evenly distributed nesting throughout the raised re-profiled sector when compared with controls. Reduced clutch destruction is very likely due to the more evenly and therefore sparsely distributed nesting turtles. The minimal slope from berm to cliff in the re-profiled sector is probably the reason for the even nesting distribution as turtles in other sectors concentrated their nesting efforts on the berm, but the absence of vegetation in the re-profiled sector may also be a factor.

In comparison to the catastrophic failure in reproduction in 2013-14 (<1% hatchling output), the 2014-15 season showed much higher success and the 2016-17 season even higher success. Analysis of the data from the previous five seasons where comparative nesting and hatching data was recorded has produced some valuable outcomes.

The analyses show

- nesting success improvement with moisture content of sand
- hatching success has a negative correlation with nesting population size
- clutch destruction is directly proportional to nesting population size
- hatchling production per clutch is negatively correlated with nesting population size
- very early phase death of embryos is the major timing for hatching failure
- hatching failure is higher in the swale and back swale and also in the north-west section of the nesting beach where nest-level inundation is more prevalent

Egg death outside inundation zones points to other major additional causes of hatching failure. Microbial load and subsequent O2 reduction and temperature increase impacts on the nest environment as well as potential toxin effects require investigation. Reduced viability of eggs retained in oviducts during repeated nesting failure prior to laying may also be a key factor. The impact of grain size and moisture conduction above the water table also needs examination.

Adult nesting turtle mortality was low, correlating with a very low density-nesting season. Cliff fall deaths were low especially in areas where fencing is present, continuing to demonstrate its value in mortality reduction. More fencing installation is required including the possible need for adaptation of fencing methods to counter rock falls as nesting turtles undermine the cliffs. Large rocks at the tower south-eastern end of the nesting beach are a major cause of overturning mortality and reshaping of these rocks may be greatly reduce mortality.

The remote sensing network is working well and delivering valuable data. Weather, inundation level and sand temperature data is presented in this report. Night images of the turtle nesting beach in four quadrants of the island will be analysed to provide full season information on turtle nesting numbers and distribution and changes to the nesting beach vegetation. This method and all new methods implemented will be calibrated against existing methods until consistent and statistically valid comparisons of data can be made. Time-lapse imagery will be an invaluable presentation tool. A website is currently being created to support live streaming of the remote sensing data and imagery, initially for in-house purposes and later for public education and awareness.

The use of drones for topographic mapping, seabird census and turtle counts was highly successful and offers new opportunities for research and increased efficiency for many areas of current research. The research to test the impact of drones on nesting seabirds was successful in developing minimal impact procedures for use of drones at Raine Island.

Acknowledgements

The Raine Island Recovery Project is a five-year $7.95 m collaboration between BHP, the Queensland Government (Queensland Parks and Wildlife Service and Department of Environment and Heritage Protection), the Great Barrier Reef Marine Park Authority, the Wuthathi and Kemer Kemer Merian Nation (Ugar, Mer, Erub)

Traditional Owners and the Great Barrier Reef Foundation to protect and restore the island's critical habitat to ensure the future of many marine species, especially marine turtles and sea birds.

Collaboration with experts from James Cook University, Australian Institute of Marine Science, University of Queensland, Auckland University of Technology and Biopixel has also been an integral part of the project. Volunteers have been invaluable to the fieldwork effort and are thanked for their enthusiasm and hard work.

References

Dunstan, A.J. (2016). Raine Island Recovery Project: 2015-16 Season technical report to the Raine Island Scientific Advisory Committee and Raine Island Reference Group. Brisbane: Department of National Parks, Sport and Racing, Queensland Government.

Limpus, C. J., Carter, D. and Hamann, M. (2001). The green turtle, Chelonia mydas, in Queensland: the Bramble Cay rookery in the 1979-1980 breeding season. Chelonian Conservation and Biology 4(1), 34-46.

Limpus, C. J., Miller, J. D., Parmenter, C. J. and Limpus, D. (2003). The green turtle, Chelonia mydas, population of Raine Island and the northern Great Barrier Reef: 1843-2001. Memoirs of the Queensland Museum 49(1), 349-440.

Miller, J. D. (1985). Embryology of Marine Turtles. In Biology of the Reptilia. Vol 14. Development. A. (Eds C. Gans, F. Billett and P. Maderson.) pp. 270-328 (John Wiley and Sons: Sydney).

Limpus, C.J. (2008). A biological review of Australian marine turtle species. 2. Green Turtle, Chelonia mydas (Linnaeus). The State of Queensland, Environmental Protection Agency.

APPENDIX

Appendix 1

Preliminary Scoping of Potential Sand Movements on Raine Island during 2017.

Scott Smithers

John Dawson

Environmental Management Group, College of Science and Engineering, James Cook University, Townsville.

INTRODUCTION

Sand movements to improve turtle nesting and hatching success are scheduled for the second half of 2017. To plan for these movements a meeting occurred in Townsville in early March to outline and scope potential options.

An outcome of this meeting was that we would do some preliminary calculations on sand volumes and offer some preliminary thoughts on potential issues or concerns associated with potential sand movement at three possible sites (Figure 1).

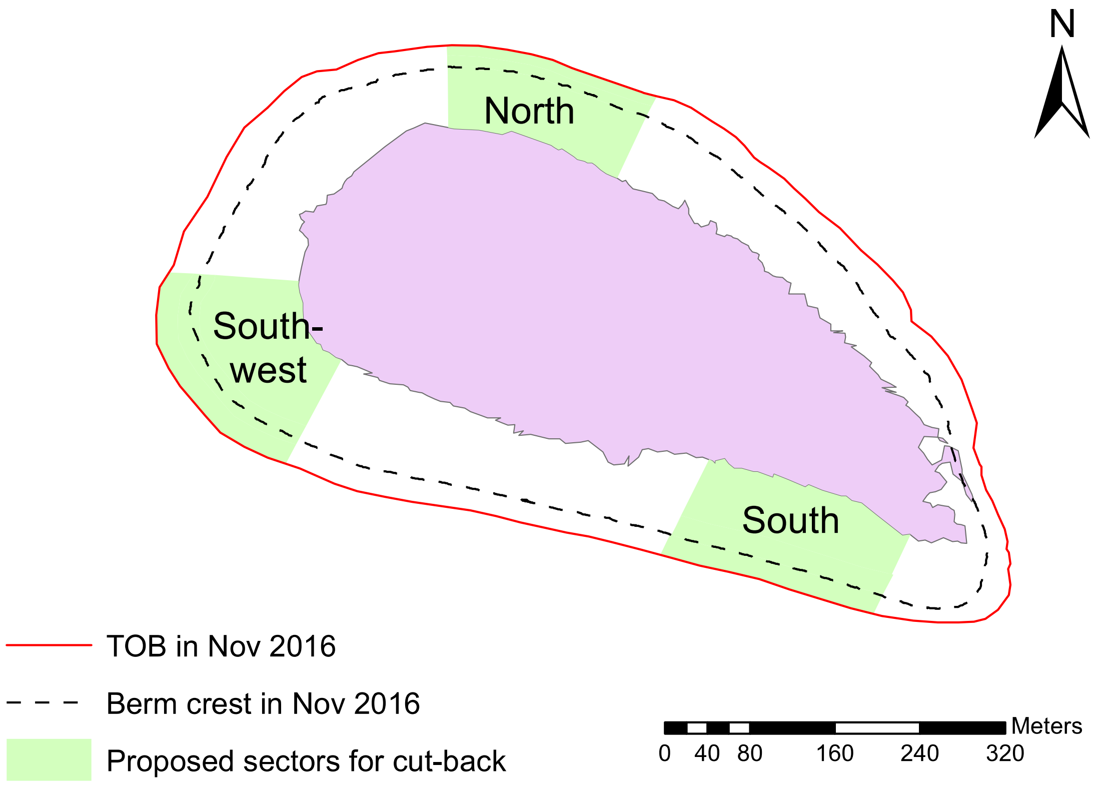

Fig. 1. Location of potential sectors for sand movements. The location of the toe of the beach (TOB) and the berm crest surveyed in November 2016 are shown and used in the calculations that follow. The reasons and limitations of using this shoreline position are discussed further below.

The outcomes of our preliminary calculations are reported below.

VOLUME CALCULATIONS

Sand volumes were calculated for three different shoreline cut back amounts at each site, where the cutback amount is the distance the berm crest is translated shoreward. For each cutback scenario the volume of sand available for movement is that beneath the area defined by the length of the sector and the distance the berm crest was translated. The beachface volume is not included in the available harvest volume as a beach is clearly needed on the remodelled shoreline and we are assuming that it would require a similar volume of sand as the existing beachface.

An important caveat on the following report is that it is predicated on the assumption that there is no beachrock under the berms to be harvested for sand to be rolled back to raise the elevation of the lower swale areas. This assumption may not be true, and thus the volume of available sand may be overestimated. Accordingly, a systematic and detailed investigation of beachrock outcrops and available sand depths/volumes should be a priority.

VOLUME CALCULATION RESULTS

Table 1 summarises the preliminary sand volume calculations calculated for each of the three potential treatment sectors with three potential cutback amounts and two different final surface levels (4 m LAT and 3.7 m LAT) investigated. The calculations were undertaken using the November 2016 geomorphological model of the island as the baseline (Figures 2 and 3).

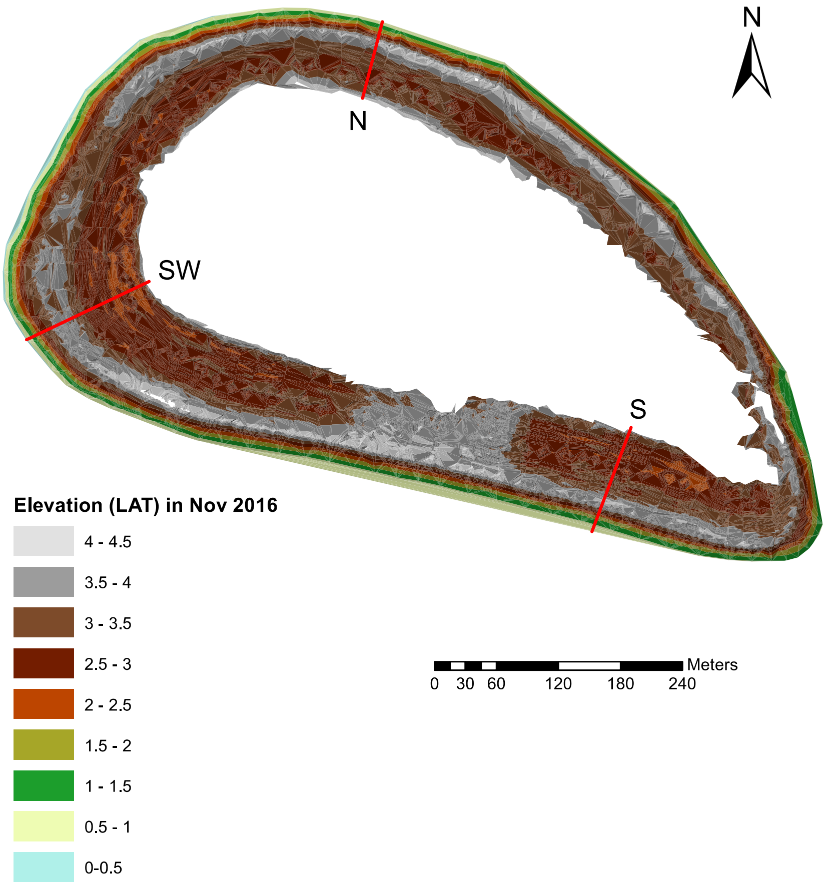

Fig. 2. November 2016 morphological model of berm areas, showing elevations and locations of cross-sections used to estimate infill volumes (see Fig.3).

We chose this model as it is likely to be most like the island would be in late September / early October when we believe the proposed sand movements will take place. We emphasise, however, that the island may not be the same at that time, and thus the volumes here may not be available if there is a significant difference in Raine Island's shoreline configuration and morphology at the time of sand movement compared to the November 2016 state. An accurate survey prior to undertaking the works would be necessary to assess the compatibility of the island's morphology and thus the utility of the numbers below for guiding volumes for harvest.

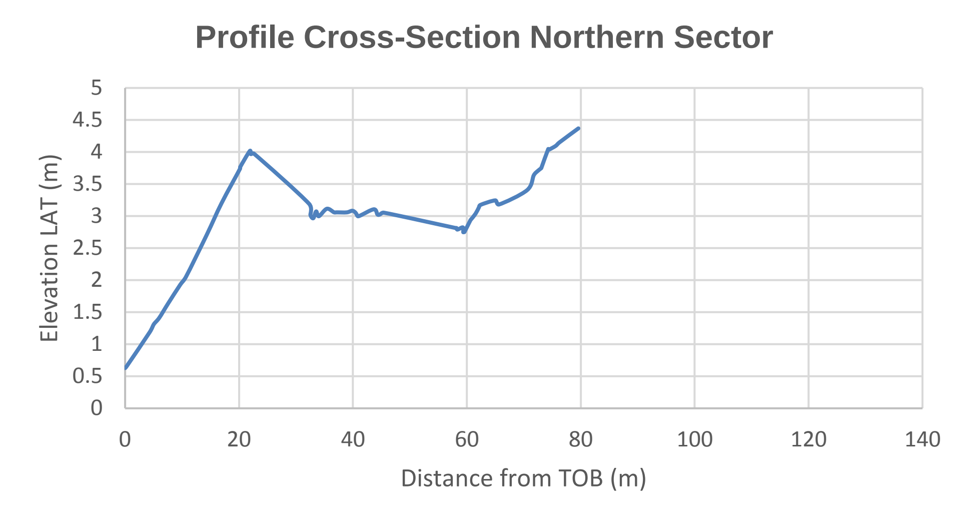

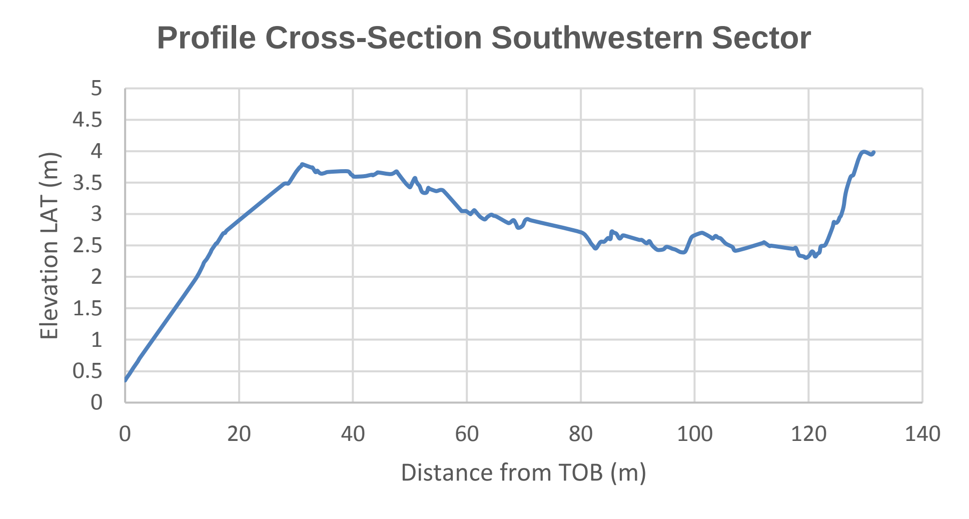

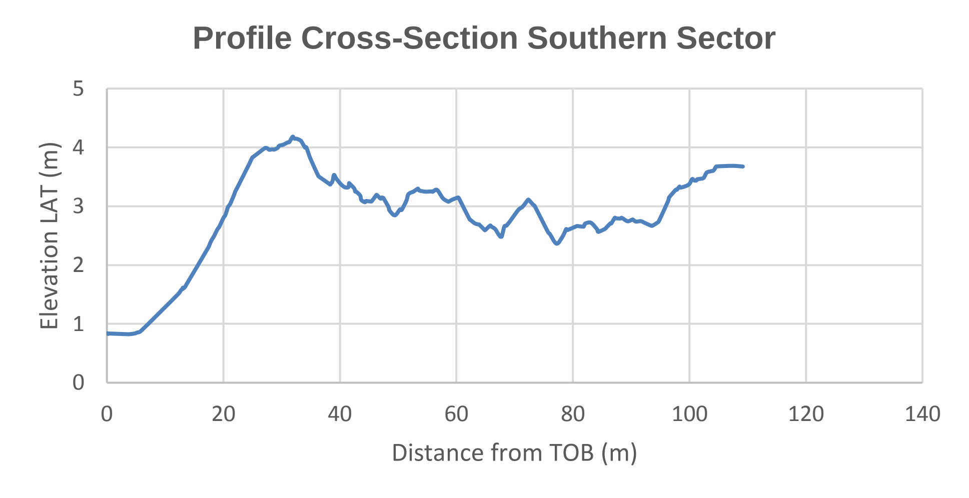

Fig. 3. November 2016 representative cross-sections showing elevations across berm and swale areas used to estimate infill volumes (see Table 1).

Table 1: Calculated sediment volumes available for three cutback scenarios at each potential sand movement site and the calculated volumes required to raise the surface of swales to 4 m and 3.7 m LAT. Green cells indicate the cutback required to find adequate volume to fill swale to 4 m ***assuming no beachrock.***

**North**	**Volume available from berm**	**Volume required to fill to 4m LAT**	**Volume required to fill to 3.7m LAT**

10m cut-back	4924	6574	4064
15m cut-back	9032	6160	3945
20m cut-back	10985	4852	3160
**Southwest**			
15m cut-back	8430	13174	9245
20m cut-back	12299	12490	9000
25m cut-back	15890	11658	8698
**South**			
10m cut-back	4841	13876	9729
15m cut-back	9590	13639	9734
20m cut-back	12340	12352	9059

DISCUSSION

The volume calculations above indicate that the minimum cutback amounts required to yield sufficient sand volume to fill the swales to a level of 4 m LAT are ~15 m for the northern sector, 20 m for the southwestern sector, and 20 m for the Southern sector. Critically, these volumes / cutback distances assume that the entire sand volume liberated by the cutback is available for redistribution, and that no beachrock is encountered beneath the translated section of beach.

An examination of our existing survey data as well as digitized shoreline (toe of beach) positions from available aerial photography and other imagery with adequate resolution for us to confidently map this feature allows us to determine whether the shoreline has over the period for which this data is available ever migrated shoreward past the proposed cutback distance, and if so whether any beachrock was or was not exposed at that time. A summary of this analysis is presented in Figure 4 below. In Fig. 4 the extent of a 20 m cutback is indicated in the tan colour for each sector, and the most shoreward position of the shoreline documented in our records is shown by the red or blue lines. For both the northern and southern sector the maximum shoreline retreat position we have a record or occurred in November 2012, and for the southwestern sector it occurred in Feb/Dec 2015. Our interpretations of these analyses for each of the proposed sectors are summarized below.

a) Northern Sector.

A smaller cutback distance (~15 m) is required at the northern sector to yield the necessary sand volume in this sector than at the other two, however, beachrock is already exposed on the beachface along this section of the shoreline, and it is clear that the contemporary shoreline is very close to its maximum point of retreat for which we have evidence. It is very likely therefore that the volumes of sand required will not be locally available in this sector. The northern shoreline has been relatively stable over the past few decades, probably in part reflecting the existence of the beachrock along this shoreline. It is our view that Interference with this beachrock would be potentially risky, as it may destabilize the shoreline. If this sector is to be targeted, more detailed investigation of how such destabilization may progress would be advised.

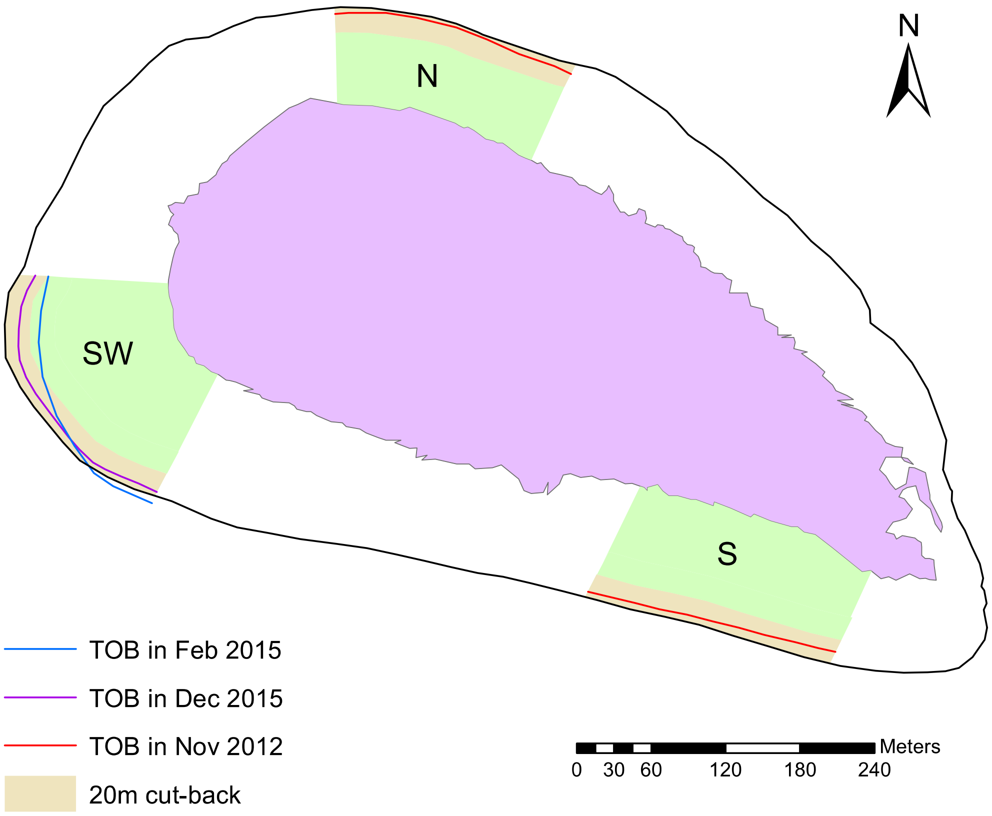

Fig. 4. Figure showing sectors and positions of maximum documented shoreline retreat in each available from our survey and digitized imagery records.

b) Southwestern Sector.

A 20 m cutback distance is required to yield the necessary sand volume in this sector. How this amount of cutback compares with the known position of maximum retreat varies through the sector. In the SE quadrant it is significantly landward of the maximum retreat position but on the western and northern section it is located seaward. The implication is that cutback at the western and northern section should be possible without encountering beachrock, but this cannot be guaranteed for the southern / eastern end of the sector. This end of the island is very dynamic and the shoreline very changeable from year to year. This is because small variations in wave climate and direction can have a large morphological effect on this curved section of coast, where the reef flat is also deeper and narrower than along other sections facing the island. As a result, during the northwesterly season sand movements along this shoreline can be very active, and the morphology and position of the shoreline can be very dynamic.

Sand movements on this section of shoreline require careful consideration as they come with significant risk. Firstly, the timing of the works is problematic as the mechanized cutback would be occurring at a time when the modified shoreline would continue to be exposed to weather from the northwest that would likely continue to ‘naturally’ erode the modified coast for several months during the NW season. This could potentially push the shoreline translation well beyond the planned 20 m cutback limit. The second risk is that removal of sand from this sector will not only affect the shoreline in this sector, but also fairly rapidly affect the shoreline in adjacent sectors. A sediment deficit (due to normally transported sediment not being available as it has been relocated onshore) at the southern section of this sector and on the sector immediately east of it would be expected to cause shoreline retreat. If this retreat is significant enough, particularly on what is now a straight section of shoreline, it is possible that a beachrock spur may be exposed. Such spurs have proven to be very problematic for nesting turtles on other islands such as Moulter Cay, and should clearly be avoided. In short, this section of shoreline is very dynamic, will be exposed to energy immediately after any proposed works, and the locus and amount of sediment movement is more difficult to predict as small variations in wave direction and magnitude have large impacts. One potential impact could be the exposure of a beachrock spur on the southern coast. Investigations to determine if such beachrock exists to be exposed by shoreline retreat should at the very least be done before this sector is preferenced.

c) Southern Sector.

A 20 m cutback distance is required to yield the necessary sand volume in this sector. This amount of cutback would translate the shoreline to a position more shoreward of its furtherest landward position we have been able to document. Encountering beachrock is thus a possibility. Harvesting sand to modify the re-profiled sector immediately to the west of this sector did not encounter significant beachrock, but the amount of cutback there was 14 m not 20 m. The shoreline on this section of the island is relatively linear, and thus shoreline behavior over this sector is less difficult to project than for curved sections of coast. Works to redistribute sand at the end of the southeasterly trade wind season would mean that it is likely that the coast here would be relatively stable through the following monsoon, and would in fact be relatively protected from the northwesterly winds and waves that prevail during that season. As such, the cutback shoreline would not be likely to continue to erode through natural processes after the mechanized shoreline movement, and may in fact infill with sediment moving alongshore from the west over this season. Sediment delivery from the reef flat would then also occur in the following SE trade season. This is a relatively low risk site.

PRELIMINARY CONCLUSIONS

The preliminary report above has quantified the potential sand volumes available and required to fill the swales to 4 m and 3.7 m above LAT. Less cutback is required to yield the required sand volume at the northern sector, but the evidence suggests that any work there will encounter beachrock which will reduce the available sand. Further, if the beachrock is disturbed other issues with shoreline stability may potentially arise. It is hard to recommend the northern sector as the priority site.

Both the SW and the S sectors would require 20 m cutback to achieve the required sand volumes. At the SW sector this would involve retreating the southern part of the sector well beyond where records depict it ever being at, and because of the timing of the works, this cutback shoreline would be exposed and vulnerable to continuing erosion and retreat through the NW monsoon season. This would potentially result in significant shoreline modification, and possible expose beachrock on the southern shoreline. This is a risk that could be further quantified with investigations of beachrock occurrence in the potentially affected area.

The southern sector is a relatively straight shoreline that will be protected for several months after any works. The shoreline is thus unlikely to erode beyond the cutback position in the months following the works, and in fact some of the cutback my infill sediments moved alongshore from the west. The straight shoreline also makes projection of shoreline behavior across the sector less complex. Previous work on the adjacent section of shoreline did not encounter beachrock with 14 m of retreat, but 20 m of retreat is required to gain the necessary volume yield. Although it is possible that beachrock may be encountered beyond the 14 m cutback this could also be verified with subsurface investigations.

Our preliminary conclusion must be that sand movements at the southern sector would be the most straightforward and present the lowest risks, although as stressed in the above report, a detailed and systematic investigation of subsurface beachrock locations is required. Some hydrodynamic modelling may also be useful, although interpreting how projected model outputs relate to real weather to be experienced in the future is always as much art as science.

NECROPSY BASED ASSESSMENT OF REPRODUCTIVE BIOLOGY OF NESTING GREEN TURTLES AT RAINE ISLAND, 2016-2017 BREEDING SEASON

Colin J. LIMPUS[1], Owen I. COFFEE[2], Duncan J. LIMPUS[1], Mark READ[3] and Katharine ROBERTSON

1. Department of Environment and Heritage Protection
2. University of Queensland
3. Great Barrier Reef Marine Park Authority
4. Department of National Parks, Sport and Recreation

1. Introduction

When a female turtle is producing eggs, a scar (corpus luteum) approximately 15 mm in diameter is formed on the ovary for each mature follicle ovulated. The corpora lutea progressively heal over the following few years and shrink to form small white scars, corpora albicantia, approximately 1-2 mm in diameter on the surface of the ovaries (Hamann *et al*. 2003; Miller and Limpus, 2003). This scarring of the ovary is permanent for the remaining life of the turtle. Examination of an ovary to identify the presence or absence of corpora albicantia provides a definitive method for distinguishing turtles that have not breed in a previous season from those that have previously bred.

At nesting beaches, an examination of the ovaries of marine turtles can provide a measure of the recruitment rate of new females entering the annual breeding population. This is particularly applicable where marine turtles die from natural causes or are taken for food at nesting beaches.

For turtles taken on the nesting beach, examination of the oviducts for the presence or absence of oviducal eggs can also identify turtles that have successfully laid or have failed to lay an entire clutch of eggs before death. Hence, examination for presence/absence of oviducal eggs provides a definitive measure of nesting success.

A nesting female turtle expends a considerable amount of energy when she comes ashore and digs to make a nest. If she fails to successfully lay her eggs and returns to the sea, she can be expected to return for another attempt to lay the same clutch of eggs on the same night or within the next few nights. If she is repetitively unsuccessful in laying her eggs, she will deplete her stored fat reserves over a series of nights. Her capacity to replace those fat reserves during the nesting season is compromised by the female not feeding or feeding to a negligible amount during the inter-nesting periods. When a nesting female excessively reduces her stored fat reserves, she will commence to extract nutrients and energy from the mature ovarian follicles to support her immediate needs and to fuel her return migration back to her home foraging area. Once a mature follicle is being resorbed, i.e. undergoing atresia, it is no longer available for ovulation to make an egg. For a turtle that is repeatedly confronted with nesting habitat that impedes her capacity to successfully lay eggs, quantifying the proportion of large atretic follicles in an ovary can therefore provide a measure of the proportional reduction in remaining egg production for the turtle for that breeding season.

These methodologies have been introduced within the tool kit of the Department of Environment and Heritage Protection Queensland Turtle Conservation Project (Limpus *et al*. 2003, 2005) to provide a direct measure of:

- recruitment rate of new breeding females into the annual breeding population of marine turtles;
- nesting success of females ashore to lay their eggs;
- reduction in the number of clutches laid per female within a breeding season.

These are critical parameters with respect to the population dynamics of the species.

Necropsy based assessment of reproductive biology of nesting green turtles was reintroduced into the summer monitoring program of studies for the 2016-2017 breeding season at Raine island.

2. Methods

Freshly dead turtles on the nesting beach can provide a unique opportunity to investigate the specific reproductive biology and individual reproductive histories of *Chelonia mydas* at Raine Island.

When the study team was at Raine Island during the 2-11 November 2016 and 30 November – 5 December 2016 study periods, daily monitoring was conducted of dead or moribund turtles. Live moribund turtles were rescued as they were identified and returned to the sea following standard NPSR management at the island. As a result of this management intervention, the availability of freshly dead turtles is limited on current trips. The rescue protocols at the island this past breeding season resulted in the rescue of 43% of the recorded dead and moribund turtles on shore (Table 2). Given the success in rescuing most stranded turtles from the nights when the study teams are working in the island, freshly dead turtles were most likely to be available on the day the team arrived at the island for each trip.

Freshly dead turtles were necropsied to assess breeding condition as follows. The turtle carcass was rolled on its carapace and either the plastron was removed to expose the internal organs or an incision was made immediately anterior to a rear flipper to access the body cavity. A portion of the gonad and the associated oviduct was removed to the exterior of the turtle.

The ovary or portion of the ovary was spread on a flat surface such as the turtle’s plastron for photography (Figure 1). Each turtle was identified by its flipper tag number or by a printed specimen number with an “N” prefix. The printed specimen number was placed on the surface of the ovary for photography

Photographs were taken using a digital camera set at ISO 1,600 or higher. Images were checked for sharpness while ensuring that structures as small as 2mm in diameter were discernible. Eggs in left and/or right oviducts were counted.

If time permitted, the crop and stomach was examined for food content.

After being returned to the laboratory, the photographs were examined independently by two persons with advanced experience in identification of gonad morphology (Dr Colin Limpus, Duncan Limpus) to determine the presence of corpora albicantia and atretic and mature ovarian follicles. The number of mature ovarian follicles and mature sized atretic follicles were counted in one image of the gonad sample for each turtle.

Percentage estimates are summarised with ± 95% confidence limits.

3.1 Necropsy results

The gonads and oviducts of 14 freshly dead green turtles that had died while ashore on nesting crawls at Raine Island were examined from 16 attempted necropsies during the 2016-2017 nesting season (Table 2). Necropsies were discontinued with two turtles that were too decomposed to provide useful data.

Presence or absence of oviducal eggs was scored for 14 turtles. Only 3 (21 ± 21%) had completed oviposition (Table 2). The remainder carried oviducal eggs consistent with having laid no eggs while ashore for nesting during the night before they died or they had been disturbed, presumably by another turtle(s) before they had completed the laying of the full clutch of eggs. This represents a low nesting success for these nesting females.

Twelve (86 ± 18%) of the 14 ovaries examined contained mature follicles that had begun atresia (Table 2; Figure 2). For the turtles for which gonads were examined during 30 November – 5 December 2016, the mean proportion of large ovarian follicles that had commenced atresia per female was 21% (Table 2. n = 12, range = 0 – 65%). Based on an average female green turtle within the nGBR genetic stock laying on average of 6 clutches of 102 eggs per breeding season (Limpus *et al.* 2001), this level of mature follicle reduction would be equivalent to a reduction of at least one clutch of eggs for the entire season for these females, if they had not died at that time.

Considering female N86827 which was necropsied on 7 November 2016, this turtle had successfully laid the only clutch that she had ovulated for the season but had commenced resorption of 67% of her mature ovarian follicles. This turtle could only have produced one more clutch of eggs had she survived to lay additional eggs for the season. Assuming that this turtle was an average egg producer for this population, the loss of mature follicles due to atresia was equivalent to the loss of four clutches of for eggs for the season.

3.2 Recruitment to the nesting population

The 14 turtles examined by necropsy were scored for evidence of a past breeding history based on presence or absence of corpora albicantia in the ovaries. Both assessors scored each gonad identically. The one female with a past recorded breeding history based on tagging-recapture history was correctly scored via gonad examination as having bred in a previous breeding season. Corpora albicantia were found in all 14 females. This result, although from a small sample of the nesting population, suggests that there was a very low a recruitment rate, approaching 0**%** of new females into the 2016-2017 breeding population.

4. Discussion

The poor confidence limits associated with results from this summer's study are a direct outcome of the small sample size of turtles whose gonads were examined. During this past summer, necropsies were performed while an EHP team member was present to do necropsies and train NPSR staff up to 5 December. Although numerous dead turtles were available for necropsy (Table 1), staff were not assigned to conduct necropsy examinations of dead turtles during subsequent visits to Raine Island or Moulter Cay after 5 December 2016. Significant improvement of the precision of results obtained from these necropsy based studies in future years will require emphasis being placed in maximising the number of turtles taken for necropsy across multiple trips to these study sites.

While acknowledging the above limitation of this year's data, the data provides the following indications of poor population performance that should be considered.

Examination of oviducal eggs during necropsy of nesting females dying at Raine Island and adjacent islands provides a more comprehensive measure of nesting success than measures obtained by observation of nesting turtles on selected nights on the beaches. This is because external observations do not always detect turtles that lay only partial clutches and return to the sea carrying oviducal eggs. These remaining oviducal eggs may be dropped at sea. A nesting success of 21% should be viewed with concern given the consequences of repeated unsuccessful nesting attempts on resorption of mature ovarian follicles and hence reduction in egg production. This season's measured rate of atresia of mature ovarian follicles in the early nesting season (late November – early December) is equivalent to a reduction by one in overall clutch production per female per year and should be viewed with concern. There is a high probability that, with continued poor nesting success through the remainder of the breeding season, there would have been an overall reduction in clutch production within this nesting population by several clutches per female. If such a reduction in annual egg production is occurring, then the current green turtle nesting population of Raine Island and Moulter Cay will not be sustainable, irrespective of the hatching success of the eggs that are laid.

This high incidence of atresia among nesting females early in the breeding season has not been recorded at any Australian turtle rookery other than Raine Island. EHP studies that include gonad examination of nesting turtles via laparoscopy and/or ultrasonography include:

- Loggerhead turtles: Woongarra Coast over recent decades using laparoscope and ultrasound (EHP unpublished data).
- Green turtles: Heron Island during 1990s via laparoscopy and Raine Island (Limpus *et al*. 2003, 2005).
- Hawksbill turtles: Milman Island over recent decades using laparoscopy (Dobbs *et al*. 2007; Miller *et al*. 2008).
- Flatback turtles: Woongarra Coast over recent decades and Mapoon since 2005 using laparoscope and ultrasound (EHP unpublished data).
- Olive ridley turtles: Mapoon since 2005 and Tiwi Island in 2008 using laparoscope and ultrasound (EHP unpublished data; Limpus and Whiting, 2009).

These measures of nesting success and follicular resorption (atresia) provide a direct measure of management success of green turtle breeding on Raine Island that is independent of hatching success of eggs laid.

The current measures of breeding success obtained via gonad assessment are indicative of breeding success not being managed successfully at Raine Island. Therefore while management actions continue to address the poor breeding success of the Raine Island green turtle population, increased emphasis should be given to improving the quantification of these parameters that give definitive measures of nesting success and loss of egg production during annual monitoring of the nesting population by:

- significantly increasing the number of turtles being examined;
- increasing the sampling events to include early, mid and late breeding season;
- increasing the range of techniques applied to measure these parameters to include gonad examination via necropsy of freshly dead turtles and viewing of gonads of the nesting females via laparoscopic and/or ultrasound examinations (Limpus *et al*. 2003, 2005). There will be a deceasing quality of data obtained from these three approaches to assessing breeding via gonad examination.

In Queensland, EHP long term mark-recapture studies are demonstrating that adult green, loggerhead, flatback and hawksbill turtles can have breeding life expectancies of 40 or more years. Unless specifically monitored within these species with an extended reproductive life, the presence of numerous old age turtles in the population can mask the failure of recruitment of new young adults entering the breeding population. Recruitment rate into the breeding population is currently the only early warning metric available at the nesting beach for positive assessment of whether or not past management actions are contributing to an increasing nesting population.

A low annual recruitment of new adult females into the breeding population within the northern GBR green stock as identified by females with no corpora albicantia has been of concern in recent decades (Limpus *et al*. 2003; Limpus *et al*. 2005) and is a continuing issue. This provides a measure of the success in management of the nGBR green turtle stock over recent decades across its total population distribution and throughout all life history stages. Low annual recruitment to the breeding population indicates that past and current management practices for this stock have not been successful.

If a large population is to be maintained for the nGBR green turtle stock, it is essential that management is not only directed to restoring successful breeding rates at the nesting beaches, especially at Raine Island and Moulter Cay, but is also needs to be directed to increasing survival/reducing mortality from human activities for these turtles throughout their dispersed foraging range.

5. References

Dobbs, K. A., Miller, J. D., and Landry, A. M. Jr. (2007). Laparoscopy of nesting hawksbill turtles, *Eretmochelys imbricata*, at Milman Island, northern Great Barrier Reef, Australia.

Chelonian Conservation and Biology **6**, 270-274.

Hamann, M., Limpus, C. J., and Owens, D. W. (2003). Reproductive cycles of males and females. In 'The Biology of Sea Turtles. Volume II'. (P. L. Lutz, J. A. Muzick, and J. WynekenEds.) pp. 135-161. (CRC Press: Boca Raton.)

Limpus, C. J., Carter, D., and Hamann, M. (2001). The green turtle, *Chelonia mydas*, in Queensland: the Bramble Cay rookery in the 1979-1980 breeding season. *Chelonian Conservation and Biology* **4**, 34-46.

Limpus, C. J., Limpus, D. J., Arthur, K. E., and Parmenter, C. J. (2005). Monitoring green turtle population dynamics in Shoalwater Bay: 2000-2004. *GBRMPA Research Publication* **83**, 1-51.

Limpus, C. J., Miller, J. D., Parmenter, C. J., and Limpus, D. J. (2003). The green turtle, *Chelonia mydas*, population of Raine Island and the northern Great Barrier Reef: 1843-2001. *Memoirs Queensland Museum* **49**, 349-440.

Limpus, C. and Whiting, S. (2009). Estimating breeding recruitment rates of olive ridley turtles in Northern Australia. *Report to Natural Heritage Trust* 1-22.

Miller, J. D. and Limpus, C. J. (2003). Ontogeny of marine turtle gonads. In 'The Biology of Sea Turtles. Volume II'. (P. L. Lutz, J. A. Muzick, and J. WynekenEds.) pp. 199-224. (CRC Press: Boca Raton.)

Miller, J. D., Limpus, C. J., and Bell, I. P. (2008). The nesting biology of *Eretmochelys imbricata* in the northern Great Barrier Reef. In “Australian hawksbill turtle population dynamics project.” Eds Limpus, C. J. and Miller, J. D. Pp. 41-93. (Queensland Environmental Protection Agency: Brisbane)

Table 1. Summary of dead or moribund turtles encountered at Raine Island and Moulter Cay during study trips to the islands during the 2016-2017 nesting season. US denotes an unsuccessful necropsy. * denotes incomplete count.

Date	Number of	dead and	moribund	Turtles
	**Total for night**	**Dead turtles**	**Rescued and released to sea**	**Necropsied**
**Raine Island**				
2016, 2 Nov	13 (on arrival)	10	3	1
3 Nov	6	1	5	
4 Nov	2	0	2	
5 Nov	5	0	5	
7 Nov	3	1	2	1
8 Nov	3	0	3	
9 Nov	4	0	4	
10 Nov	4	0	4	
11 Nov	2	0	2	
**No. 8 Sandbank**				
2016, 11 Nov	Nil (on arrival)	0	0	
**Raine Island**				
2016, 30 Nov	49 (on arrival)	42	7	10
1 Dec	15	7	8	
2 Dec	10	2	8	1 + 1 US
3 Dec	8	2	6	
4 Dec	14	3	11	1 + 1 US
5 Dec	13	3	10	
**Raine Island**				
2016, 8 Dec	7	3	4	
**Raine Island**				
10 Dec	26	21	5	
11 Dec	5	0	5	

**Moulter Cay**				
2016, 12 Dec	44 (on arrival)	42	2	
13 Dec	3	0	3	
14 Dec	10	8	2	
**Raine Island**				
2017, 29 Jan	66	54	12	
30 Jan	6	2	4	
31 Jan	3	0	3	
1 Feb	9	0	9	
2 Feb	3	0	3	
3 Feb	4	0	4	
4 Feb	6	0	6	
5 Feb*	2	0 *	2	
**Raine Island**				
9 Feb*	9	- *	9	
**Raine Island**				
11 Feb	10	3	7	
12 Feb	3	0	3	
**Moulter Cay**				
2017, 13 Feb	13 (on arrival)	13	0	
14 Feb	4	0	4	
**TOTAL RECORDS**	**384**	**217**	**167**	**14**

Table 2. Summary of results of necropsy of freshly dead female *Chelonia mydas* that had died on the beach platform following nesting attempts during a previous night on Raine Island, 2 November - 04 December 2016. 'Yes' denotes presence; 'Nil' denotes absence; "-" denotes not recorded.

Date 2016	Tag no.	CCL (cm)	Oviducal eggs		Corpora albicantia	Mature follicles in atresia	Notes
			Left	right			
02 Nov	N86826	107.8	39	44	Yes	Nil (0/5)	• Spent ovary;<br>• Nil stomach content
07 Nov	N86827	102.6	0	0	Yes	Yes (46/69)	• Died after only ovulating 1 clutch which was

							successfully laid on this night.
30 Nov	N86828	98.7	52	-	Yes	Yes (7/22)	
30 Nov	N86829	105.7	-	42	Yes	Nil (0/33)	
30 Nov	N86830	108.8	69	-	Yes	Yes (7/42)	
30 Nov	N86831	103.3	-	50	Yes	Yes (7/42)	
30 Nov	N86832	102.3	0	-	Yes	Yes (6/35)	
30 Nov	N86833	99.8	-	46	Yes	Yes (4/21)	
30 Nov	N86834	103.3	-	50	Yes	Yes (26/40)	
30 Nov	N86835	109.7	-	45	Yes	Yes (8/37)	
30 Nov	QA8291<br>QA66030<br>N86836	114.0	-	0	Yes	Yes (8/38	• Nesting Raine Is. Nov 2009.<br>• Agreement of gonad & capture data
30 Nov	QA74806<br>N86837	111.1	-	27	Yes	Yes (3/48)	• First tagged at Raine Is. Dec 2016
02 Dec	N86839	106.1	35	-	Yes	Yes (4/29)	
04 Dec	N86840	102.3	46	-	Yes	Yes (5/31)	

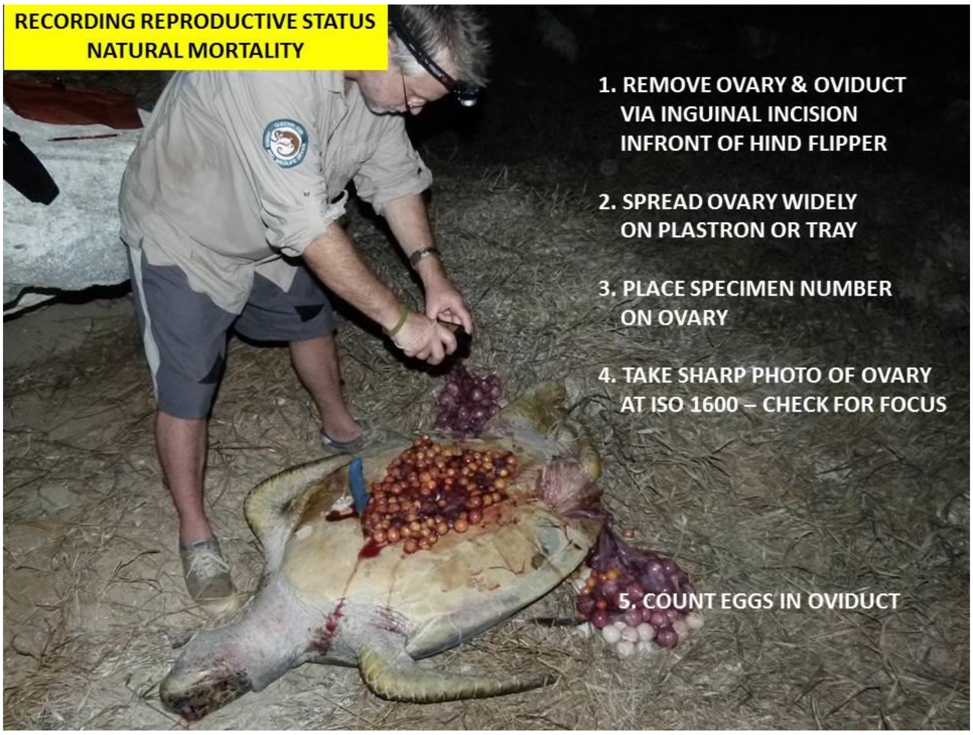

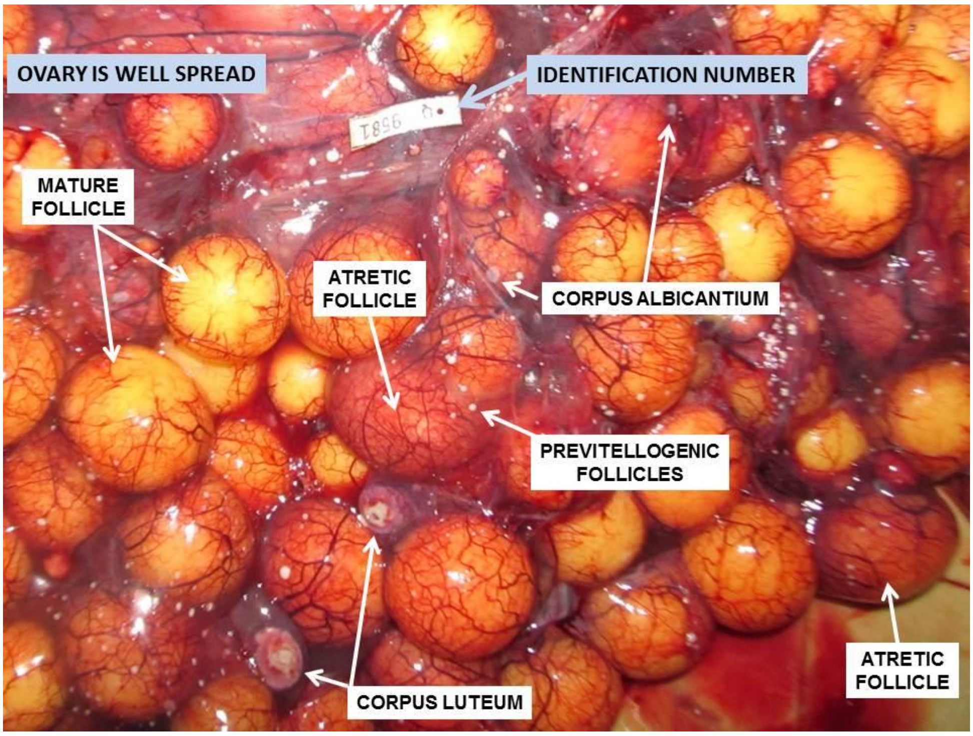

Figure 1. Simplified instructions issued to team members performing the necropsies of dead green turtles at Raine Island and taking photographs of gonads.

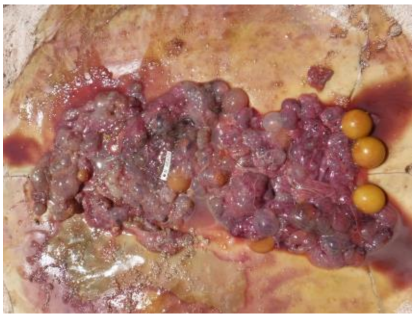

N86826

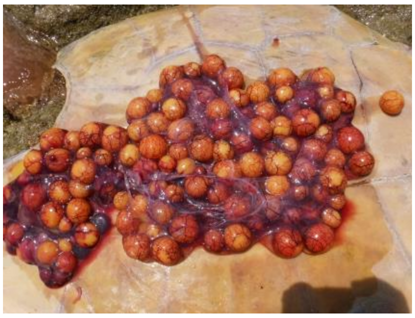

N86827

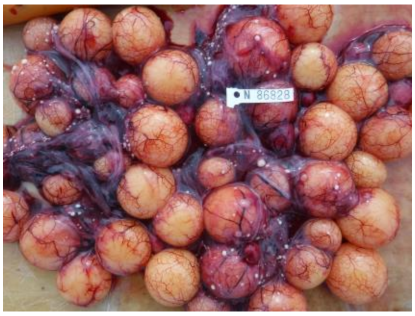

N86828

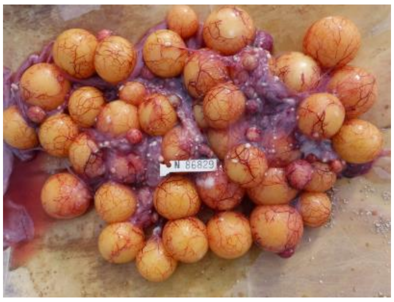

N86829

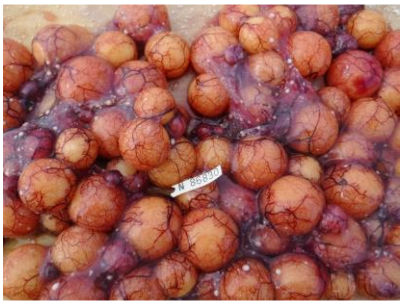

N86830

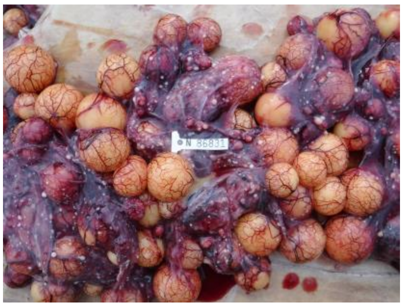

N86831

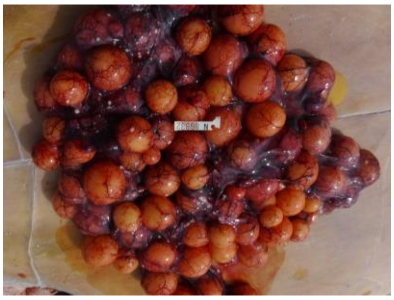

N86832

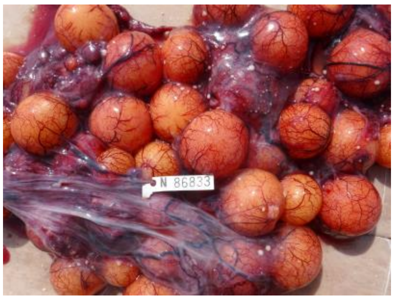

N86833

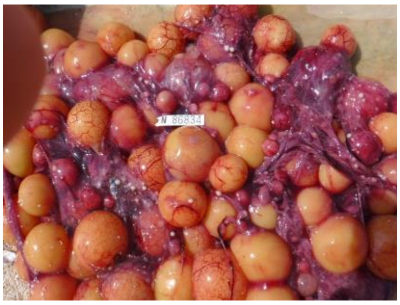

N86834

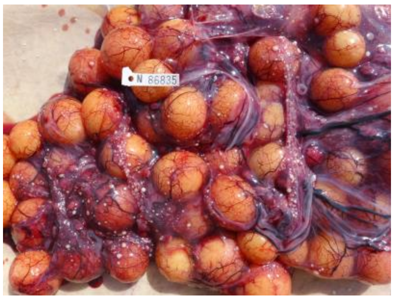

N86835

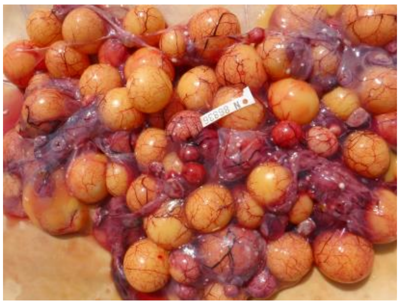

N86836 = QA8291

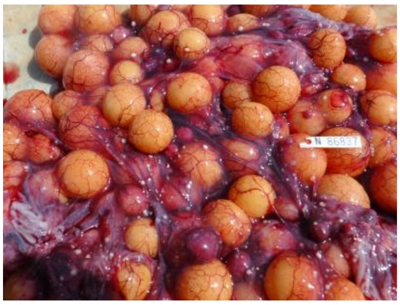

N86837 = QA34806

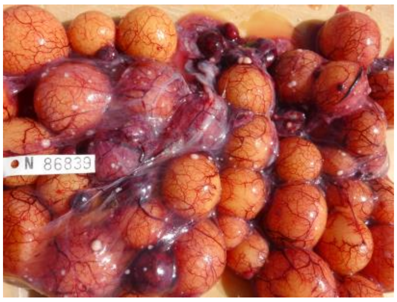

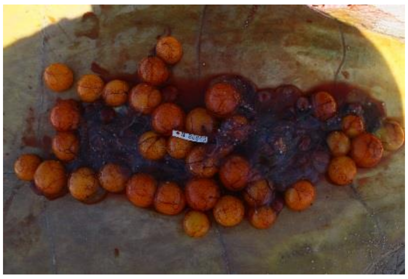

N86839 **N86840**

Figure 2. Images of portions of the gonads of 14 breeding green turtles necropsied at Raine Island, 2016-2017 breeding season and identified by specimen and tag numbers (Table 2).

Assessment of the potential for Fastloc GPS satellite telemetry to quantify the breeding success, habitat use and effectiveness of habit restoration for green turtles nesting at Raine Island: a report on use of satellite telemetry at Raine Island, 2015 and 2016 breeding seasons.

Taka Shimada[1,2], Mark Hamann[1] and Colin J. Limpus[2]

1. College of Science and Engineering, James Cook University
2. Conservation and Biodiversity Operations, Department of Environment and Heritage Protection

Introduction

Protecting biodiversity, in particular of the Great Barrier Reef, is a Science and Research Priority for Queensland (Office of the Queensland Chief Scientist 2015). Marine turtles are considered important as potential ecosystem engineers, and thus it is considered to be critical to conserve them to maintain biodiversity (Coleman and Williams 2002; Moran and Bjorndal 2007). They are also one of the main wildlife species that attract tourists to the Queensland billion dollars tourism (Queensland Government 2016). However, all sea turtle species are currently of listed as threatened due to past and current human activities (Nature Conservation Act, Environmental Protection and Biodiversity Conservation Act; Wallace et al. 2011; IUCN 2017). In order to conserve marine turtles, it is necessary to understand the current status of the populations and threats to which they are exposed. Conservation management will benefit from regular revision in response to the increasing understanding of their complex biology.

Green turtles breeding in the northern Great Barrier Reef (nGBR) are an independent genetic stock of profound conservation concern (FitzSimmons and Limpus, 2014; Chaloupka *et al*. 2008). The largest aggregation of the nesting green turtles occurs in Raine Island (Limpus *et al*. 2003), and as such it is critical to understand the trends and population dynamics of this nesting population. Investigation of these turtles based on tagging studies have been long been used to describe nesting behaviour and estimate population size of the Raine Island nesting turtles using data collected by surveys on the island and in the waters around the island. It was not possible with these studies to quantify key demographic parameters such as the number of clutches laid per female per breeding season and adult survivorship. In addition, a key assumption of the population estimation methods is that the turtles remain in the vicinity of the island during the nesting season. However, flipper tag recovery records indicate that some turtles may not stay close to Raine Island, instead spending some time elsewhere between successive nesting occasions where they can't be sampled using the existing techniques. The potential departure from the area adjacent to Raine Island indicates that the assumption for the current methods for population estimation may be violated, and subsequently the estimates may not be reliable. Clearly the movements of turtles within the inter-nesting period need to be taken into account to accurately estimate the population size of green turtles nesting at Raine Island.

Until recent years, no methods had been available for tracking marine turtles at sea with sufficient accuracy to adequately resolve their positions within the scale of Raine Island beach and its associated reef and the surrounding in-water survey area. A location resolution of <100 m is required to achieve this. High-resolution tracking is now possible using the new Fastloc GPS technology (FGPS).

In the present study, we used a small number of FGPS tags to demonstrate their capacity for collecting accurate tracking data from female green turtles nesting at Raine Island and providing results not available from previous studies. These tags have the capacity to record the timing and location of a turtles nesting activities on the island and hence provide a minimum estimate of the number of clutches laid within a nesting season by individual females and a measure of their nesting success (proportion of visits to the island that result in egg laying). The satellite telemetry data can be analysed to estimate the probability of a turtle's presence and absence within the in-water survey area where mark-recapture studies occur for estimation of population size. These availability estimates have the potential to improve the estimation of turtle population size within the inter-nesting habitat at

Raine Island, and will thus allow for more accurate estimate of the past and present population sizes within the nGBR green turtle stock. Once the tagged turtles cease nesting for the season, the FGPS data also allows for precise definition of migratory pathways, locations of their foraging habitats and the geographical extent of these foraging areas.

This report primarily focus on the habitat use of female green turtles at the Raine Island region during their inter-nesting periods and provides biological insights inferred from satellite tracking data, which have never been recorded for the Raine Island turtles using other conventional methods.

Material and methods

Analytical tools and geographical data

R software was used for data preparation and analyses (R Core Team 2017). Figures were created using the R packages ggplot2 (Wickham 2009), ggmap (Kahle and Wickham 2013) and ggsn (Baquero 2017). ArcGIS 10.1 software was used to outline geographical features (e.g. boundaries of islands, cliff and reefs) from geo-coordinated satellite imagery. IKONOS satellite imagery at resolution of 0.8 m (courtesy of Dr. J. Dawson) or high-resolution satellite images available from Google online sources were used.

Study turtles

Adult female green turtles were captured during their nesting attempts at Raine Island in December 2015 (n = 2) and November 2016 (n = 3). Given that the peak nesting density occurs during December and January at Raine Island (Limpus et al. 2003), the turtles captured in 2016 were presumed to be near the commencement of hat nesting season, and those in 2015 were likely at the middle of their nesting season. Each turtle had been sighted at Raine Island at least once during the previous surveys (Table 1). K74859 had also been recorded once at her foraging habitat in the Coombe Reef (latitude -14.425, longitude 144.947) prior to have been recorded nesting at Raine Island. The curved carapace length (CCL) of the turtles ranged from 98.0 to 109.9 cm with a median of 103.2 cm. One turtle (I759) was missing about a half of its right hind flipper (Fig. 1a). The other turtles did not have significant flipper damage.

Each turtle was deployed with an Argos-linked Fastloc GPS tag on the carapace (Table 1, Fig. 1b). The two turtles captured in the 2015 trip were taken on a small barge, which was secured to the Reef Ranger during attachment of the tags (Fig. 1c). During the 2016 trip, the turtles (n = 3) were immobilised using the Gyuris and Limpus (1986) method and kept in a timber enclosure on the beach close to areas where they were captured while the tags were attached (Fig. 1d). The turtles were released to the nearby water on the following morning (Fig. 1e). The satellite tags were configured to obtain a GPS location every 30 minutes using the Fastloc technology.

Data acquisition and pre-processing

The raw GPS data were downloaded via Argos satellite system or a USB link directly from the tag to computer (Fig. 1f), and then decoded into GPS locations using the Wildlife Computers' web-based data processor. The tags also used Argos satellite system for location estimation. To maximise the sample size, we merged the GPS fixes (>30 GPS satellites and residual error < 30) with high-quality Argos fixes (Location Classes 3, 2, 1). The satellite telemetry data were then screened by a data-driven filter (Shimada *et al*. 2012; Shimada *et al*. 2016b) using the R package SDLfilter (Shimada 2017). We also removed location fixes above the cliff of Raine Island because no turtle was The mean error of GPS fixes treated with the data-driven filter is estimated to be <50 m (Shimada *et al*. 2012).

Classification of nesting events and attempts

The satellite tag also provided a time and a location if the turtle consecutively stayed above the water for more than 10 minutes (a "haulout" event hereafter). Additionally, two tags deployed in 2015 (133764, 133765) were set up to record the duration of the "haulout" events. We used a combination of satellite-derived locations and haulout data to estimate the timings of the nesting events/attempts of the tracked turtles.

When the tracking data suggested a turtle was on the beach at night, we estimated the nesting activity was

- "unsuccessful" on the night if a potential nesting activity was also recorded during the nights for the following eight days or,
- "successful" on the night if a potential nesting activity was not recorded during the following eight days.

The threshold days (i.e. 8 days) was chosen based on known biological information of nesting females. In general, a female sea turtle requires 9-10 days of clutch preparation period after ovulation (Hamann et al. 2003), and the minimum renesting interval appears 9 days for this population of green turtles nesting at Bramble Cay (Limpus *et al*. 2001).

Detailed analysis for inter-nesting behaviour relating to boat survey

Inter-nesting habitat areas of the tracked turtles were represented by the utilisation distributions (UDs). We estimated a UD using a movement-based kernel density estimator based on a biased random bridge (Benhamou 2011). The parameters for the UD estimation were adopted from Shimada *et al*. (2016a). Raine Island and Moulter Cay were used as boundaries for UD estimation. We defined the areas containing 95% of the UD as the inter-nesting home range areas, and 50% of the UD as the inter-nesting core areas. The R package adehabitatHR (Calenge 2006, 2015) was used to estimate
UDs, and to calculate the size of the home range and core areas.

In 2015 and 2016, in-water turtle count survey was conducted on eight separate days using the Darter II research boat. The on-board GPS receiver recorded the locations of the boat (n = 506) during the survey exercises at an interval of five minutes. The distances between the reef edge and the boat locations were up to 347.7 m away from the reef and up to 2.6 m on the reef. Therefore we considered areas <350 m outward and <3 m inward from the reef edge as the boundaries of the boat survey (Fig. 2).

To assess turtle habitat use in relation to the area of boat survey, we first estimated the straight distances between turtle locations and the reef edge. For this analysis, we only used the turtle locations obtained during the daytime (8am to 5pm) to correspond to the times of the boat survey. The distance calculation and related spatial processing were executed using the R packages raster (Hijmans 2016), rgdal (Bivand *et al*. 2017), and rgeos (Bivand and Rundel 2017).

We modelled the distances of turtles from the reef edge using Bayesian generalised linear mixed model (Bayesian GLMM). The dependent variable was the number of days following their nesting events or attempts at Raine Island. We log-transformed the response variable and fitted the model with a Gaussian distribution error structure using an identity link function. Each turtle was treated as a random effect to allow for within-turtle correlation. Three chains were used in the Markov Chain Monte Carlo process with a burn-in of 2,000 samples and a thinning rate of 10, generating 800 iterations for each posterior distribution. We verified there is not apparent issue with autocorrelation and mixing of the chains. The Bayesian analysis and model diagnosis were conducted using the R packages rstanarm (Stan Development Team 2016b), rstan (Stan Development Team 2016a) and broom (Robinson 2017).

Migration and foraging periods

We defined a post-breeding migration of a turtle starts at the first in-water location following the last nesting event, and ends at the first location within its foraging habitat, which are represented by aggregation of fixes at the end of the migration path. For each turtle, the duration of migration was estimated as the time difference between the first and last location of migration track. We also estimated migration distance in two different ways, namely beeline distance and travelling distance. Beeline distance is the straight-line distance between the first and last location of

the track, and travelling distance is the sum of distances between successive locations along the track. Finally, minimum travelling speed was calculated using the duration of migration and travelling distance.

Power analysis

We investigated the probability of Type II error which the present study may have. The null hypothesis was derived from the proportion of turtles that departed from the area around Raine Island following their nesting events or attempts. The alternative hypothesis was set to be half of the probability for the null hypothesis. We used the R package pwr to perform the power analysis.

Results

Tracking data

The green turtles were tracked for periods ranging from 67 to 124 days (median = 123 days). Satellite telemetry data collected up to 8 March 2017 was used but the tags deployed in November 2016 (n = 3) were still in operation at that time. Four turtles were tracked at their nesting/inter-nesting habitats, during the subsequent post-nesting migration, and in their foraging habitats. Another turtle (T90143) was only tracked at her nesting/inter-nesting habitats because transmission from the tag ceased before she commenced her post-nesting migration.

Nesting events and attempts

The satellite-derived location and haulout data identified a total of 31 nights of successful nesting events and 51 nights of unsuccessful nesting attempts from the five turtles (Table 2). A total of two or six presumed nesting events were detected from the two turtles tracked in 2015/2016, and seven or eight from the three turtles tracked in 2016/2017 (Table 3). The rate of nesting success ranged 0.25 to 0.60 with a median at 0.38 (Table 3). The lowest nesting success (0.25) was observed by an undamaged turtle tracked in 2015 (I22154), followed by 0.26 observed from a turtle tracked in 2016 (I759) whose right hind flipper was half missing (Table 3, Fig. 1a).

The turtles were also observed on Raine Island following satellite tag deployment. Nesting events were observed in two occasions and non-nesting activities (e.g. wondering, returning to sea) were observed in five occasions. Estimation made by satellite telemetry and field observation were agreeable in 5 out of 7 occasions (2 nesting and 3 non-nesting activities). For the other two occasions, satellite data suggested successful nesting but nesting event was not observed from the turtles on the beach. These two turtles appeared on their way back to the sea when they were found on the beach (Shimada and Robertson, field observations). These two turtles may have been on the beach for hours before they were seen by the observers.

The duration of each nesting activities (successful or unsuccessful), recorded for two turtles, ranged from 1.1 to 10.7 hours at a median of 3.8 hours (Table 2). The duration tended to be longer when the nesting attempt was successful, ranging from 1.9 to 10.7 hours at a median of 4.9 hours. Note that any attempts less than 10 minutes on the beach were not detected by the satellite tags due to the settings used in the present study (see Materials and methods - *Classification of nesting events and attempts*).

The period between a successful nesting event and the successive nesting attempt (i.e. inter-nesting interval) ranged from 9 to 13 days (median = 11 days). Each turtle often attempted nesting over multiple nights before successfully laid a clutch. The number of nights between the first nesting attempt and a subsequent successful nesting ranged from 0 to 14 days (median = 1 days). The longest sequence of consecutive nesting attempts was observed with I759 whose right hind flipper was partially missing (Fig. 1a). For all tracked turtles combined.

I759 nested and attempted nesting both in Raine Island and Moulter Cay (Fig. 3a, b). In particular, during her continuous attempts of nesting over 14 days, she visited Raine Island for the first three nights, then a minimum of 6 nights over 8 days at Moulter Cay, and back to Raine Island for other three nights before the apparent success in

nesting on 30/11/2016. Despite multiple failure in nesting attempts, she continued returning to the northwest side of Raine Island, although her locations on Moulter Cay were more widely spread. The other turtles nested or attempted nesting only at Raine Island during our tracking study. Two turtles (T7159, T90143) predominantly used northwest side of the island, where they were originally captured for satellite-tag deployment (Fig. 3e, f). In contrast, the other two turtles (I22154, K74859) used wider range of the beach (Fig. 3c, d). There doesn't seem an apparent pattern in sections of the beach where nesting occurred or failed (Fig. 3).

Inter-nesting habitat use with relation to boat survey area

There are large variation in the inter-nesting habitat use of the tracked turtles. The home range areas (i.e. 95% UDs) ranged from 3.3 to 146.4 km^2 with a median of 6.5 km^2, and the core areas (i.e. 50% UDs) ranged from 0.3 to 7.0 km^2 with a median of 1.7 km^2 (Table 4). Three turtles (I22154, K74859, T7159), residents hereafter, mostly used the areas immediately around Raine Island during their inter-nesting periods (Fig. 4c, d, e). In particular, the residents mostly remained around the edge of the reef surrounding Raine Island (Fig. 5). The other two turtles (I759, T90143), nomads hereafter, used much wider range of areas, with their core habitats in reefs both near and distant to Raine Island (Fig. 4a, b). The nomads stayed around Raine Island for less than 50% of their inter-nesting periods (Fig. 5).

A turtle's daytime locations changed over days following the nesting events or attempts at Raine Island (Fig. 6). Nonetheless, the resident turtles mostly stayed within or near the boat survey area throughout their inter-nesting period (Fig. 6b, c, d). In contrast, the nomads appeared to spend much less time around the boat survey area and stayed longer elsewhere (Fig. 6a, e). Our analysis with Bayesian GLMM estimated higher chances of a turtle's presence near Raine Island in the first and second days following their nesting events/attempts at Raine Island, than days later on (Fig. 7). Yet, the probability of the turtles present within the boat survey area is consistently low throughout the inter-nesting period (i.e. <0.42), with the highest probability (0.41) occurring on the day immediately after successful and unsuccessful nesting events at Raine Island (Table 5).

Post-nesting behaviour

The tracked turtles departed Raine Island as early as the 23rd of December, and as late as the 1st of February (Table 6). The post-nesting migration took 4 to 15 days (median = 12 days) and a total of 21 to 150 locations were used to reconstruct their migration paths (Fig. 8). The beeline distance of the migration track ranged from 143 to 324 km (median = 251 km). The minimum distance each turtle travelled (i.e. travelling distance) ranged from 166 to 440 km (median = 280 km). The travelling speed ranged from 0.9 to 1.6 km/h (median = 1.2 km/h). I22154 travelled to the north and settled in Dollar Reef south of Badu and Moa Islands in Torres Straits (Fig. 8). The other turtles travelled to the south and settled in at Jubilee Reef west of Night Island (I759), Corbett Reef north of Princess Charlotte Bay (T7159), or Coombe Reef (K74859; where she had been previously captured by the rodeo method) (Table 1). Foraging habitat use of the turtles was not analysed in the present study as the tracking data were not completed as of the preparation of this report (see Results - tracking data).

Sample size

The null hypothesis of the power test was set 0.4 based on the proportion of nomads among the tracked turtles (i.e. 2 out of 5 turtles were nomads), and 0.2 for the alternative hypothesis. The power analysis detected substantially low power (0.17) for the present study, indicating the sample size (n = 5) was too small. To increase the confidence of the parameters estimated in this study, a larger sample size is required. The high power (0.80), which is desirable for many biological studies (Whitlock and Schluter 2009), is likely achieved for our study with the sample size of ≥40 turtles (Fig. 9).

Discussion

This study demonstrated insights regarding information that can be obtained by FGPS satellite telemetry. These results are highly applicable to conservation and other studies such as modelling population sizes. Interpretation and inferences that can be derived from the tracking data are provided below. However, due to the detected low power as a result of small sample size (i.e. n = 5), the results of the present study needs to be treated with caution, or rather as an illustration of what can be achieved using FGPS satellite tracking data. Thus, it is highly desirable to increase the sample size (e.g. ≥40 turtles) so that more reliable parameters can be estimated.

The study has demonstrated how satellite telemetry data can identify the number of successful and unsuccessful nesting events for each turtle, even across multiple nesting sites (e.g. Raine Island and Moulter Cay). Each turtle tracked in 2016/2017 presumably laid 7 or 8 clutches during the season. These numbers of clutches are greater than the mean number of clutches per season of 6.2 for green turtles nesting at Bramble Cay (Limpus *et al*. 2001). The other turtles tracked in 2015/2016 (I22154, T90143) laid fewer clutches during the tracking period (2 and 6 respectively). Given the time of tag deployment and the number of presumed nesting events, the 2016/2017 turtles, and possibly T90143, were likely deployed with satellite tags close to the beginning of their respective nesting seasons. In contrast, I22154 may have commenced her nesting activity much earlier than the commencement of her tracked behaviour.

Our data also highlighted the application of satellite telemetry data to estimate inter-nesting and re-nesting intervals. The re-nesting interval estimated using our satellite telemetry data (10 to 25 days with a median of 12 days) was similar to those (9-19 days with a mean at 12.4 days) recorded for the green turtles of same population nesting at Bramble Cay (Limpus *et al*. 2001). However another potential issue when estimates are made using flipper-tag recovery data has emerged: I759 was at Raine Island three days from 17/11 to 19/11, and then re-appeared at Raine Island from 28/11 to 1/12. Since turtles are physiologically capable of nesting every 9 days (Hamann *et al*. 2003), if the turtle was seen on the beach on both occasions but never seen in-between, it could be assumed that she laid two clutches during these two periods. However satellite telemetry data detected her consecutive nesting attempts at Moulter Cay during her absence at Raine Island (20/11 to 27/11), indicating she did not lay a clutch on the 19/11 but only laid on 1/12. This is the first time that detailed inter-nesting parameters, such as re-nesting intervals and number of nesting events/attempts, have been recorded for turtles at Raine Island (Limpus *et al*. 2003).

Satellite telemetry data also detected a low rate of nesting success (38%). Although the nesting success of I759 was likely hindered by the damaged flipper, the other turtles also showed low nesting success (Table 3). Other potential issues causing a reduction in nesting success at Raine Island have been identified: dryness of the sand, encountering buried obstructions to digging and disturbance by other turtles (Limpus *et al*. 2003). All of those can cause reduced nesting success and a turtle will continue attempting nesting, often over multiple nights, until it succeeds. Every attempt results in consumption of additional energy. As nesting turtles generally do not feed during nesting season, once they use up their reserve of fat, they start resorbing the yolk from ovarian follicles as a source of energy. Consequently, reduced nesting success may lead to reduced number of clutches a female can lay and subsequently affect their reproduction fitness. However, this impact has not yet been quantified.

The extent to which repeated unsuccessful nesting attempts may lead to reduced seasonal reproductive output can be tested using a variety of approaches. The most direct method would be to use visual examination of gonads to assess number of clutches laid, the number of clutches lost through atresia of mature follicles and the number of mature ovarian follicles still available for ovulation. Alternatively, estimate energy expenditure of the turtles during each phase of reproduction (i.e. egg production, nesting activity, interesting activity, migration) with GPS tracking and Time-depth recorders (TDR) for identifying each phase of breeding activity (i.e. nesting and inter-nesting activities, migration) . Energy budget for each activity phase can be estimated using the published data: Prange and Jackson (1976) for nesting activities, Enstipp et al. (2011) and Halsey *et al*. (2011) for in-water activities (i.e. inter-nesting, migration), and Bouchard and Bjorndal (2000) for egg production. Blood sample may also be needed as indicators of physiological condition. Finally their reproductive fitness may be inferred by comparing the energy expenditure with those of healthy population. The outcome of these studies will have important conservation implications. Managers can prioritise their conservation effort to particular phases of turtles’ activity to increase their reproductive output.

This study has identified potential error in the current boat survey method and assumptions used for modelling the size of the Raine Island nesting population. The probability of turtles' presence within the survey area measured in this study is much lower than previously assumed. This is because the inter-nesting habitats of turtles often lay beyond the in-water survey area used for Petersen Index estimation of the population size, either on or off the reef surrounding Raine Island. Prior to about 2014, the boat survey area included the reef flat (Limpus *et al*. 2003). The probability of a turtle's presence on the reef flat and the adjacent area would have been 0.61 on the first day following their nesting activities. However, by excluding the reef flat from the survey area (i.e. current survey area), we estimated the probability has decreased to 0.41. This difference needs to be taken into account when estimating population using the boat survey data. Alternatively, the recent use of UAV (unmanned aerial vehicles) may compensate for this bias by detecting the turtles outside of the current survey area at short range on and off the reef flat.

However it is beyond the capacity of the survey by boat or UAV to detect turtles many kilometres away from Raine Island reef. This can be problematic because the turtles likely spend substantial time away from the reef surrounding Raine Island. For example, two of the tracked turtles spent nearly 50% or more time in areas 10 km or more away from Raine Island Reef (Fig. 5). The probability estimates that can be obtained using satellite telemetry data will enable correction of this bias that is occurring in the current turtle counting data, and thus improve the population estimation for green turtles nesting at Raine Island.

Another potential factor that may influence population size modelling is the effect of water depth on turtle visibility. Using time-depth recorders (TDRs) deployed on five turtles, Bell *et al*. (2009) found Raine Island turtles typically used the upper 6.8 m of the water column during their inter-nesting periods. This diving behaviour may decrease the ability of observers to recognise the turtles given that turtles are typically not detectable at water deeper than 2.5 m even in clear waters during manned aerial surveys (Fuentes *et al*. 2015). Population estimates using animal count data with marine species can be greatly improved by incorporating diving patterns of the animals (Hagihara *et al*. 2014; Fuentes *et al*. 2015). It is recommended that this detection probability at depth needs to be re-assessed using cameras mounted under the survey vessel and on low level UAV. Studies of detailed diving behaviour, together with more comprehensive satellite telemetry studies, would be warranted to further improve the population estimates of nGBR green turtles.

One of the tracked turtles (K74859) demonstrated over 10 years of site fidelity both to her nesting beach (Raine Island) and to her foraging habitat (i.e. Coombe Reef). This behaviour aligns to the movements of southern GBR green turtle populations and other sea turtle species (Limpus *et al*. 1992; Limpus and Limpus 2001; Plotkin 2003; Schofield *et al*. 2010). The fidelity to home habitat is known to persist even after human-induced displacement for several species of sea turtles at different locations (Limpus 1992; Avens *et al*. 2003; Shimada *et al.* 2016b). The accumulating evidence of their site fidelity behaviour confirms the importance of protecting their existing foraging habitats to conserve those threatened species.

With an increased sample size of tracked turtles, FGPS satellite telemetry data from turtles nesting at Raine Island has the potential to provide a rigorous assessment of the success of beach restoration in restoring high nesting success within the trial restoration areas and across the island as a whole. These data will also quantify unsuccessful nesting effort and the number of clutches laid per female for a breeding season, which are critical data for modelling the population dynamics of the species. Additionally integration of studies on their dive behaviour, which can be incorporated into the satellite telemetry studies, will enhance capacity to improve the estimation of the population in the interesting habitat surrounding Raine Island.

The present study provides a guide to potential results which satellite telemetry study can achieve with an adequate sample size of tracked turtles (e.g. ≤40 turtles).

References

Avens L, Braun-McNeill J, Epperly S, Lohmann KJ (2003) Site fidelity and homing behavior in juvenile loggerhead sea turtles (*Caretta caretta*). Mar Biol 143:211-220 doi:http://dx.doi.org/10.1007/s00227-003-1085-9
Baquero OS (2017) ggsn: north symbols and scale bars for maps created with 'ggplot2' or 'ggmap'. R package v. 0.4.0. https://CRAN.R-project.org/package=ggsn. Accessed 11 April 2017
Bell IP, Seymour J, Fitzpatrick R, Hogarth J (2009) Inter-nesting Dive and Surface Behaviour of Green Turtles, *Chelonia mydas*, at Raine Island, Northern Great Barrier Reef. Mar Turtle Newsl 125
Benhamou S (2011) Dynamic approach to space and habitat use based on biased random bridges. PLoS One 6:e14592 doi:http://dx.doi.org/10.1371/journal.pone.0014592
Bivand R, Keitt T, Rowlingson B (2017) rgdal: bindings for the geospatial data abstraction library. R package v. 1.2-6. https://CRAN.R-project.org/package=rgdal. Accessed 11 April 2017
Bivand R, Rundel C (2017) rgeos: interface to geometry engine - open source (GEOS). R package v. 0.3-23. https://CRAN.R-project.org/package=rgeos. Accessed 11 April 2017
Bouchard SS, Bjorndal KA (2000) Sea turtles as biological transporters of nutrients and energy from marine to terrestrial ecosystems. Ecology 81:2305-2313
Calenge C (2006) The package “adehabitat” for the R software: a tool for the analysis of space and habitat use by animals. Ecol Modell 197:516-519 doi:http://dx.doi.org/10.1016/j.ecolmodel.2006.03.017
Calenge C (2015) adehabitatHR: home range estimation. R package v. 0.4.14. http://CRAN.R-project.org/package=adehabitatHR. Accessed 19 September 2015
Chaloupka M et al. (2008) Encouraging outlook for recovery of a once severely exploited marine megaherbivore. Global Ecol Biogeogr 17:297-304 doi:https://dx.doi.org/10.1111/j.1466-8238.2007.00367.x
Coleman FC, Williams SL (2002) Overexploiting marine ecosystem engineers: potential consequences for biodiversity. Trends Ecol Evol 17:40-44 doi:http://doi.org/10.1016/S0169-5347(01)02330-8
Enstipp MR et al. (2011) Energy expenditure of freely swimming adult green turtles (Chelonia mydas) and its link with body acceleration. J Exp Biol 214:4010-4020 doi:10.1242/jeb.062943 FitzSimmons, N. N. and Limpus, C. J. (2014). Marine Turtle Genetic Stocks of the Indo-Pacific: identifying boundaries and knowledge gaps. *Indian Ocean Marine Turtle Newsletter* **20**, 2-12.
Fuentes MMPB et al. (2015) Improving in-water estimates of marine turtle abundance by adjusting aerial survey counts for perception and availability biases. J Exp Mar Biol Ecol 471:77-83 doi:http://doi.org/10.1016/j.jembe.2015.05.003
Gyuris E, Limpus C (1986) Rapid Method for Immobilization and Collection of Sea-Turtle Muscle Biopsies for Electrophoresis. Wildl Res 13:333-334 doi:doi:10.1071/WR9860333
Hagihara R, Jones RE, Grech A, Lanyon JM, Sheppard JK, Marsh H (2014) Improving population estimates by quantifying diving and surfacing patterns: A dugong example. Mar Mamm Sci 30:348-366 doi:10.1111/mms.12041
Halsey LG, Jones TT, Jones DR, Liebsch N, Booth DT (2011) Measuring Energy Expenditure in Sub-Adult and Hatchling Sea Turtles via Accelerometry. PLoS One 6:e22311 doi:10.1371/journal.pone.0022311
Hamann M, Limpus CJ, Owens DW (2003) Reproductive cycles of males and females. In: Lutz PL, Muzick JA, Wyneken J (eds) The Biology of Sea Turtles, vol II. CRC Press, Boca Raton, pp 135-161
Hijmans RJ (2016) raster: geographic data analysis and modeling. R package v. 2.5-8. https://CRAN.R-project.org/package=raster. Accessed 24 November 2016
IUCN (2017) IUCN Red List of Threatened Species. Version 2016-3. www.iucnredlist.org (Accessed 13 April 2017)
Kahle D, Wickham H (2013) ggmap: Spatial Visualization with ggplot2. The R Journal 5:144-161
Limpus CJ (1992) The hawksbill turtle, *Eretmochelys imbricata*, in Queensland: population structure within a southern Great Barrier Reef feeding ground. Wildl Res 19:489-506 doi:http://dx.doi.org/10.1071/wr9920489
Limpus CJ, Carter D, Hamann M (2001) The green turtle, Chelonia mydas, in Queensland, Australia: the Bramble Cay rookery in the 1979-1980 breeding. Chelonian Conserv Biol v. 4, no. 1:p. 34-46
Limpus CJ, Limpus DJ (2001) The loggerhead turtle, *Caretta caretta*, in Queensland: breeding migrations and fidelity to a warm temperate feeding area. Chelonian Conserv Biol 4:142-153
Limpus CJ, Miller JD, Parmenter CJ, Limpus DJ (2003) The green turtle, Chelonia mydas, population of Raine Island and the northern Great Barrier Reef. Mem Queensl Mus 49:349-440
Limpus CJ, Miller JD, Parmenter CJ, Reimer D, Mclachlan N, Webb R (1992) Migration of green (*Chelonia mydas*) and loggerhead (*Caretta caretta*) turtles to and from eastern Australian rookeries. Wildl Res 19:347-358 doi:http://dx.doi.org/10.1071/WR9920347
Moran KL, Bjorndal KA (2007) Simulated green turtle grazing affects nutrient composition of the seagrass *Thalassia testudinum*. Mar Biol 150:1083-1092 doi:10.1007/s00227-006-0427-9
Office of the Queensland Chief Scientist (2015) Revised Queensland Science and Research Priorities. Brisbane

Plotkin P (2003) Adult migrations and habitat use. In: Lutz PL, Musick JA, Wyneken J (eds) The biology of sea turtles, vol II. CRC Press, Florida, pp 225-241
Prange HD, Jackson DC (1976) Ventilation, gas exchange and metabolic scaling of a sea turtle. Respir Physiol 27:369-377 doi:10.1016/0034-5687(76)90065-7
Queensland Government (2016) Queensland Tourism Investment Guide 2016. Brisbane
R Core Team (2017) R: a language and environment for statistical computing v. 3.3.3. https://www.r-project.org. Accessed 11 April 2017
Robinson D (2017) broom: convert statistical analysis objects into tidy data frames. R package v. 0.4.2. https://CRAN.R-project.org/package=broom. Accessed 11 April 2017
Schofield G, Hobson VJ, Fossette S, Lilley MKS, Katselidis KA, Hays GC (2010) Fidelity to foraging sites, consistency of migration routes and habitat modulation of home range by sea turtles. Divers Distrib 16:840-853 doi:http://dx.doi.org/10.1111/j.1472-4642.2010.00694.x
Shimada T (2017) SDLfilter: filtering satellite-derived locations. R package v. 0.2.3. https://github.com/TakahiroShimada/SDLfilter. Accessed 8 March 2017
Shimada T, Jones R, Limpus C, Groom R, Hamann M (2016a) Long-term and seasonal patterns of sea turtle home ranges in warm coastal foraging habitats: implications for conservation. Mar Ecol Prog Ser 562:163-179 doi:https://doi.org/10.3354/meps11972
Shimada T, Jones R, Limpus C, Hamann M (2012) Improving data retention and home range estimates by data-driven screening. Mar Ecol Prog Ser 457:171-180 doi:https://dx.doi.org/10.3354/meps09747
Shimada T, Limpus C, Jones R, Hazel J, Groom R, Hamann M (2016b) Sea turtles return home after intentional displacement from coastal foraging areas. Mar Biol 163:1-14 doi:http://dx.doi.org/10.1007/s00227-015-2771-0
Stan Development Team (2016a) RStan: the R interface to Stan. R package v. 2.14.1. http://mc-stan.org/. Accessed 11 April 2017
Stan Development Team (2016b) rstanarm: Bayesian applied regression modeling via Stan. R package v. 2.13.1. http://mc-stan.org/. Accessed 11 April 2017
Wallace BP et al. (2011) Global Conservation Priorities for Marine Turtles. PLoS One 6:e24510 doi:10.1371/journal.pone.0024510
Whitlock MC, Schluter D (2009) The analysis of biological data. Roberts and Company Publishers, Greenwood Village, CO
Wickham H (2009) ggplot2: Elegant Graphics for Data Analysis. Springer-Verlag, New York

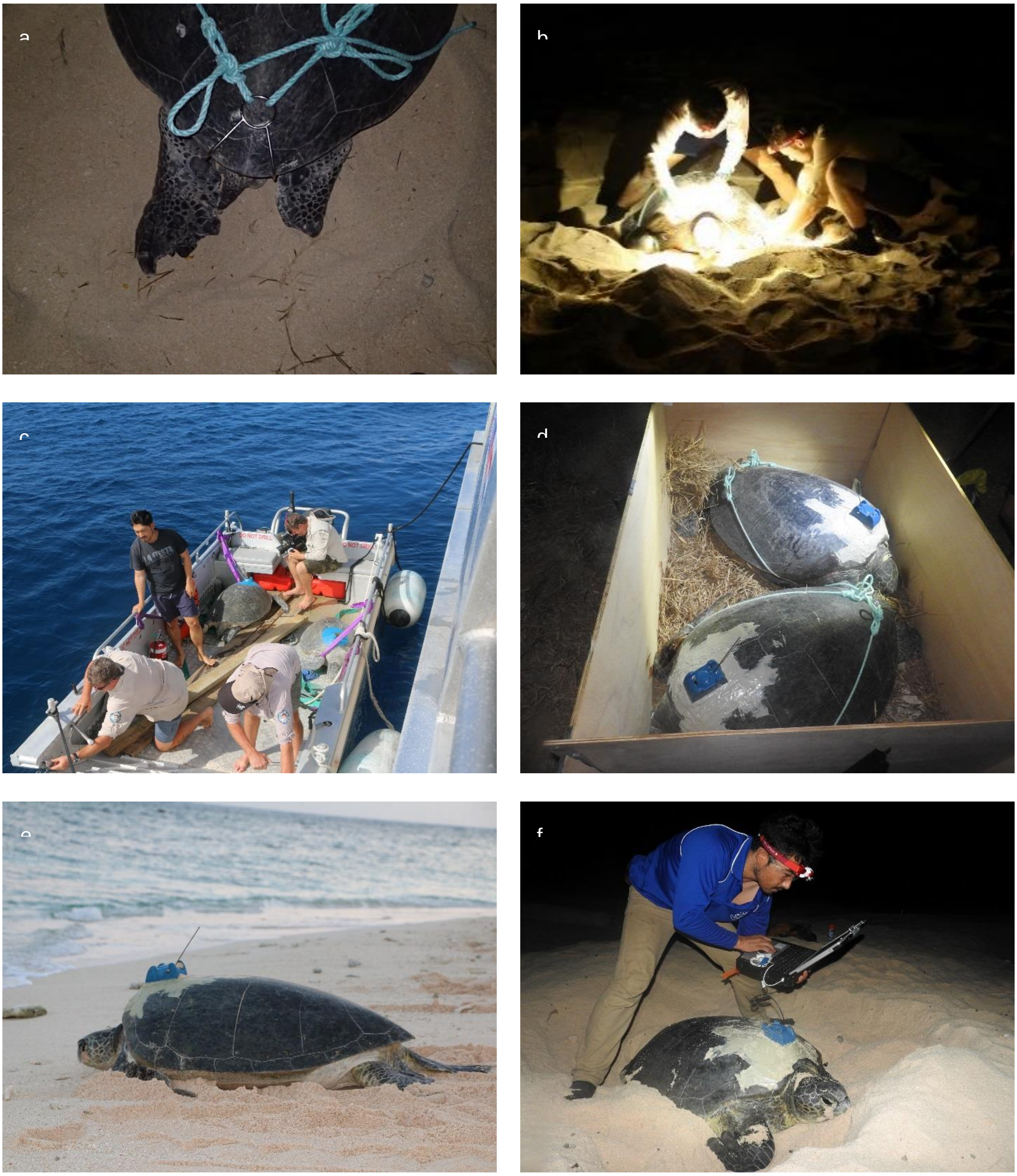

Figure 1. **a** Damaged right hind flipper of I759 next to her undamaged left hind flipper. **b** Deployment of a satellite-linked tag on a nesting green turtle at Raine Island. **c** Satellite-tag deployment on a barge in December 2015. **d** Satellite-tag deployment in a closure at Raine Island in November 2016. **e** A turtle deployed with a satellite tag leaving the beach. **f** Downloading archival data directly from the tag after her successful nesting. Photos (**b**, **d**, **f**) courtesy of the Queensland Parks and Wildlife Service.

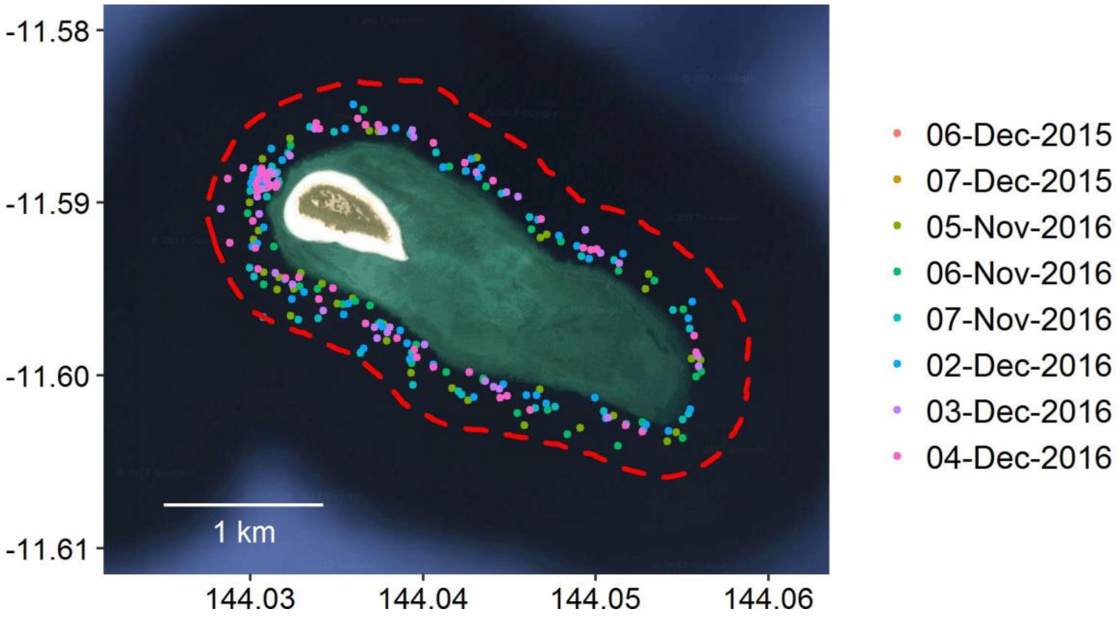

Figure 2. GPS locations of the survey boat (coloured dots) during the painted turtle count exercises in 2015 (2 survey days) and 2016 (6 survey days). Red dashed line outlines 350 m from the reef edge, which includes all the locations visited by the boat during the count exercises.

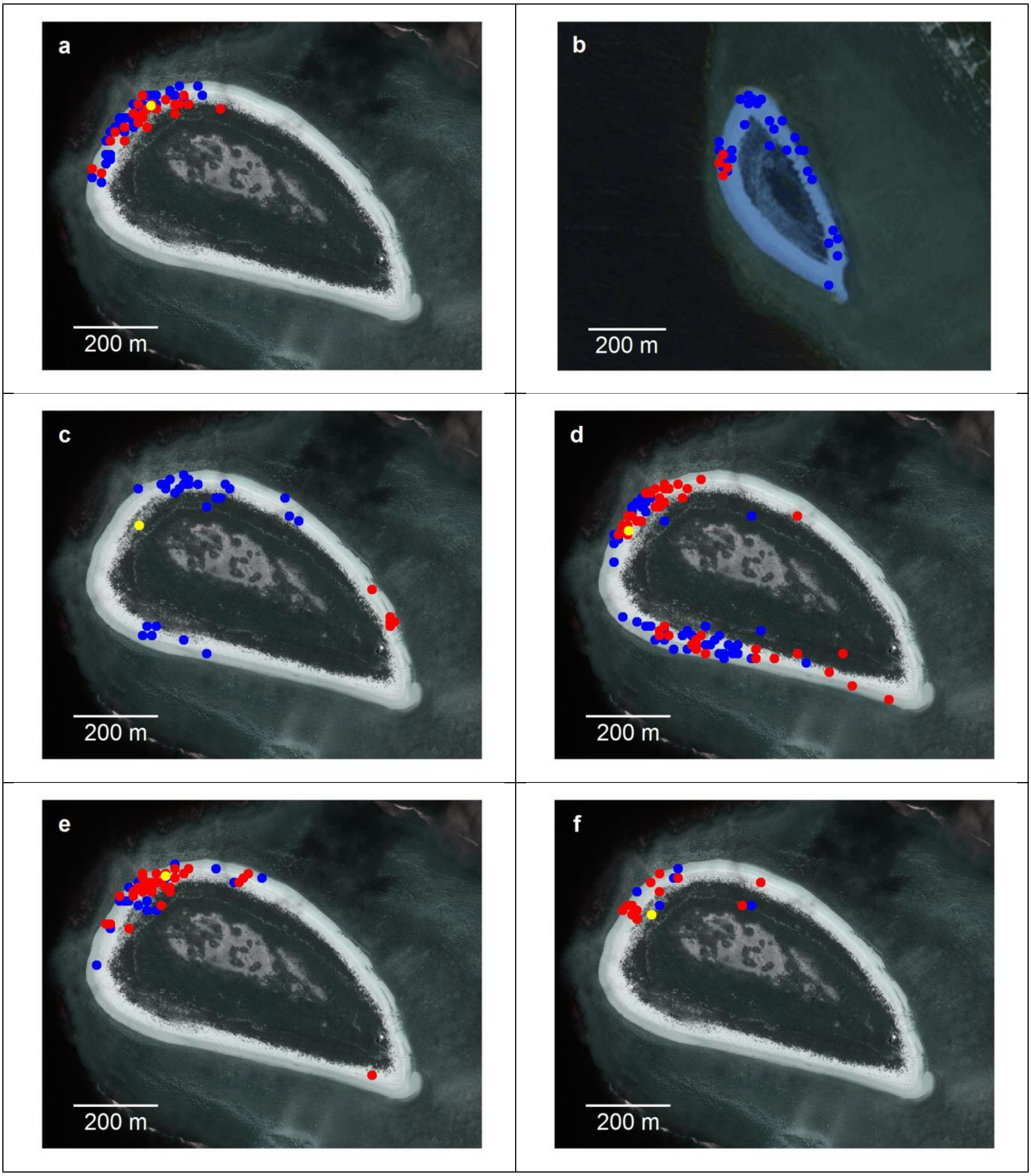

Figure 3. Satellite-derived locations of turtles during nesting or nesting attempt on Raine Island and Moulter Cay. Each turtle was captured at Raine Island during its nesting attempt (yellow circle), and released with a Fastloc GPS satellite tag deployed on the carapace. One turtle (I759) used both **a** Raine Island and **b** Moulter Cay, and the other four turtles were only observed at Raine Island (**c** I22154, **d** K74859, **e** T7159, **f** T90143). Location fixes are shown in red dots if they were obtained during a night when nesting likely occurred, or in blue dots if nesting attempt likely failed during the same night.

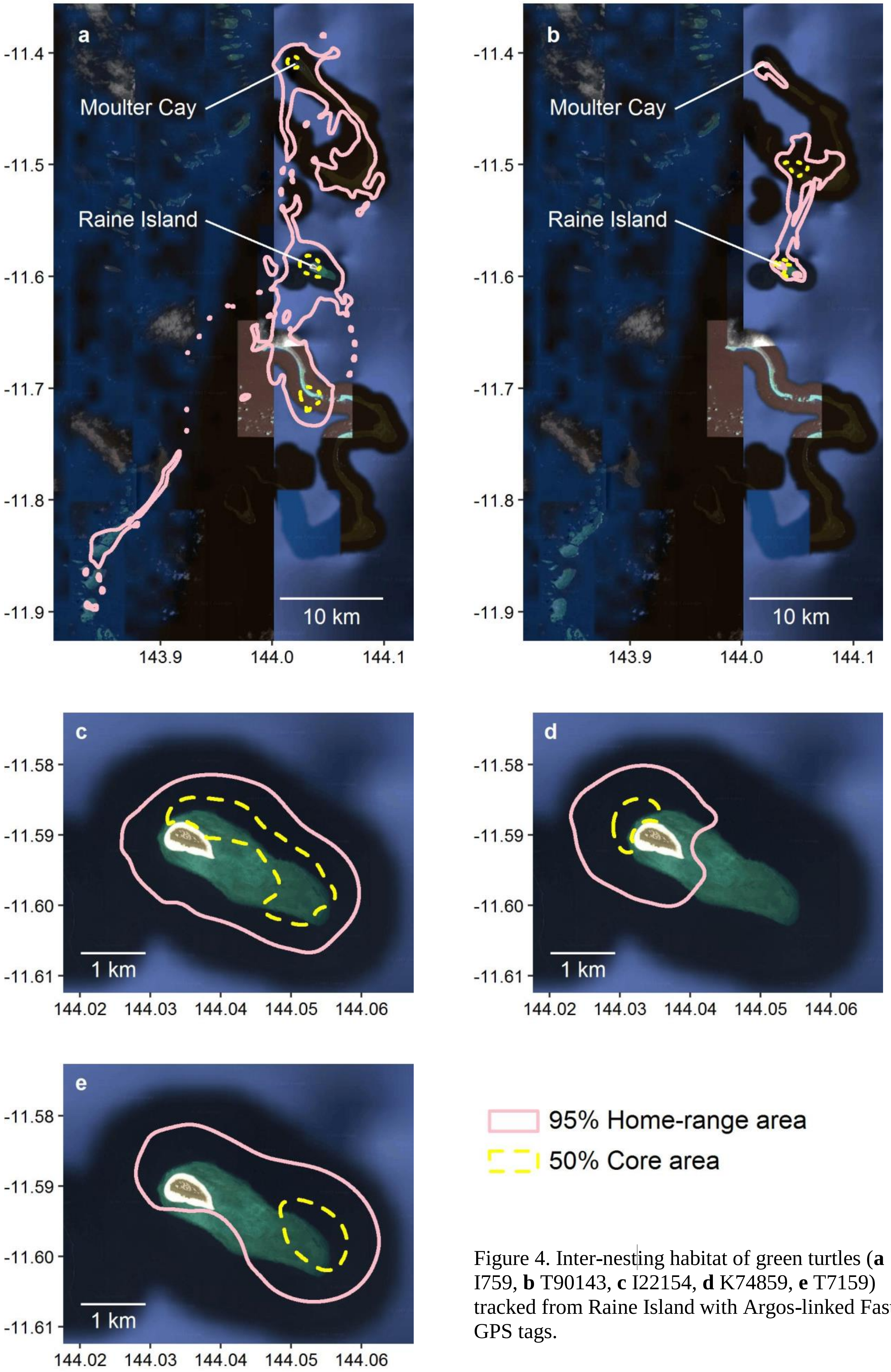

Figure 4. Inter-nesting habitat of green turtles (**a** I759, **b** T90143, **c** I22154, **d** K74859, **e** T7159) tracked from Raine Island with Argos-linked Fastloc GPS tags.

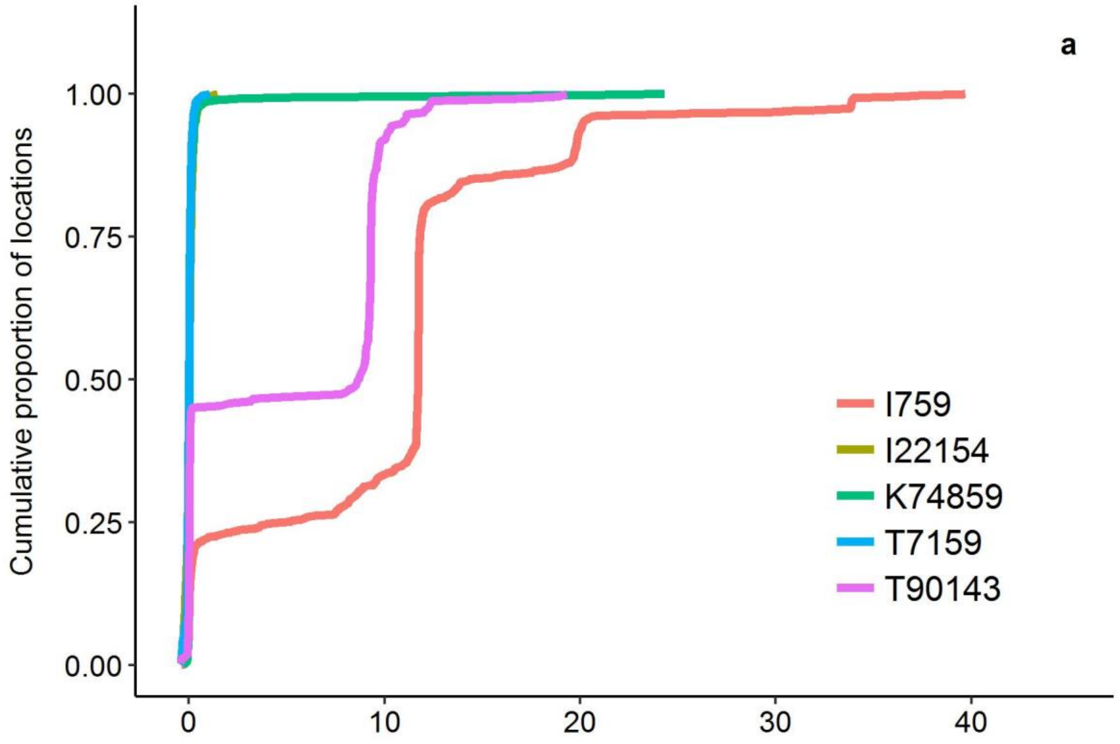

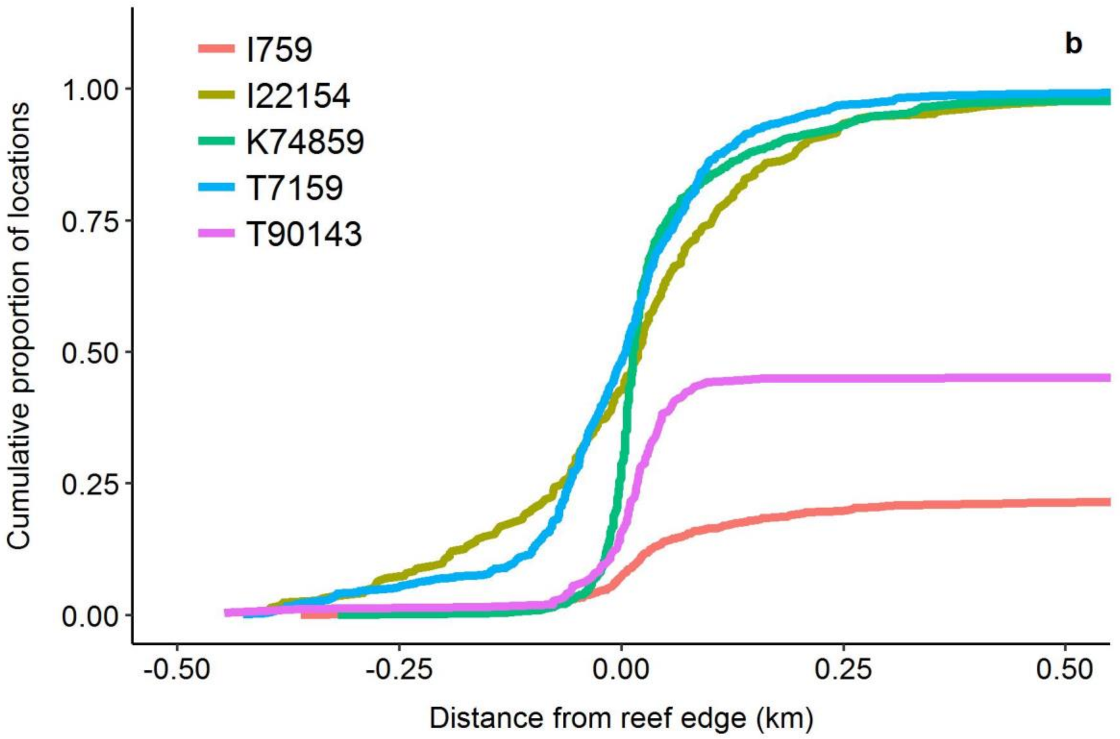

Figure 5. **a** Cumulative proportion of turtle locations relative to the distances from the reef edge around Raine Island, and **b** subset of the figure **a** zoomed into the distances between -0.5 and 0.5 km from the reef edge. Positive values are distances between turtle locations outside the reef and the reef edge. Negative values are distances between turtle locations on the reef and the reef edge.

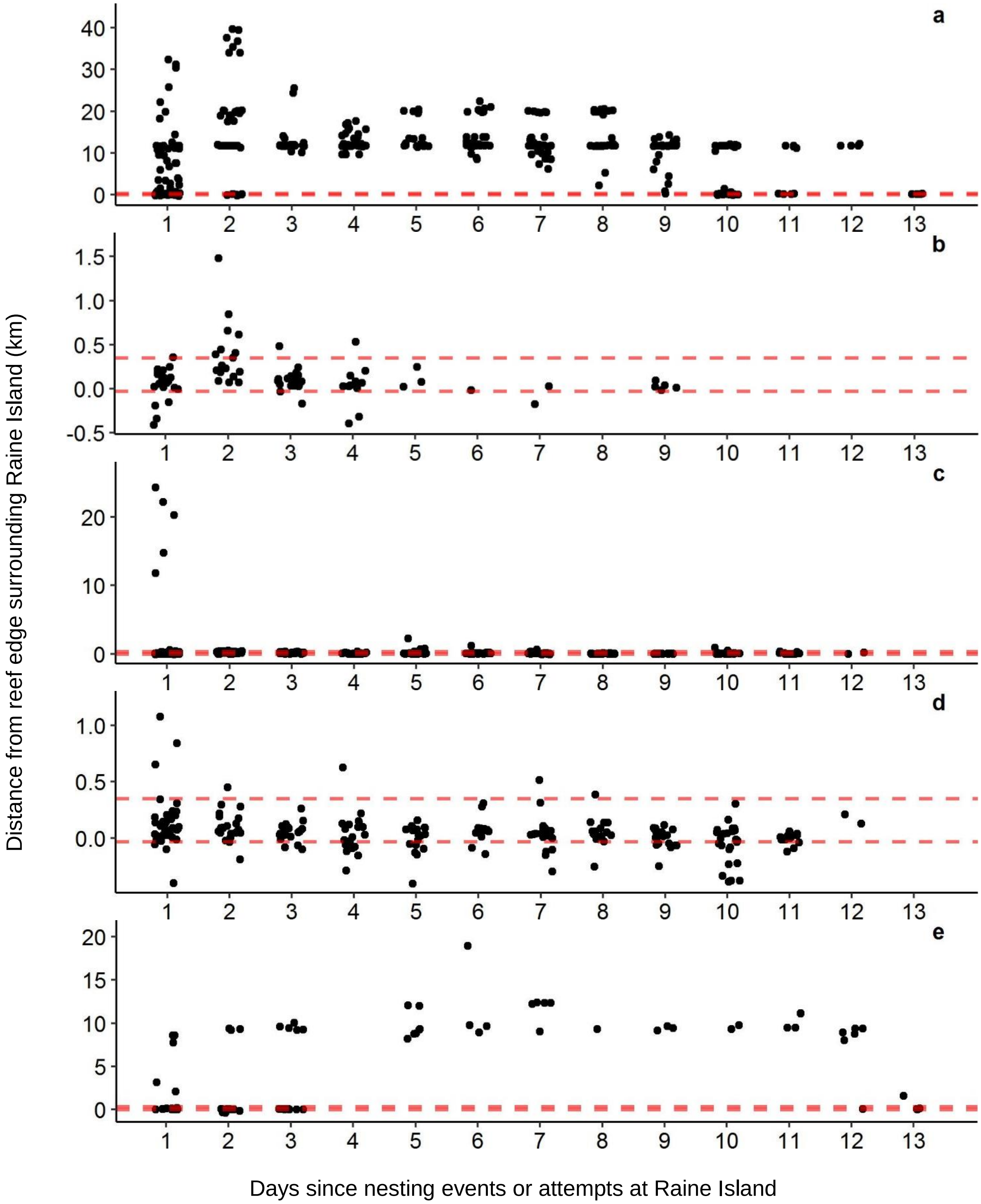

Figure 6. Distances between daytime turtle locations (**a** I759, **b** I22154, **c** K74859, **d** T7159, **e** T90143) and the reef edge of Raine Island during the days following their nesting events or attempts at Raine Island. Positive values are distances between turtle locations outside the reef and the reef edge. Negative values are distances between turtle locations on the reef and the reef edge. The red dashed lines indicate the range of the boat survey area (see Fig. 2).

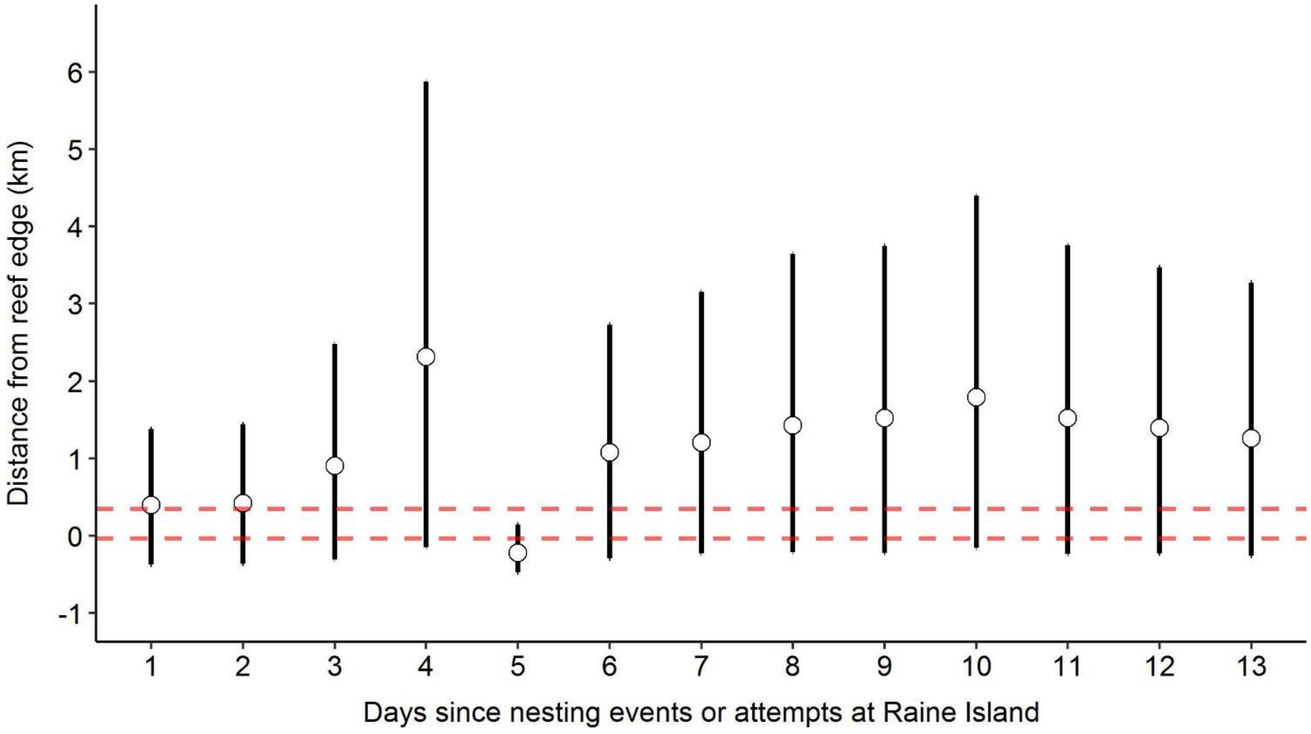

Figure 7. Estimated distances of the tracked turtles from the reef edge of Raine Island during the days following their nesting events or attempts at Raine Island. The circles are Bayesian generalised linear mixed model fits, with error bars denoting 95% credible intervals. Positive values are distances between turtle locations outside the reef and the reef edge. Negative values are distances between turtle locations on the reef and the reef edge. The red dotted lines indicate the range of the boat survey area (see Fig. 2). Only the turtle location data obtained during the daytime (8am to 5pm) were used for this analysis.

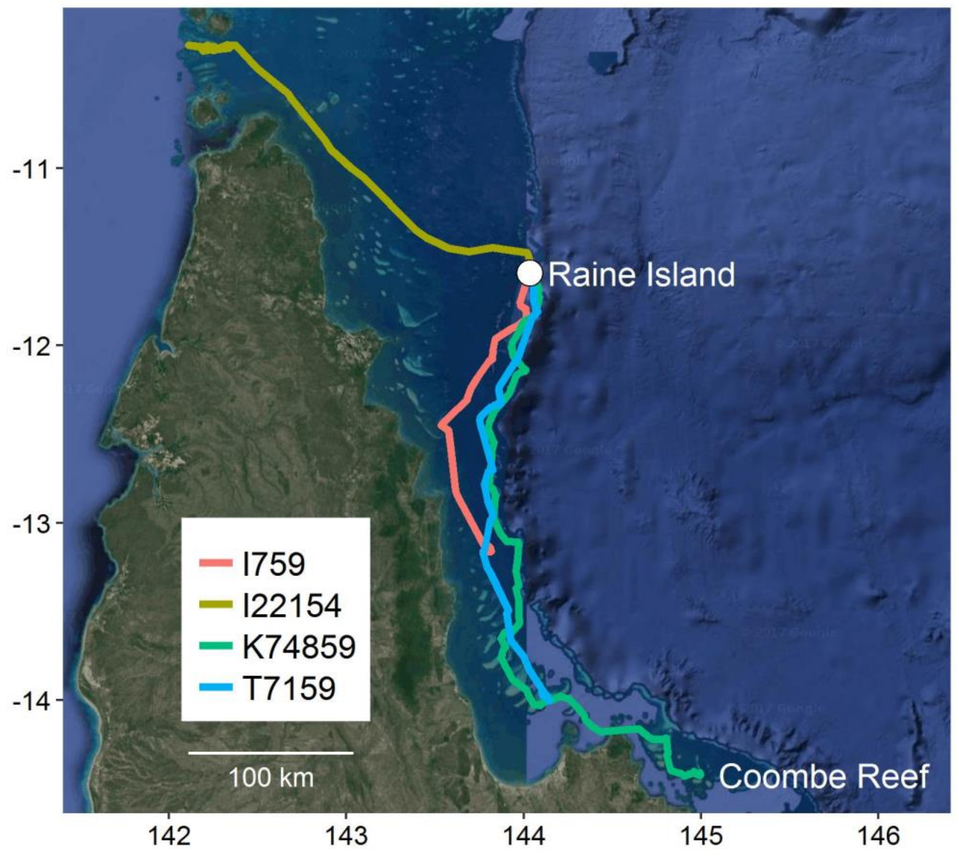

Figure 8. Post-nesting movements of four female green turtles tracked from Raine Island. The end point of each track is the foraging habitat of each turtle.

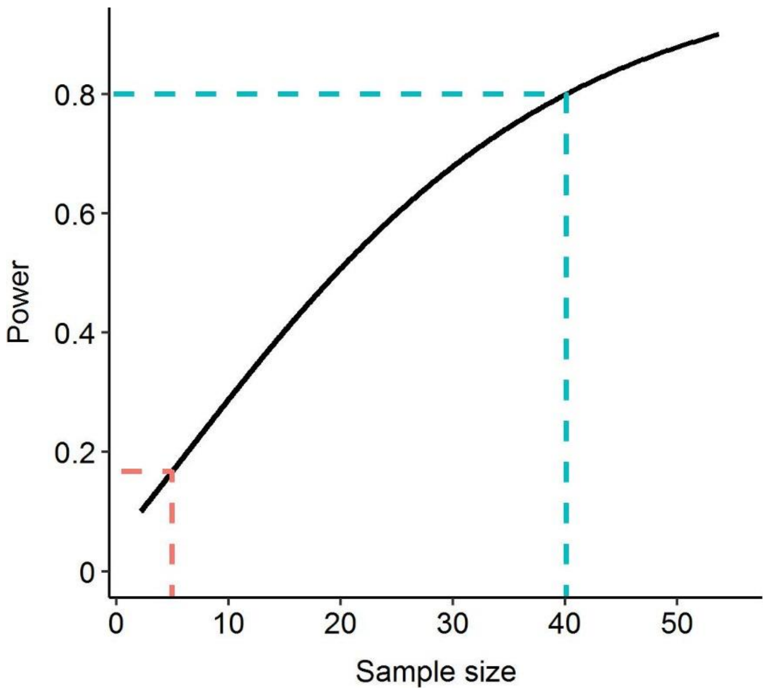

Figure 9. Power changes as a function of sample size (i.e. number of tracked turtles). Orange dashed line indicates the low power (0.17) with the current sample size (n = 5), and green dashed line indicates the high power (0.80) with an increased sample size (n = 40). See the main text for the parameters used for the power analysis.

Table 1. Summary data for female green turtles deployed with a Argos-linked Fastloc GPS tags at Raine Island in December 2015 and November 2016. CCL = curved carapace length.

Turtle ID	Argos ID	Capture history		
		Site	Month Year	CCL (cm)
I759	133766#	Raine Island	Dec 1992	109.0
		Raine Island	Dec 1997	109.5
		Raine Island	Dec 2002	109.7
		Raine Island	Nov 2016	109.9
I22154	133765	Raine Island	Nov 1993	103.0
		Raine Island	Dec 1997	103.4
		Raine Island	Dec 2001	102.1
		Raine Island	Dec 2015	103.2
K74859+	133763#	Coombe Reef	Jul 2006	104.7
		Raine Island	Dec 2006	104.8
		Raine Island	Nov 2016	105.5
T7159	133762#	Raine Island	Nov/Dec 1984	100.5
		Raine Island	Dec 1999	101.9
		Raine Island	Nov 2016	101.7
T90143	133764	Raine Island	Dec 1995	97.4
		Raine Island	Dec 2015	98.0

The satellite tags were still in operation as of 8 March 2017.

+The turtle returned to Coombe Reef following her breeding season at Raine Island, demonstrating over 10 years of site fidelity both to her nesting habitat (i.e. Raine Island) and to her foraging habitat (i.e. Coombe Reef).

Table 2. Nesting events or attempts (Nesting) estimated for five green turtles tracked during the summer of 2015/2016 and 2016/2017 at Raine Island and Moulter Cay. Estimation was made using data obtained with Argos-linked Fastloc GPS satellite tags (see Materials and methods - *Classification of nesting events and attempts*). As nesting activities occur across a night, the date of the earlier night was used here to represent the dates of each nesting activity. Duration of time (hours) spent on the beach was only obtained for two turtles (I22154 and T90143).

Turtle ID	Date	Duration	Location	Nesting
I759	2/11/2016	-	Raine	No lay+
	4/11/2016	-	Raine	No lay
	6/11/2016	-	Moulter	Lay
	17/11/2016	-	Raine	No lay
	18/11/2016	-	Raine	No lay
	19/11/2016	-	Raine	No lay
	20/11/2016	-	Moulter	No lay
	21/11/2016	-	Moulter	No lay
	22/11/2016	-	Moulter	No lay
	25/11/2016	-	Moulter	No lay
	26/11/2016	-	Moulter	No lay
	27/11/2016	-	Moulter	No lay
	28/11/2016	-	Raine	No lay
	29/11/2016	-	Raine	No lay
	30/11/2016	-	Raine	No lay
	1/12/2016	-	Raine	Lay
	12/12/2016	-	Raine	No lay
	13/12/2016	-	Raine	No lay
	14/12/2016	-	Raine	Lay
	24/12/2016	-	Raine	No lay
	25/12/2016	-	Raine	No lay
	26/12/2016	-	Raine	No lay
	27/12/2016	-	Raine	Lay
	6/01/2017	-	Raine	No lay
	7/01/2017	-	Raine	Lay
	17/01/2017	-	Raine	Lay
	27/01/2017	-	Raine	No lay
	28/01/2017	-	Raine	Lay
I22154	1/12/2015	-	Raine	No lay+
	4/12/2015	1.1	Raine	No lay*2
	5/12/2015	1.5	Raine	No lay
	5/12/2015	3.7	Raine	No lay
	9/12/2015	8.2	Raine	Lay
	18/12/2015	1.7	Raine	No lay
	19/12/2015	10.5	Raine	No lay
	20/12/2015	3.5	Raine	No lay
	21/12/2015	-	Raine	No lay
	22/12/2015	10.7	Raine	Lay

Table 2 continued.

Turtle ID	Date	Duration	Location	Nesting
K74859	3/11/2016	-	Raine	No lay+
	4/11/2016	-	Raine	No lay
	5/11/2016	-	Raine	Lay*1
	15/11/2016	-	Raine	No lay
	16/11/2016	-	Raine	No lay
	17/11/2016	-	Raine	No lay
	19/11/2016	-	Raine	Lay
	1/12/2016	-	Raine	No lay
	2/12/2016	-	Raine	No lay*2
	3/12/2016	-	Raine	No lay
	4/12/2016	-	Raine	No lay
	5/12/2016	-	Raine	No lay
	6/12/2016	-	Raine	Lay
	16/12/2016	-	Raine	No lay
	17/12/2016	-	Raine	No lay
	18/12/2016	-	Raine	Lay
	27/12/2016	-	Raine	No lay
	28/12/2016	-	Raine	Lay
	8/01/2017	-	Raine	Lay
	19/01/2017	-	Raine	Lay
	30/01/2017	-	Raine	No lay
	31/01/2017	-	Raine	Lay*2
T7159	3/11/2016	-	Raine	No lay+
	5/11/2016	-	Raine	Lay*2
	16/11/2016	-	Raine	No lay
	17/11/2016	-	Raine	Lay
	29/11/2016	-	Raine	No lay
	30/11/2016	-	Raine	No lay
	1/12/2016	-	Raine	No lay
	2/12/2016	-	Raine	Lay
	13/12/2016	-	Raine	Lay
	23/12/2016	-	Raine	No lay
	24/12/2016	-	Raine	Lay
	3/01/2017	-	Raine	No lay
	4/01/2017	-	Raine	Lay
	15/01/2017	-	Raine	No lay
	16/01/2017	-	Raine	Lay
	27/01/2017	-	Raine	No lay
	28/01/2017	-	Raine	Lay

Table 2 continued

Turtle ID	Date	Duration	Location	Nesting
T90143	1/12/2015	-	Raine	No lay+
	3/12/2015	-	Raine	No lay*2
	4/12/2015	1.9	Raine	Lay*1
	17/12/2015	3.1	Raine	No lay
	18/12/2015	4.0	Raine	No lay
	19/12/2015	5.5	Raine	No lay
	20/12/2015	4.9	Raine	Lay
	2/01/2016	3.7	Raine	Lay
	14/01/2016	6.1	Raine	Lay
	25/01/2016	0.0	Raine	No lay
	27/01/2016	4.5	Raine	Lay
	6/02/2016	-	Raine	Lay

+The turtle was captured for tag deployment before nesting.
*1The turtle was seen on the beach and successful nesting was observed.
*2The turtle was seen on the beach but nesting event was not observed.

Table 3. Summary of Table 2.

Turtle ID	Capture	Successful nesting	Unsuccessful nesting	Total nesting emergences	Rate of nesting success	Number of locations
I759	02/11/2016	7	20	27	0.26	151
I22154	01/12/2015	2	6	8	0.25	48
K74859	03/11/2016	8	13	21	0.38	126
T7159	03/11/2016	8	8	16	0.50	73
T90143	01/12/2015	6	4	10	0.60	20

Table 4. Summary of inter-nesting habitat use of five female green turtles tracked from Raine Island. The core and home range areas of their inter-nesting habitats are represented by 50% and 95% UDs respectively. Total days on the beach and nesting events were estimated using satellite-derived data. (see Materials and methods - *Classification of nesting events and attempts*).

Turtle ID	Release date	Tracking days	50% UD (km^2)	95% UD (km^2)	Number of locations
I759	03/11/2016	86	7.0	146.4	1178
I22154	02/12/2015	21	1.7	6.5	263
K74859	04/11/2016	89	0.3	3.3	1035
T7159	04/11/2016	86	0.8	5.6	668
T90143	02/12/2015	67	2.6	35.0	274

Table 5. Estimated probabilities of female green turtles at three different localities during the days following their nesting events or attempts at Raine Island. The localities are the boat survey area (see Fig. 2) and outside of the survey area either on or off the reef surrounding Raine Island. The probabilities are estimated for the daytime (8am to 5pm) when boat survey is conducted.

Days	Boat survey area	Outside of survey area	
		on the reef	off the reef
1	0.41	0.19	0.40
2	0.15	0.05	0.80
3	0.13	0.04	0.83
4	0.10	0.03	0.88
5	0.09	0.02	0.89
6	0.07	0.02	0.92
7	0.08	0.02	0.89
8	0.11	0.03	0.86
9	0.13	0.03	0.84
10	0.40	0.18	0.42
11	0.21	0.07	0.73
12	0.05	0.01	0.94
13	0.09	0.89	0.02

Table 6. Summary of post-breeding migration of four female green turtles tracked from Raine Island. Beeline distance is the straight-line distance between the first and last location of the track. , and travelling distance is the sum of distances between successive locations along the track. Minimum travelling speed was calculated using the duration of migration and travelling distance.

Turtle ID	Departure date	Tracking days	Beeline distance (km)	Travelling distance (km)	Travelling speed (km/h)	Number of locations
I759	28/01/2017	4	143	166	1.6	35
I22154	23/12/2015	12	236	255	0.9	21
K74859	01/02/2017	15	324	440	1.2	150
T7159	29/01/2017	11	266	304	1.1	56
T90143	-	-	-	-	-	-

Table 7. Summary of tracking data of four female green turtles at their foraging habitat.

Turtle ID	Arrival date	Tracking days	Number of locations
I759	02/02/2017	33	302
I22154	04/01/2016	89	119
K74859	16/02/2017	18	75
T7159	09/02/2017	25	92
T90143	-	-	-
